# Graph Representation Learning and Its Applications: A Survey

**DOI:** 10.3390/s23084168

**Published:** 2023-04-21

**Authors:** Van Thuy Hoang, Hyeon-Ju Jeon, Eun-Soon You, Yoewon Yoon, Sungyeop Jung, O-Joun Lee

**Affiliations:** 1 Department of Artificial Intelligence, The Catholic University of Korea, 43, Jibong-ro, Bucheon-si 14662, Gyeonggi-do, Republic of Korea; hoangvanthuy90@gmail.com (V.T.H.); tesniere@naver.com (E.-S.Y.); 2 Data Assimilation Group, Korea Institute of Atmospheric Prediction Systems (KIAPS), 35, Boramae-ro 5-gil, Dongjak-gu, Seoul 07071, Republic of Korea; hjjeon@kiaps.org; 3 Department of Social Welfare, Dongguk University, 30, Pildong-ro 1-gil, Jung-gu, Seoul 04620, Republic of Korea; yyoon@dongguk.edu; 4 Semiconductor Devices and Circuits Laboratory, Advanced Institute of Convergence Technology (AICT), Seoul National University, 145, Gwanggyo-ro, Yeongtong-gu, Suwon-si 16229, Gyeonggi-do, Republic of Korea; sungyeop.jung@snu.ac.kr

**Keywords:** graph embedding, graph representation learning, graph transformer, graph neural networks

## Abstract

Graphs are data structures that effectively represent relational data in the real world. Graph representation learning is a significant task since it could facilitate various downstream tasks, such as node classification, link prediction, etc. Graph representation learning aims to map graph entities to low-dimensional vectors while preserving graph structure and entity relationships. Over the decades, many models have been proposed for graph representation learning. This paper aims to show a comprehensive picture of graph representation learning models, including traditional and state-of-the-art models on various graphs in different geometric spaces. First, we begin with five types of graph embedding models: graph kernels, matrix factorization models, shallow models, deep-learning models, and non-Euclidean models. In addition, we also discuss graph transformer models and Gaussian embedding models. Second, we present practical applications of graph embedding models, from constructing graphs for specific domains to applying models to solve tasks. Finally, we discuss challenges for existing models and future research directions in detail. As a result, this paper provides a structured overview of the diversity of graph embedding models.

## 1. Introduction

Graphs are a common language for representing complex relational data, including social media, transportation system networks, and biological protein–protein networks [1,2]. Since most graph data are complex and high-dimensional, it is difficult for researchers to extract valuable knowledge. Therefore, processing graph data and transforming them into a form (fixed-dimensional vectors) is an important process that researchers can then apply to different downstream tasks [3]. The objective of graph representation learning is to obtain vector representations of graph entities (e.g., nodes, edges, subgraphs, etc.) to facilitate various downstream tasks, such as node classification [4], link prediction [5,6], community detection [7], etc. As a result, graph representation learning plays an important role since it could significantly promote the performance of the downstream tasks.

Representation of the graph data, however, is challenging and different from image and text data [8]. In textual data, words are linked together in a sentence, and they have a fixed position in that sentence. In image data, pixels are arranged on an ordered grid space and can be represented by a grid matrix. However, the nodes and edges in graphs are non-ordered and have their features. This leads to mapping graph entities to latent space while preserving the graph structure, and proximity relationships are challenging. In the case of a social network, a user can have many friends (neighbors) and various personal information, such as hometown, education level, and hobbies, which makes preserving the graph structure and properties significantly problematic. In addition, many real-world networks show dynamic behaviors in which graph structures and node features could be changed over time [9,10]. These could deliver challenges in capturing the graph structure and mapping graph entities into vector space.

**Figure 1:**
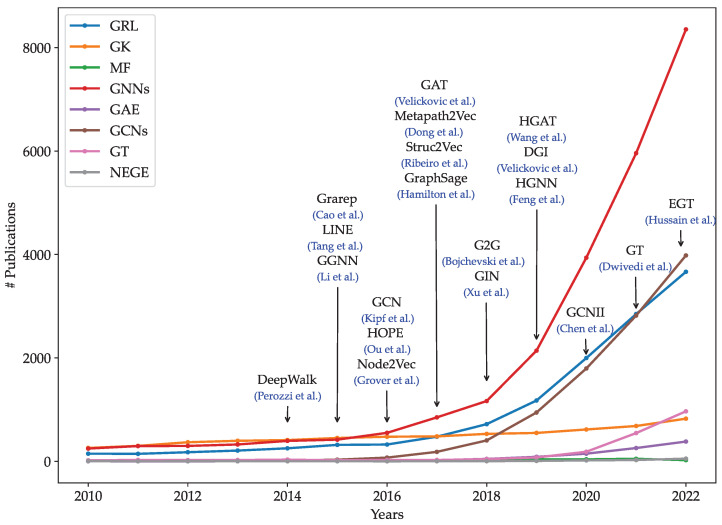
The popularity of graph representation learning models in the Scopus database. The line plot shows changes in the number of publications in different types of graph representation learning models from 2010 to 2022. The y-axis denotes the number of publications on the popularity of graph representation learning models over the years. There are seven keywords, including graph representation learning (GRL), graph kernels (GK), matrix factorization-based graph embedding (MF), graph neural networks (GNNs), graph autoencoder (GAE), graph convolution networks (GCNs), graph transformer (GT), and non-Euclidean graph embedding (NEGE). There are nineteen representative models, including DeepWalk [14], Grarep [15], LINE [16], GGCN [17], GCN [18], HOPE [5], Node2Vec [4], GAT [19], Metapath2Vec [20], Struc2Vec [21], GraphSage [22], G2G [23], GIN [24], HGAT [25], DGI [26], HGNN [27], GCNII [28], GT [29], and EGT [30].

Over decades, various graph representation learning models have been proposed to project graph entities into fixed-length vectors [11,12,13]. Graph embedding models are mainly divided into five main groups: graph kernels, matrix factorization models, shallow models, deep neural network models, and non-Euclidean models. Figure 1 presents the popularity of different graph representation learning models from 2010 to 2022. The number of graph representation learning studies increased considerably over the period of 12 years. Furthermore, there was significant growth in the frequency of research studies on graph neural networks, graph convolutional networks, and graph transformer models. In contrast, the number of studies in graph kernels, graph autoencoder, and matrix factorization-based models increased slightly over the period of 12 years. We obtained the frequency of academic publications including each keyword from Scopus (https://www.scopus.com (accessed on 16 April 2023)).

Historically, the first graph representation learning models were graph kernels. The idea of graph kernel methods perhaps comes from the most essential and well-known Weisfeiler–Lehman (WL) isomorphic testing in 1968 [31]. Graph kernels are kernel functions that aim to measure the similarity between graphs and their entities [32]. The main idea of graph kernels is to decompose original graphs into substructures and construct vector embeddings based on the substructure features. There are two main types of graph kernels: kernels for graphs and kernels on graphs. The former aims to measure the similarity between pairs of graphs, while the latter estimates the similarity between graph nodes. Several strategies to estimate the similarity of graph pairs have been proposed to represent various graph structures, such as graphlet kernels, random walk, and the shortest path, which started in the 2000s. Based on WL isomorphic testing, various graph kernels are built to compute the similarity of pairs of graph entities, such as WL kernels [31], WL subtree kernels [33,34,35], and random walks [36,37]. However, one of the limitations of graph kernels is the computational complexity when working with large-scale graphs since computing graph kernels is an NP-hard class.

Early models for graph representation learning primarily focused on matrix factorization methods, which are motivated by traditional techniques for dimensionality reduction in 2002 [38]. Several matrix factorization-based models have been proposed to handle large graphs with millions of nodes [39,40]. The objective of matrix factorization models is to decompose the proximity matrix into a product of small-sized matrices and then learn the embeddings that fit the proximity. Based on the ways to learn vector embeddings, there are two main lines of matrix factorization models: Laplacian eigenmaps and node proximity matrix factorization. Starting in the 2000s, Laplacian eigenmaps methods [41,42] aim to represent each node by Laplacian eigenvectors along with the first *k* eigenvalues. In contrast, the node proximity matrix factorization methods [5,15] aim to gain node embeddings by the singular value decomposition in 2015. Various proximity matrix factorization models have successfully handled large graphs and achieved great performance [15,43]. However, matrix factorization models suffer from capturing high-order proximity due to computational complexity when performing with high transition matrices.

In 2014 and 2016, early shallow models, DeepWalk [14] and Node2Vec [4] were proposed, which learn node embeddings based on shallow neural networks. Remarkably, the primary concept is to learn node embeddings by maximizing the neighborhood probability of target nodes using the skip-gram model started in the natural language processing area. The purpose of this strategy could then be optimized with SGD on neural network layers, thus reducing computational complexity. With this historic milestone, various models have been developed by improving multiple sampling strategies and training processes. Shallow models are the embedding models that aim to map graph entities to low-dimensional vectors by conducting an embedding lookup for each graph entity [3]. From this perspective, the embedding of node $v_{i}$ could be represented as $Z_{i}=Mx_{i}$, where *M* denotes an embedding matrix of all nodes and $x_{i}$ is a one-hot vector of node $v_{i}$. Various shallow models have been proposed to learn embeddings with different strategies to preserve graph structures and the similarity between graph entities. Structure-preservation models aim to preserve the structural connection between entities (e.g., DeepWalk [14], Node2Vec [4]). In 2015, Tang et al. [16] proposed the LINE model, a proximity reconstruction method that aims to preserve proximity between nodes in graphs. After that, various models have been proposed to preserve the node proximity with higher-order proximity and capture more global graph structure. However, most of the above models focus on transductive learning and ignore node features, which may have several limitations to practical applications.

Breakthroughs in deep learning have led to a new research perspective on applying deep neural networks to the graph domain. Since the 2000s, there have been several early models on GNNs designed to learn node embeddings based on neighborhood information using an aggregation mechanism [44,45]. Graph neural networks (GNNs) have shown a significant expressive capacity to represent graph embeddings in an inductive learning manner and solve the limitations of aforementioned shallow models [46,47]. Recurrent GNNs are also the first studies on GNNs based on recurrent neural network architecture [48,49] in 2005. These models aim to learn node embeddings via recurrent layers with the same weights in each hidden layer and run recursively until convergence. Several recurrent GNNs with different strategies have been proposed by the power of recurrent neural network architecture and the combinations with several sampling strategies. However, using the same weights at each hidden layer of the RGNN model may cause the model to be incapable of distinguishing the local and global structure. Since 2016, several graph autoencoder models have been proposed based on the original autoencoder architecture, which could learn complex graph structures by reconstructing the input graph structure [50,51]. The graph autoencoders comprise two main layers: encoder layers take the adjacency matrix as input and squeeze it to generate node embeddings, and decoder layers reconstruct the input data. By contrast, the idea of CGNNs is to use convolutional operators with different weights in each hidden layer, which are more efficient in capturing and distinguishing the local and global structures [18,52,53,54]. Many studies have been proposed with various variants of CGNNs, including spectral CGNNs [55,56,57] started in 2014, spatial CGNNs [22,24,52] started in 2016, and attentive CGNNs [19,58] started in 2017. Nevertheless, most GNNs suffer limitations such as over-smoothing problems and noise from neighbor nodes when stacking more GNN layers [59,60].

Motivated by transformer architecture started from natural language processing applications in 2017, several graph transformer models were proposed using the transformer architecture to the graph domain in 2019 [61,62]. Graph transformer models have shown competitive and superior performance against GNNs in learning complex graph structures [30,63]. Graph transformer models can be divided into three main groups: transformer for tree-like graphs, transformer with GNNs, and transformer with global self-attention. Early graph transformer models aim to learn tree-like graphs, which mainly aim at learning node embeddings in tree-like graphs where nodes are arranged hierarchically [64,65] since 2019. These models encode the node positions through their relative and absolute positional encoding in trees as constraints with root nodes and neighbor nodes at the same level. Second, several models leverage the power of GNNs as an auxiliary module in computing attention scores [66]. In addition, some models put GNN layers on top of the model to overcome the over-smoothing problem and make the model remember the local structure [61]. Most above graph transformer models adopt vanilla transformer architecture to learn embeddings that rely on multi-head self-attention. Third, several graph transformer models use a global self-attention mechanism to learn node embeddings, which implements self-attention independently and does not require constraints from the neighborhood [30,67]. These models work directly on input graphs and can capture the global structure with global self-attention.

Most of the above models learn embeddings in Euclidean space and represent graph entities as vector points in latent space. However, graphs in the real world could have complex structures and different forms, such that Euclidean space may be inadequate to represent the graph structure and ultimately lead to structural loss [68,69]. Early models learn complex graphs in non-Euclidean geometry by developing efficient algorithms for learning node embeddings based on manifold optimization [70] in 2017. Following the line, several models aim to represent graph data in non-Euclidean space and gain desirable results [68,69,71]. Two typical non-Euclidean spaces, including spherical and hyperbolic geometry, have their advantages. Spherical space could represent graph structures with large cycles, while hyperbolic space is suitable for hierarchical graph structures. Most non-Euclidean models aim to design an efficient algorithm for learning node embeddings since it is challenging to implement operators directly in non-Euclidean. Furthermore, to deal with uncertainty, several Gaussian graph models have been introduced to represent graph entities as density-based embeddings [23] started in 2016. Node embeddings could be defined as a continuous density mostly based on Gaussian distribution [72].

To the extent of our knowledge, no comparable paper in the literature focuses on a wide range of graph embedding models for static and dynamic graphs in different geometric spaces. Most current papers only presented specific approaches for graph representation learning. Wu et al. [8] focused on graph neural network models, which are presented as a section in this paper. Several surveys [13,73,74] summarized graph embedding models for various types of graphs, but they did not mention either graph transformer models or non-Euclidean models. From applying graph embedding models to practical applications, several papers only list the applications for specific and narrow tasks [12,75]. However, we discuss how graphs are constructed in specific applications and how graph embedding models are implemented in various domains.

This paper presents a comprehensive picture of graph embedding models in static and dynamic graphs in different geometric spaces. In particular, we recognize five general categories of models for addressing graph representation learning, including graph kernels, matrix factorization models, shallow models, deep neural network models, and non-Euclidean models. The contribution of this study can be categorized as follows:

- This paper presents a taxonomy of graph embedding models based on various algorithms and strategies.
- We provide readers with an in-depth analysis of an overview of graph embedding models with different types of graphs ranging from static to dynamic and from homogeneous to heterogeneous graphs.
- This paper presents graph transformer models, which have achieved remarkable results in a deeper understanding of graph structures in recent years.
- We cover applications of graph representation learning in various areas, from constructing graphs to applying models in specific tasks.
- We discuss the challenges and future directions of existing graph embedding models in detail.

Since abundant graph representation learning models have been proposed recently, we employed different approaches to find related studies. We built a search strategy by defining keywords and analyzing reliable sources. The list of keywords includes graph embedding, graph representation learning, graph neural networks, graph convolution, graph attention, graph transformer, graph embedding in non-Euclidean space, Gaussian graph embedding, and applications of graph embedding. We found related studies at famous top-tier conferences and journals such as AAAI, IJCAI, SIGKDD, ICML, WSDM, Nature Machine Intelligence, Pattern Recognition, Intelligent Systems with Applications, the Web, and so on.

The following sections of this paper are summarized as follows. Section 2 describes fundamental concepts and backgrounds related to graph representation learning. In Section 3, all the graph embedding models will be presented, such as graph kernels, matrix factorization models, shallow models, deep neural network models, and non-Euclidean models. Section 4 discusses a wide range of practical applications of graph embedding models in the real world. Section 5 summarizes the latest benchmarks, downstream tasks, evaluation metrics, and libraries. Challenges for existing graph embedding models and future research directions will be discussed in Section 6. The last section, Section 7 is the conclusion.

## 2. Problem Description

Graph representation learning aims to project the graph entities into low-dimensional vectors while preserving the graph structure and the proximity of entities in graphs. With the desire to map graph entities into vector space, it is necessary to model the graph in mathematical form. Therefore, we begin with several fundamental definitions of graphs. The list of standard notations used in this survey is detailed in Table 1. Mathematically, a graph *G* can be defined as follows:

**Definition** **1** (Graph [3]). *A graph is a discrete structure consisting of a set of nodes and the edges connecting those nodes. The graph can be described mathematically in the form: G=(V,E,A), where $V=\{v_{1},v_{2},\cdots ,v_{N}\}$ is the set of nodes, $E=\{\left(v_{i},v_{j}\right)|\left(v_{i},v_{j}\right)\in V\times V\}$ is the set of edges, and A is an adjacency matrix. A is a square matrix of size N×N where N is the number of nodes in graphs. This can be formulated as follows:*

* *

(1) $$\begin{array}{c} A=\left[\begin{array}{ccc} A_{11} & \cdots & A_{1N}\\ \vdots & \ddots & \vdots \\ A_{N1} & \cdots & A_{NN} \end{array}\right],\;A_{ij}=\left\{\begin{array}{cc} 1, & if\;e_{ij}\in E.\\ 0, & otherwise. \end{array}\right. \end{array},$$

*where $A_{ij}$ indicates adjacency between node $v_{i}$ and node $v_{j}$.*

When $A_{ij}$ is binary, the matrix *A* represents only the existence of connections between nodes. By extending the definition of matrix *A*, we could expand to abundant different types of graph *G*:

- Directed graph: When $A_{ij}=A_{ji}$ for any 1≤i, j≤n, then the graph *G* is called an undirected graph, and *G* is directed graph otherwise.
- Weighted graph: is a graph in which each edge is assigned a specific weight value. Therefore, the adjacency matrix could be presented as: $A_{ij}=w_{ij}$, where $w_{ij}\in R$ is the weight of the edge $e_{ij}$.
- Signed graph: When $A_{ij}\in \left[-\infty ,\infty \right]$, the graph *G* is called signature/signed graph. The graph *G* could have all positive signed edges when $A_{ij}>0$ for any 1≤i, j≤n, and *G* could have all negative signed edges otherwise.
- Attributed graph: A graph G=(V,E,A,X) is an attributed graph where *V*, *E* is the set of nodes and edges, respectively, and *X* is the matrix of node attributes with size n×d. Furthermore, we could also have the matrix *X* as the matrix of edge input attribute with size m×d where *m* is the number of edges $e_{ij}\in E$ for any 1≤i, j≤n.
- Hyper graph: A hyper graph *G* could be represented as G=(V,E,W), where *V* denotes the set of nodes and *E* denotes a set of hyperedge. Each hyperedge $e_{ij}$ can connect multiple nodes and is assigned a weight $w_{ij}\in W$. The hypergraph *G* could be represented by an incidence matrix *H* size $\left|V\right|\times \left|E\right|$ with entries $h(v_{i},v_{j})=1$ if $e_{ij}\in E$, and $h(v_{i},v_{j})=0$ otherwise.
- Heterogeneous graph: A heterogeneous graph is defined as G=(V,E,T,φ,ρ) where *V*, and *E* are the set of nodes and edges, respectively, φ is the mapping function: $\varphi :V\to T_{v}$, and the mapping function $\rho :E\to T_{e}$ with $T_{v}$, $T_{e}$ describe the set of node types and edge types, respectively, and $T=T_{v}$+ $T_{e}$ is the sum of the number of node types and edge types.

According to the definitions of graph G=(V,E) that have been represented mathematically above, the idea of graph embedding is to map graph entities into low-dimensional vectors with the number of dimensions *d* with d≪N. Mathematically, the graph embedding is formulated as follows:

**Definition** **2** (Graph embedding [14]). *Given a graph G=(V,E) where V is the set of nodes, and E is the set of edges, graph embedding is a projection function ϕ(·), where $\phi :V\to R^{d}$ (d≪|V|) and $k\left(v_{i},v_{j}\right)≃\langle \phi \left(v_{i}\right),\phi \left(v_{j}\right)\rangle$ describes the proximity of two nodes $v_{i}$ and $v_{j}$ in the graph and $\langle \phi \left(v_{i}\right),\phi \left(v_{j}\right)\rangle$ is the distance of two vectors $\phi (v_{i})$ and $\phi (v_{j})$ in the vector space.*

**Figure 2:**
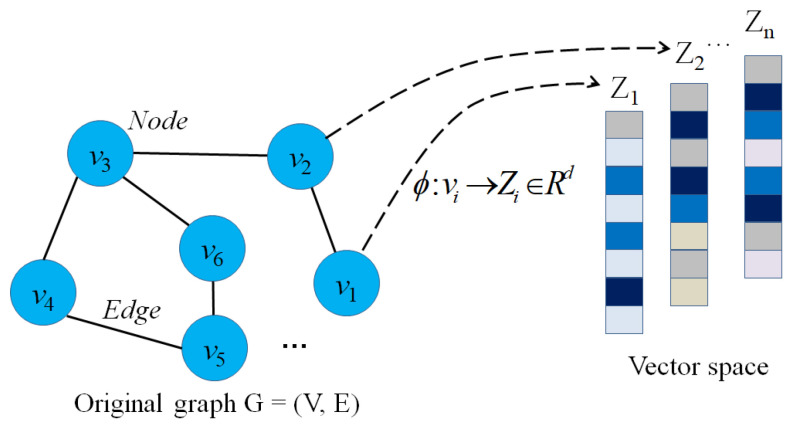
A comprehensive view of graph embedding. Given a spare, high-dimensional graph G=(V,E) where *V* and *E* denote the set of nodes and edges. Graph embedding learning aims to find a function ϕ that maps nodes from graph space to *d*-dimensional vector space with d≪|V|.

Graph representation learning aims to project graph entities into the vector space while preserving the graph structure and entity proximity. For example, if two nodes $v_{i}$ and $v_{j}$ in the graph *G* are connected directly, then in vector space, the distance between two vectors $\phi (v_{i})$ and $\phi (v_{j})$ must be minimal. Figure 2 shows an example of a graph embedding model that transforms nodes in a graph to low-dimensional vectors $\left(Z_{1}\;Z_{2}\;\cdots \;Z_{n}\right)$ in the vector space.

When mapping graph entities to latent space, preserving the proximity of graph entities is one of the most important factors in preserving the graph structure and the relationship between nodes. In other words, if two nodes $v_{i}$ and $v_{j}$ are connected or close in the graph, the distance between the two vectors $Z_{i}$ and $Z_{j}$ must be minimal in the vector space. Several models [16,76,77,78] aim to preserve *k*-order proximity between graph entities in vector space. Formally, the *k*-order proximity is defined as follows:

**Definition** **3** (*k*-order proximity [79]). *Given a graph G=(V,E) where V is the set of nodes, and E is the set of edges, k-order proximity describes the similarity of nodes with the distance captured from the k-hop in the graph G. When k=1, it is 1st-order proximity that captures the local pairwise proximity of two nodes in graphs. When k is higher, it could capture the global graph structure.*

There is another way to define graph embedding from the perspective of Encoder-Decoder architecture [3]. From this perspective, the task of the encoder part is to encode graph entities into low-dimensional vectors, and the decoder part tries to reconstruct the graph from the latent space. In the real world, many graphs show dynamic behaviors, including node and edge evolution, and feature evolution [80]. Dynamic graphs are found widely in many applications [81], such as social networks where connections between friends could be added or removed over time.

**Definition** **4** (Dynamic graph [80]). *A dynamic graph G is formed of three entities: $G=\left(V,E,T\right)$ where $V=\left\{V\left(t\right)\right\}$ is the group of node sets, E={E(t)} with t∈T is the group of edge sets over time span T, and T denotes the time span. From the statistic perspective, we could also consider a dynamic graph $G=\{G\left(t_{0}\right)\;G\left(t_{1}\right)\;\cdots \;G\left(t_{n}\right)\}$ as a collection of static graphs $G(t_{k})$ where $G\left(t_{k}\right)=\left(V\left(t_{k}\right),E\left(t_{k}\right)\right)$ denotes the static graph G at time $t_{k}$, and $V\left(t_{k}\right),E\left(t_{k}\right)$ denotes the set of nodes and set of edges at time $t_{k}$, respectively.*

**Figure 3:**
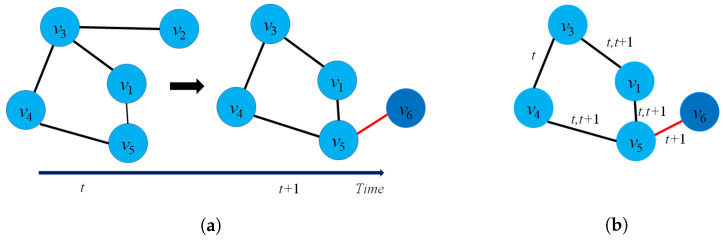
Methods for modeling dynamic graphs over time. (**a**) The representation of a dynamic graph by a series of snapshots; (**b**) The evolution of edges and nodes in the dynamic graph from time *t* to t+1. In (**a**), the graph *G* is the collection of G(t) (i.e., G={G(1),G(2),⋯,G(t)}) which *t* is the time span, and the entities of *G* change from time *t* to t+1. (**b**) depicts the evolution of edges in the same dynamic graph from (**a**) which each edge contains the series of the time spans from *t* to t+1. At time *t*, the graph has five nodes ($v_{1}$, $v_{2}$, $v_{3}$, $v_{4}$, $v_{5}$) and five edges $\left(e_{13}\;e_{15}\;e_{34}\;e_{45}\;e_{23}\right)$. However, at time t+1, the edge $e_{23}$ and node $v_{2}$ are removed, and a new node $v_{6}$, a new edge $e_{56}$ are added in the graph.

Figure 3a presents an example of dynamic graph representation. At time t+1, there are several changes in the graph G(t+1) such as: The edge $e_{23}$ will be removed, node $v_{6}$ will be added and new edge $e_{56}$. Casteigts et al. [80] proposed an alternative definition of a dynamic graph with five components: G=(V,E,T,ρ,ζ) where ρ:V×T→{0,1} describes the existence of each node at time *t*, and ζ:E×T→Ψ describes the existence of an edge at time *t*.

There is another way to model a dynamic graph based on the changes of the graph entities (edges, nodes) taking place on the graph G over a time span *t* or by an edge stream. From this perspective, a dynamic G could be modeled as $G=(V,E_{t},T)$ where $E_{t}$ presents the collection of edges of dynamic graph G at time *t*, and function $f:E\to R^{+}$ to map edges into integer numbers. It notices that all the edges at time *t* will have the same labels. Figure 3b describes the evolution of the edges of a graph from time (t) to (t+1).

**Definition** **5** (Dynamic graph embedding [82]). *Given a dynamic graph G=(V,E,T) where V={V(t)} is the group of node sets, and E={E(t)} is the group of edge sets over time span T, a dynamic graph embedding is a projection function ϕ(·), where $\phi \left(\cdot \right):G\times T\to R^{d}\times T$. T describes the time domain in latent space and T is the time span. When G is represented as the collection of snapshots: $G=\{G\left(t_{0}\right)\;G\left(t_{1}\right)\;\cdots \;G\left(t_{n}\right)\}$, then the projection function ϕ will be defined as: ϕ={ϕ(0)ϕ(1)⋯ϕ(n)} where ϕ(t) is the vector embedding of the graph G(t) at time t.*

There are two ways to represent a dynamic graph G, including a temporal dynamic graph embedding (changes over a period of time) and topological dynamic graph embedding (changes in the graph structure over time).

- Temporal dynamic graph embedding: A temporal dynamic embedding is a projection function ϕ(·), where $\phi_{t}:\;G_{t-k,t}\times T\to R^{d}\times T$ and $G_{t-k,t}=\left\{G_{t-k}\;G_{t-k+1}\;\cdots \;G_{t}\right\}$ describes the collection of graph G during time interval [t−k,t].
- Topological dynamic graph embedding: A topological dynamic graph embedding for graph G for nodes is a mapping function ϕ, where $\phi :V\times T\to R^{d}\times T$.

## 3. Graph Representation Learning Models

**Figure 4:**
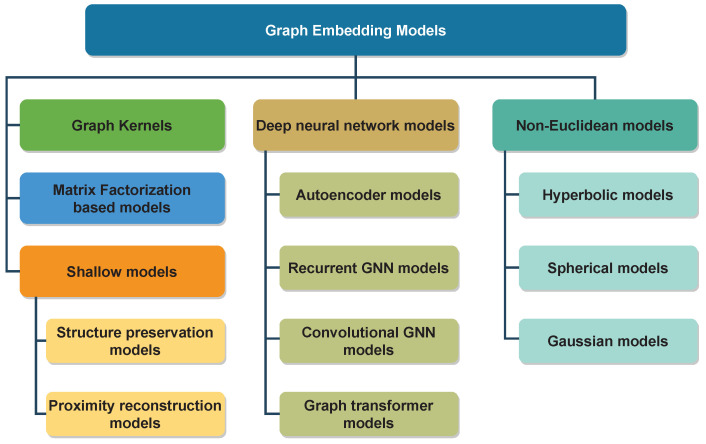
The proposed taxonomy for graph representation learning models.

This section presents a taxonomy of existing graph representation learning models in the literature. We categorize the existing graph embedding models into five main groups based on strategies to preserve graph structures and proximity of entities in graphs, including graph kernels, matrix factorization-based models, shallow models, deep neural network models, and non-Euclidean models. Figure 4 presents the proposed taxonomy of the graph representation learning models. Furthermore, we deliver open-source implementations of graph embedding models in Appendix A.

Graph kernels and matrix factorization-based models are one of the pioneer models for graph representation learning. Graph kernels are prevalent in learning graph embeddings using a deterministic mapping function in solving graph classification tasks [83,84,85]. There are two types of graph kernels: kernels for graphs, which aim to compare the similarity between graphs, and kernels on graphs aim to find the similarity between nodes in graphs. Second, matrix factorization-based models aim to represent the graph as matrices and gain embeddings by decomposing the matrices [5,86]. There are several strategies for factorization modeling, and most of these models aim to approximate high-order proximity between nodes. However, graph kernels and matrix factorization-based models suffer from computational complexity when handling large graphs and capturing high-order proximity.

Shallow models aim to construct an embedding matrix to transform each graph entity into vectors. We categorize shallow models into two main groups: structure preservation and proximity reconstruction. Structure-preservation strategies aim to conserve structural relationships between nodes in graphs [4,14,87]. Depending on specific tasks, several sampling strategies could be employed to capture graph structures, such as random walks [4,14], graphlets [88], motifs [89,90,91], etc. By contrast, the objective of the proximity reconstruction models is to preserve the proximity of nodes in graphs [16,92]. The proximity strategies can vary across different models based on their objectives. For example, the LINE model [16] aims to preserve 1st-order and 2nd-order proximity between nodes, while PALE [77] preserves pairwise similarities.

Graph neural networks have shown great performance in learning complex graph structures [18,50]. GNNs can be categorized into three main groups: graph autoencoder [50,51], recurrent GNNs [17,93], and convolutional GNNs. Graph autoencoders and recurrent GNNs are mostly pioneer studies of GNNs based on autoencoder architecture and recurrent neural networks, respectively. Graph autoencoders are composed of an encoder layer and a decoder layer. The encoder layer aims to compress a proximity graph matrix to vector embeddings, and the decoder layer reconstructs the proximity matrix. Most graph autoencoder models employ multilayer perceptron-based layers or recurrent GNNs as the core of autoencoder architecture. Recurrent GNNs aim to learn node embeddings based on recurrent neural network architecture in which connections between neurons can make a cycle. Therefore, earlier RGNNs mainly aimed to learn embeddings on directed acyclic graphs [94]. Recurrent GNNs employ the same weights in all hidden layers to capture local and global structures. Recently, convolutional GNNs have been much more efficient and can gain outstanding performance compared to RGNNs. The main difference between RGNNs and CGNNs is that CGNNs use different weights in each hidden layer, which could distinguish local and global structures. Various CGNN models have been proposed and mainly fall into two categories: spectral CGNNs, and spatial CGNNs [22,52,95]. Spectral CGNNs aim to transform graph data to the frequency domain and learn node embeddings in this domain [56,96]. By contrast, spatial CGNNs work directly on the graph using convolutional filters [53,54]. By staking multiple GNN layers, the models could learn node embeddings more efficiently and capture higher-order structural information [97,98]. However, stacking many layers could cause the over-smoothing problem, which most GNNs have not fully solved in a whole extent.

Recently, several models have enabled transformer architecture to learn graph structures which gain significant results compared to other deep-learning models [30,46,99]. We categorize graph transformer models into three main groups: transformer for tree-like graphs [64,65], transformer with GNNs [99,100], and transformer with global self-attention [30,67]. Different types of graph transformer models aim to handle distinct types of graphs. The transformer for tree-like graphs aims to learn node embeddings in tree-like hierarchical graphs [64,65,101]. The hierarchical relationships from the target nodes to their parents and neighbors are presented as absolute and relative positional encoding, respectively. Several graph transformer models employ the message-passing mechanism from GNNs as an auxiliary module in computing the attention score matrix [61,100]. GNN layers can be used to aggregate information as input to graph transformer models or put on top of the model, which aims to preserve local structures. In addition, some graph transformer models can directly process graph data without support from GNN layers [30,67]. These models implement a global self-attention to learn local and global structures in a graph input without neighborhood constraints.

Most existing graph embedding models aim to learn embeddings in Euclidean space, which may not deliver good geometric representations and metrics. Recent studies have shown that non-Euclidean spaces are more suitable for representing complex graph structures. The non-Euclidean models could be categorized as hyperbolic, spherical, and Gaussian. Hyperbolic and spherical space are two types of non-Euclidean geometry that could represent different graph structures. Hyperbolic space [102] is more suitable for representing hierarchical graph structures that follow the power law, while the power of spherical space is to represent large circular graph structures [103]. Moreover, since the information about the embedding space is unknown and uncertain, several models aim at learning node embeddings as Gaussian distribution [23,104].

### 3.1. Graph Kernels

Graph kernels aim to compare graphs or their substructures (e.g., nodes, subgraphs, and edges) by measuring their similarity [105]. The problem of measuring the similarity of graphs is, therefore, at the core of learning graphs in an unsupervised manner. Measuring the similarity of large graphs is problematic since the graph isomorphism problem is assigned to the NP (nondeterministic polynomial time) class. However, it is an NP-complete for subgraphs isomorphism problem. Table 2 describes a summary of graph kernel models.

Kernel methods applied to the graph embedding problem can be understood in two forms, including the isomorphism testing of *N* graphs (kernels for graphs) and embedding entities of graphs to Hilbert space (kernels on graphs).

- Kernels for graphs: Kernels for graphs aim to measure the similarity between graphs. The similarity between the two graphs (isomorphism) could be explained as follows: Given two undirected graphs $G_{1}=\left(V_{1}\;E_{1}\right)$ and $G_{2}=\left(V_{2}\;E_{2}\right)$, $G_{1}$ and $G_{2}$ are isomorphic if they exist a bimodal mapping function $\phi :V_{1}\to V_{2}$ such that $\forall a\;b\in V_{1}$, *a* and *b* are contiguous on $G_{1}$ if ϕ(a) and ϕ(b) are contiguous on $G_{2}$.
- Kernels on graphs: To embed nodes in graphs, kernel methods refer to finding a function that maps pairs of nodes to latent space using particular similarity measures. Formally, graph kernels could be defined as: Given a graph G=(V,E), a function K=V×V→R is a kernel on *G* if there is a mapping function ϕ:V→H such that $K\left(v_{i}\;v_{j}\right)=\langle \phi \left(v_{i}\right)\;\phi \left(v_{j}\right)\rangle$ for any node pairs $\left(v_{i}\;v_{j}\right)$.

There are several strategies to measure the similarity of pairs of graphs, such as graphlet kernels, WL kernels, random walk, and shortest paths [31,83]. Among the kernel methods, graphlet kernels are one of the simple kernels that could measure the similarity between graphs by counting subgraphs with a limited size *k* [83,106]. For instance, Shervashidze et al. [83] introduced a graphlet kernel with the main idea of finding the graph feature by counting the number of different graphlets in graphs. Formally, given an unlabeled graph *G*, a graphlet list $V_{k}=\left(G_{1}+G_{2}+\cdots +G_{n_{k}}\right)$ is the set of the graphlets with size *k* where $n_{k}$ depicts the number of graphlets. The graphlet kernel for two unlabeled graphs *G* and $G^{\prime }$ could be defined as:

(2) $$K\left(G,G^{\prime }\right)=\langle \phi \left(G\right),\phi \left(G^{\prime }\right)\rangle ,$$

where $\phi^{G}$ and $\phi^{G^{\prime }}$ are vectors that depict the number of graphlets in a $G_{i}$ and $G_{i}^{\prime }$, respectively. By counting all graphlets with size *k* for a graph, the computation time is expensive by the enumeration $n^{k}$ with *n* depicts the number of nodes in *G*. One of the practical solutions to overcome this limitation is to design the feature ${\phi_{i}}^{G}$ more effectively, called Weisfeiler–Lehman.

**Figure 5:**
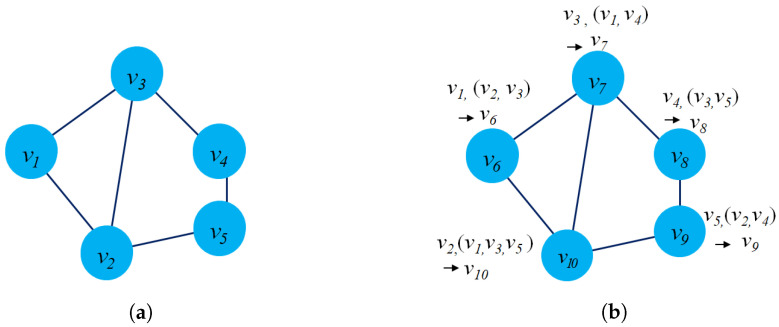
The Weisfeiler–Lehman isomorphism test. (**a**) Original labels, i=0; (**b**) Relabeled labels, i=1. There are two interactions of WL relabeling for the graph with five nodes $\left\{v_{1},v_{2},v_{3},v_{4},v_{5}\right\}$. In (**a**), labels of nodes are initialized consisting of 5 nodes. In (**b**), in the first iteration, new labels of the nodes will be reassigned and calculated based on the connection information to its adjacent nodes. For example, node $v_{1}$ is adjacent to node $v_{2}$ and node $v_{3}$, therefore the new label of $v_{1}$ is calculated as $\left\{v_{1},\langle v_{2},v_{3}\rangle \right\}$ and resigned as new label $v_{6}$. The same steps are repeated until a steady state for the nodes is reached.

Weisfeiler–Lehman (WL) test [31] is considered to be a traditional strategy to test the homomorphism of two graphs using color refinements. Figure 5 presents the main idea of the WL homomorphism test for two graphs in detail. By updating node labels, all the structure information of nodes in graphs could be stored at each node, including both local and global information, depending on the number of iterations. We can then compute histograms or other summary statistics over these labels as a vector representation for graphs.

Several models improved the idea from WL isomorphism test [34,84]. The concept of the WL isomorphism test inspired various GNN models later, which aim to be expressive as powerful as the WL test to distinguish different graph structures. Shervashidze et al. [33] presented three instances of WL kernels, including the WL subtree kernel, WL edge kernel, and WL shortest-path kernel with an enrichment strategy for labels. The key idea of [33] is to represent a graph *G* as WL sequences with the height of *h*. The WL sequences of two graphs *G* and $G^{\prime }$ can be defined as:

(3) $$\begin{array}{cc} \; & k_{WL}^{\left(h\right)}\left(G,G^{\prime }\right)=k\left(G_{0},G_{0}^{\prime }\right)+k\left(G_{1},G_{1}^{\prime }\right)+\cdots +k\left(G_{h},G_{h}^{\prime }\right)\; \end{array}$$

where $k\left(G_{i},G_{i}^{\prime }\right)=\langle \phi \left(G_{i}\right),\phi \left(G_{i}^{\prime }\right)\rangle$. For *N* graphs, the WL subtree kernel could be computed in a runtime of $O(Nhm+N^{2}hn)$, where *h* and *m* are the numbers of interactions and edges in *G*, respectively. Therefore, the algorithm could capture more information about the graph *G* after *h* interactions and compare graphs at different levels.

However, the vanilla WL isomorphism test requires massive resources since the methods are an NP-hard class. Following the WL isomorphism idea, Morris et al. [34] presented a set of *k*-set forms $V{\left(G\right)}_{k}$ and built a local and global neighborhood of the k-sets. Instead of working on each node in graphs, the models calculate and update the labels based on the k-set. The feature vectors of graph *G* then could be calculated by counting the number of occurrences of k-sets. Several models [84,114] improved the Wasserstein distance based on the WL isomorphism test, and the models could estimate weights of subtree patterns before the kernel construction [35]. Several models adopted a random-walk sampling strategy to capture the graph structure that could help reduce the computational complexity to handle large graphs [36,37,85,107].

However, the above methods only focus on homogeneous graphs in which nodes do not have side information. In the real world, graph nodes could contain labels and attributes and change over time, making it challenging to learn node embeddings. Several models have been proposed with slight variations from the traditional WL isomorphism test and random walk methods [109,110,111,112,113]. For example, Borgwardt et al. [109] presented random-walk sampling on attributed edges to capture the graph structure. Since existing kernel models primarily work on small-scale graphs or a subset of graphs, improving similarity based on shortest paths could achieve better computational efficiency for graph kernels in polynomial time. An all-paths kernel *K* could be defined as:

(4) $$K\left(P\left(G_{1}\right),P\left(G_{2}\right)\right)=\sum_{p_{1}\in P\left(G_{1}\right)}\sum_{p_{2}\in P\left(G_{2}\right)}k_{path}\left(p_{1},p_{2}\right),$$

where $P(G_{1})$ and $P(G_{2})$ are the set of random-walk paths in $G_{1}$ and $G_{2}$, respectively, and $k_{path}\left(p_{1},p_{2}\right)$ depicts a positive definite kernel on two paths $p_{1}$ and $p_{2}$. The model then applied Floyd–Warshall algorithm [115] to find *k* shortest-path kernels in graphs. One of the disadvantages of this model is the runtime complexity, which is about $O(k\times n^{4})$, where *n* depicts the number of nodes in graphs. Morris et al. [108] introduced a variation of the WL subtree kernel for attributed graphs by improving existing shortest-path kernels. The key idea of this model is to use a hash function that maps continuous attributes to label codes, and then it normalizes the discrete label codes.

To sum up, graph kernels are effective models and bring several advantages:

- Coverage: The graph kernels are one of the most useful functions to measure the similarity between graph entities by performing several strategies to find a kernel in graphs. This could be seen as a generalization of the traditional statistical methods [116].
- Efficiency: Several kernel tricks have been proposed to reduce the computational cost of kernel methods on graphs [117]. Kernel tricks could reduce the number of spatial dimensions and computational complexity on substructures while still providing efficient kernels.

Although kernel methods have several advantages, several disadvantages make the kernels difficult to scale:

- Missing entities: Most kernel models could not learn node embeddings for new nodes. In the real world, graphs are dynamic, and their entities could evolve. Therefore, the graph kernels must re-learn graphs every time a new node is added, which is time-consuming and difficult to apply in practice.
- Dealing with weights: Most graph kernel models do not consider the weighted edges, which could lead to structural information loss. This could reduce the possibility of graph representation in the hidden space.
- Computational complexity: Graph kernels are an NP-hard class [109]. Although several kernel-based models aim to reduce the computational time by considering the distribution of substructures, this may increase the complexity and reduce the ability to capture the global structure.

Although the graph kernels delivered good results when working with small graphs, they remain limitations when working with large and complex graphs [118]. To address the issue, matrix factorization-based models could bring far more advantages to learning node embeddings by decomposing the large original graphs into small-sized components. Therefore, we discuss matrix factorization-based models for learning node embeddings in the next section.

### 3.2. Matrix Factorization-Based Models

Matrix factorization aims to reduce the high-dimensional matrix that describes graphs (e.g., adjacency matrix, Laplacian matrix) into a low-dimensional space. Several well-known decomposition models (e.g., SVD, PCA, etc.) are widely applied in graph representation learning and recommendation system problems. Table 3 and Table 4 present matrix factorization-based models for static and dynamic graphs, respectively. Based on the strategy to preserve the graph structures, matrix factorization models could be categorized into two main groups: graph embedding Laplacian eigenmaps and node proximity matrix factorization.

- The Laplacian eigenmaps: To learn representations of a graph G=(V,E), these approaches first represent *G* as a Laplacian matrix *L* where L=D−A and *D* is the degree matrix [41]. In the matrix *L*, the positive values depict the degree of nodes, and negative values are the weights of the edges. The matrix *L* could be decomposed to find the smallest number from eigenvalues which are considered node embeddings. The optimal node embedding $Z^{*}$, therefore, could be computed using an objective function:
  (5) $$Z^{*}=\underset{Z}{\arg\min}\;Z^{⊺}LZ\;.$$
- Node proximity matrix factorization: The objective of these models is to decompose node proximity matrix into small-sized matrices directly. In other words, the proximity of nodes in graphs will be preserved in the latent space. Formally, given a proximity matrix *M*, the models try to optimize the distance between two pair nodes $v_{i}$ and $v_{j}$, which could be defined as:
  (6) $$Z^{*}=\underset{Z}{\arg\min}\;∥M_{ij}-Z_{i}{Z_{j}}^{T}∥\;.$$

Hofmann et al. [119] proposed an MSDC (Multidimensional Scaling and Data Clustering) model based on matrix factorization. The key idea of MSDC is to represent data points as a bipartite graph and then learn node embeddings based on node similarity in the graph. This method requires a symmetric proximity matrix $M\in R^{N\times N}$ as input and learns a latent representation of the data in Euclidean space by minimizing the loss that could be defined as:

(7) $$Z^{*}=\underset{Z}{\arg\min}\;\frac{1}{2|V|}{\sum_{(v_{i},v_{j})\in E}\left[{\left|Z_{i}-Z_{j}\right|}^{2}-M_{ij}\right]}^{2}\;.$$

However, the limitation of the MSDC model is that the model focuses only on the pairwise nodes, which cannot capture the global graph structure. Furthermore, the model investigated the proximity of all the data points in the graph, which could increase computational complexity when working on large graphs. Several models [39,120] adopted *k*-nearest methods to search neighbor nodes which can capture more graph structure. The *k*-nearest methods, therefore, could bring the advantage of reducing computational complexity since the models only take *k* neighbors as inputs. For example, Han et al. [120] proposed the similarity $S_{ij}$ between two nodes $v_{i}$ and $v_{j}$ as:

(8) $$S_{ij}=\left\{\begin{array}{cc} \exp\left(-\frac{\left|\right|v_{i}-v_{j}|{|}^{2}}{\delta^{2}}\right), & \mathrm{if}\;v_{j}\in N_{k}\left(v_{i}\right).\\ 0, & \mathrm{otherwise}.\; \end{array}\right.,$$

where $N_{k}\left(v_{i}\right)$ depicts the set of *k* nearest neighbors of $v_{i}$ in graphs. The model could measure the infringement of the constraints between pairs of nodes regarding label distribution. In addition, the model can estimate the correlation between features which would be beneficial to combine common features during the training process.

Several models [7,40,120,121,122] have been proposed to capture side information in graphs such as attributes and labels. He et al. [42] used the locality-preserving projection technique, a nonlinear Laplacian Eigenmap, to preserve the local structural information in graphs. The model first constructs an adjacency matrix with *k* nearest neighbors for each pair of nodes. The model then computes the objective function as:

(9) $$\begin{array}{c} a^{*}=\underset{a}{\arg\min}\;a^{⊺}XLX^{⊺}a\; \end{array}$$

(10) $$\begin{array}{c} \mathrm{subject}\;\mathrm{to}:\;a^{⊺}XLX^{⊺}a=1\; \end{array}$$

where *D* is a diagonal matrix, L=D−A is the Laplacian matrix, and *a* is the transformation matrix in the linear embedding $x_{i}\to y_{i}=A^{⊺}x_{i}$. Nevertheless, the idea from [42] only captures the structure within *k* nearest neighbors, which fails to capture the global similarity between nodes in the graph. Motivated by these limitations, Cao et al. [15] introduced the GraRep model, which considers a *k*-hop neighborhood of each target node. Accordingly, GraRep could capture global structural information in graphs. The model works with *k*-order probability transition matrix (proximity matrix) $M^{k}$ which could be defined as:

(11) $$M^{k}=\underset{k}{\underbrace{M\cdots M}}\;$$

where $M=D^{-1}A$, *D* is the degree matrix, *A* is the adjacent matrix, and $M_{ij}^{k}$ presents the transition probability from node $v_{i}$ to $v_{j}$. The loss function, thus, is the sum of *k* transition loss functions:

(12) $$\begin{array}{cc} L_{k}\left(v_{i}\right) & =\sum_{v_{j}\in N\left(v_{i}\right)}M_{ij}^{k}\log\sigma \left(Z_{i}^{⊺}Z_{j}\right)-\left|N_{neg}\right|\sum_{v_{m}∼P_{n}\left(v\right)}M_{im}^{k}\log\sigma \left(-Z_{i}^{⊺}Z_{m}\right)\;. \end{array}$$

To construct the vector embeddings, GraRep decomposed the transition matrix into small-sized matrices using SVD matrix factorization. Similarly, Li [123] introduced NECS (Learning network embedding with the community) to capture the high-order proximity using Equation (11).

**Table 3:** A summary of matrix factorization-based models for static graphs. *C* indicates the number of clusters in graphs, $N\left(Z_{i}|\mu_{c},\Sigma_{c}\right)$ refers to the multivariate Gaussian distribution for each cluster, *L* means the Laplacian matrix, $H\in R^{n\times k}$ is the probability matrix that a node belongs to a cluster, *U* denotes the coefficient vector, and $W_{ij}$ is the weight on $\left(v_{i},v_{j}\right)$.

Models	Graph Types	Tasks	Loss Function
SLE [39]	Static graphs	Node classification	$\sum_{v_{i}\in V}\sum_{v_{j}\in V}\left|W_{ij}\right|{∥Z_{i}-s_{ij}Z_{j}∥}_{2}^{2}$
[120]	Attributed graphs	Node classification	$\underset{W}{\arg\min}\;Tr\left(U^{⊺}X^{⊺}LXU\right)+\alpha_{}\sum_{\begin{array}{c} v_{i}\in V\\ v_{j}\in N\left(v_{i}\right) \end{array}}{∥Z_{i}-Z_{j}∥}_{2}^{2}+\alpha_{1}L_{1}+\alpha_{2}L_{2}$
[7]	Attributed graphs	Community detection	$-\sum_{(v_{i},v_{j})\in E}\log\sigma \left(Z_{i}^{⊺}Z_{j}\right)-\sum_{\begin{array}{c} v_{i}\in V\\ v_{j}\in N\left(v_{i}\right) \end{array}}\log\sigma \left(Z_{i}^{⊺}Z_{j}\right)$ $-\left|N_{neg}\right|\sum_{v_{k}∼P_{n}\left(v\right)}\log\sigma \left(-Z_{i}^{⊺}Z_{k}\right)-\sum_{v_{i}\in V,c=1}^{C}N\left(Z_{i}|\mu_{c},\Sigma_{c}\right)$
LPP [42]	Attributed graphs	Node classification	$\frac{1}{\left|V\right|}\sum_{v_{i}\in V}{∥y_{i}-{\hat{y}}_{i}∥}_{2}^{2}$
[121]	Attributed graphs	Graph reconstruction	$\frac{1}{\left|V\right|}\sum_{v_{i}\in V}{∥y_{i}-{\hat{y}}_{i}∥}_{2}^{2}$
[40]	Static graphs	Node clustering	$\sum_{v_{i}\in V}\sum_{c=1}^{C}∥Z_{i}-\mu_{c}∥$
GLEE [122]	Attributed graphs	Graph reconstruction, Link prediction	${∥L-\hat{L}∥}^{2}$
LPP [42]	Static graphs	Node classification	$\sum_{(v_{i},v_{j})\in E}{∥Z_{i}-Z_{j}∥}_{2}^{2}$
Grarep [15]	Static graphs	Node classification, Node clustering	$-\sum_{v_{i}\in V}\sum_{v_{j}\in N\left(v_{i}\right)}A_{ij}^{l}\log\sigma \left(Z_{i}^{⊺}Z_{j}\right)$ $-|N_{neg}|\sum_{v_{k}\sim P_{n}\left(v\right)}A_{ik}^{l}\log\sigma \left(\sum Z_{i}^{⊺}Z_{k}\right)$
NECS [123]	Static graphs	Graph reconstruction, Link prediction, Node classification	${∥M-\hat{M}∥}_{F}^{2}+\alpha_{1}{∥H-\hat{H}∥}_{F}^{2}+\alpha_{2}{∥H^{⊺}\hat{H}-I∥}_{F}^{2}$
HOPE [5]	Static graphs	Graph reconstruction Link prediction, Node classification	${∥M-Z\cdot Z^{⊺}∥}_{F}^{2}$
[124]	Static graphs	Link prediction	$\sum_{(v_{i},v_{j})\in S}A_{ij}{∥Z_{i}-Z_{j}∥}_{2}^{2}$
AROPE [86]	Static graphs	Graph reconstruction, Link prediction, Node classification	${∥M-Z\cdot Z^{⊺}∥}_{F}^{2}$
ProNE [43]	Static graphs	Node classification	$-\sum_{v_{i}\in V}\left[\sum_{v_{j}\in N\left(v_{i}\right)}\log\sigma \left(Z_{i}^{⊺}Z_{j}\right)+\sum_{v_{k}∼P_{n}\left(v\right)}\log\sigma \left(-Z_{i}^{⊺}Z_{k}\right)\right]$
ATP [6]	Static graphs	Link prediction	${∥M-Z\cdot Z^{⊺}∥}_{F}^{2}$
[125]	Static graphs	Graph partition	${\sum_{\begin{array}{c} (v_{i},v_{j})\in E\\ v_{i},v_{j}\in V_{k} \end{array}}\left(W_{ij}-\langle Z_{i}^{\left(k\right)},Z_{j}^{\left(k\right)}\rangle \right)}^{2}+\sum_{v_{i}\in V_{k}}{∥Z_{i}-{\hat{Z}}_{i}∥}_{2}^{2}$
NRL-MF [126]	Static graphs	Node classification	$\sum_{\begin{array}{c} v_{i}\in V\\ v_{j}\in N\left(v_{i}\right) \end{array}}{∥Z_{i}-Z_{j}∥}_{2}^{2}$

In terms of considering the node proximity based on neighbor relations, Ou et al. [5] presented HOPE, an approach for preserving structural information in graphs using *k*-order proximity. In contrast to GraRep, HOPE tried to solve the asymmetric transitivity problem in directed graphs by approximating high-order proximity. The objective function needs to be minimized for the approximation proximity could be defined as:

(13) $$Z^{*}=\underset{Z}{\arg\min}\;{∥M_{ij}-Z_{i}^{⊺}Z_{j}∥}_{2}^{2}\;$$

where *M* is the high-order proximity matrix, for instance, $M_{ij}$ presents the proximity of two nodes $v_{i}$ and $v_{j}$, $Z_{i}$ and $Z_{j}$ denote vector embeddings of $v_{i}$ and $v_{j}$, respectively. The proximity matrix *M* can be measured by decomposing into two small-sized matrices $\left(M=M_{g}^{-1}\cdot M_{l}\right)$. Several common criteria could measure the node proximity, such as Katz Index [127], Rooted PageRank [128], Adamic-Adar [129], and Common Neighbors. Coskun and Mustafa [124] suggested changes in the proximity measure formulas of the HOPE model. For nodes that have a small degree, singular values could be zero after measuring the node proximity. Therefore, to solve this problem, they added a parameter σ to regularize the Laplacian graph.

A few models have been proposed with the same idea as HOPE and GraRep [43,86]. For example, ProNE model [43] aimed to use *k* number of the Chebyshev expansion to avoid Eigen decomposition, instead of using *k*-order proximity in HOPE models. Sun et al. [6] introduced a similar approach for preserving asymmetric transitivity with high-order proximity. However, the significant difference is that they proposed a strategy to break directed acyclic graphs while preserving the graph structure. The non-negative matrix factorization could then be applied to produce an embedding matrix. Several models [125,130,131] mainly focused on the pointwise mutual information (PMI) of nodes in graphs which calculates the connection between nodes in terms of linear and nonlinear independence. Equation (5) is used to learn node embeddings.

Several models aimed to reduce computational complexity from matrix factorization by improving the sampling strategies [126,132,133]. For instance, the key idea of the NRL-MF model [126] was to deal with a hashing function for computing dot products. Each node is presented as a binarized vector by a hashing function, which can be calculated faster by XOR operators. The model could learn the binary and quantized codes based on matrix factorization and preserve high-order proximity. Jiezhong [133] targeted sparse matrix factorization. They implemented random-walk sampling on graphs to construct a NetMF Matrix Sparsifier. RNP model [132] explored in-depth vector embeddings based on personalized PageRank values, then approximated the PPR matrices.

**Table 4:** A summary of matrix factorization-based models for heterogeneous graphs and dynamic graphs. $H\in R^{n\times k}$ is the probability matrix that a node belongs to a cluster, $E^{\left(t\right)}$ is the edge matrix with type *t*, $W_{ij}$ is the weight on $\left(v_{i},v_{j}\right)$, *r* denotes the relation type, and $E^{\left(1,2\right)}$ is the set of edges in two component graphs $G_{1}$ and $G_{2}$.

Models	Graph Types	Tasks	Loss Function
DBMM [134]	Dynamic graphs	Node classification, Node clustering	${∥A-\hat{A}∥}_{F}^{2}$
[135]	Dynamic graphs	Link prediction	${∥A-\hat{A}∥}_{2}^{2}+\alpha_{}L_{2}$
[136]	Dynamic graphs	Link prediction	${∥A-\hat{A}∥}_{F}^{2}$
LIST [137]	Dynamic graphs	Link prediction	${∥A-\hat{A}∥}_{F}^{2}+L_{2}$
TADW [131]	Attributed graphs	Node classification	${∥M-W^{⊺}HX∥}_{F}^{2}+\alpha \left({∥W∥}_{F}^{2}+{∥H∥}_{F}^{2}\right)$
PME [138]	Heterogeneous graphs	Link prediction	$\sum_{\begin{array}{c} \left(v_{i},v_{j}\right)\in E^{\left(r\right)}\\ \left(v_{i},v_{k}\right)\notin E^{\left(r\right)} \end{array}}\left({∥Z_{i}-Z_{j}∥}_{2}^{2}-{∥Z_{i}-Z_{n_{k}}∥}_{2}^{2}+m\right)$
EOE [139]	Heterogeneous graphs	Node classification	$\sum_{\left(v_{i},v_{j}\right)\in E^{\left(1,2\right)}}\sigma \left(Z_{i}^{⊺}Z_{j}\right)-\sum_{\left(v_{l},v_{k}\right)\notin E^{\left(1,2\right)}}\sigma \left(-Z_{i}^{⊺}Z_{j}\right)+\alpha_{1}L_{1}+\alpha_{2}L_{2}$
[130]	Heterogeneous graphs	Link prediction	$\sum_{\left(v_{i},v_{j}\right)\in E^{\left(t\right)}}{∥Z_{i}^{\left(t\right)}-{\hat{Z}}_{i}^{\left(t\right)}∥}_{F}^{2}+\alpha_{1}L_{1}+\alpha_{2}L_{2}$
ASPEM [140]	Heterogeneous graphs	Node classification, Link prediction	$-\sum_{\begin{array}{c} v_{i}\in V\\ (v_{i},v_{j},r)\in E \end{array}}\log\left(p(v_{i}|v_{j},r)\right)$
MELL [141]	Heterogeneous graphs	Link prediction	$-\sum_{(v_{i},v_{j})\in E}\sigma \left(Z_{i}^{⊺}Z_{j}\right)-\sum_{(v_{i},v_{k})\notin E}\sigma \left(1-Z_{i}^{⊺}Z_{k}\right)+\alpha L_{2}$
PLE [142]	Attributed graphs	Node classification	$\sum_{(v_{i},v_{j})\in E}\log\sigma \left(Z_{i}Z_{j}\right)+\left|N_{neg}\right|E_{v_{k}∼P_{n}\left(v_{k}\right)}\left(\log\sigma (-Z_{i}Z_{k})\right)$

In the real world, several graphs often contain attributes for nodes and edges, such as user profiles on a social network. These attributes provide helpful information to improve the node representation and help to learn node embedding. Yang et al. [131] proposed the TADW model by representing the DeepWalk model as a matrix factorization and integrating text features into the factorization model. Ren et al. [142] introduced the PLE model to learn jointly different types of nodes and edges with text attributes. Since existing models often ignore the noise of labels, PLE is the first work to investigate the noisy type labels by measuring the similarity between entities and type labels.

Beyond static and homogeneous graphs, several models have been proposed to learn embeddings in dynamic and heterogeneous graphs. The embedding models for dynamic graphs are essentially the same as for static graphs, including Laplacian eigenmaps methods and node proximity matrix factorization to model relations in dynamic graphs over time. For Laplacian eigenmaps methods, Li et al. [81] presented DANE (Dynamic Attributed Network Embedding) model to learn node embeddings in dynamic graphs. The main idea of the DANE model is to represent a Laplacian matrix as $L_{A}^{\left(t\right)}=D_{A}^{\left(t\right)}-A^{\left(t\right)}$, where $A^{\left(t\right)}\in R^{n\times n}$ is the adjacency matrix of dynamic graphs at time *t*, $D_{A}$ is the diagonal matrix, then the model could be able to learn node embeddings by time in an online manner. To preserve the node proximity, the DANE model aimed to minimize the loss function:

(14) $$\begin{array}{c} L(v_{i},v_{j})=\sum_{\begin{array}{c} \left(v_{i},v_{j}\right)\\ i\neq j \end{array}}A_{ij}^{\left(t\right)}{∥Z_{i}-Z_{j}∥}_{2}^{2}\;. \end{array}$$

The eigenvectors λ of the Laplacian matrix *L* can be calculated by solving the generalized eigenproblem: $L_{A}^{\left(t\right)}a=\lambda D_{A}^{\left(t\right)}$, where $a=\langle a_{0}\;a_{1}\;\cdots \;a_{N}\rangle$ is the eigenvectors.

Several models applied node proximity matrix factorization directly to dynamic graphs by updating the proximity matrix between entities in the dynamic graphs. Rossi et al. [134] presented dynamic graphs as a set of static graph snapshots: $G=\{G\left(t_{0}\right)\;G\left(t_{1}\right)\;\cdots \;G\left(t_{N}\right)\}$. The model then learned a transition proximity matrix *T*, which describes all transitions from the dynamic graphs. For evaluation, they predict the graph *G* at time t+1: ${\hat{G}}_{t+1}=G_{t}T_{t+1}$, then estimate the error using Frobenius loss: ${∥{\hat{G}}_{t+1}-G_{t+1}∥}_{F}$. Zhu et al. [135,137] aimed to preserve the graph structure based on temporal matrix factorization during the network evolution. Given an adjacency matrix A(t) at time *t*, two temporal rank-*k* matrix factorization *U* and V(t) are factorized as $A\left(t\right)=f\left(UV{\left(t\right)}^{⊺}\right)$, and the objective is to minimize the loss function $L\left(A\right)$ which could be defined as:

(15) $$\begin{array}{c} L\left(A\right)=\sum_{t=1}^{T}\frac{D(t)}{2}{∥A\left(t\right)-\hat{A}\left(t\right)∥}_{F}^{2}\;. \end{array}$$

Matrix factorization models have been successfully applied to graph embedding, mainly for the node embedding problem. Most models are based on singular value decomposition to find eigenvectors in the latent space. There are several advantages of matrix factorization-based models:

- Training data requirement: The matrix factorization-based models do not need much data to learn embeddings. Compared to other methods, such as neural network-based models, these models bring advantages in case there is little training data.
- Coverage: Since the graphs are presented as Laplacian matrix *L*, or transition matrix *M*, then the models could capture all the proximity of the nodes in the graphs. The connection of all the pairs of nodes is observed at least once time under the matrix that makes the models could be able to handle sparsity graphs.

Although matrix factorization is widely used in graph embedding problems, it still has several limitations:

- Computational complexity: The matrix factorization suffers from time complexity and memory complexity for large graphs with millions of nodes. The main reason is the time it takes to decompose the matrix into a product of small-sized matrices [15].
- Missing values: Models based on matrix factorization cannot handle incomplete graphs with unseen and missing values [143,144]. When the graph data are insufficient, the matrix factorization-based models could not learn generalized vector embeddings. Therefore, we need neural network models that can generalize graphs and better predict entities in graphs.

### 3.3. Shallow Models

This section focuses on shallow models for mapping graph entities into vector space. These models mainly aim to map nodes, edges, and subgraphs as low-dimensional vectors while preserving the graph structure and entity proximity. Typically, the models first implement a sampling technique to capture graph structure and proximity relation and then learn embeddings based on shallow neural network algorithms. Several sampling strategies could be taken to capture the local and global information in graphs [14,145,146]. Based on the sampling strategy, we divide shallow models into two main groups: structure preservation and proximity reconstruction.

**Figure 6:**
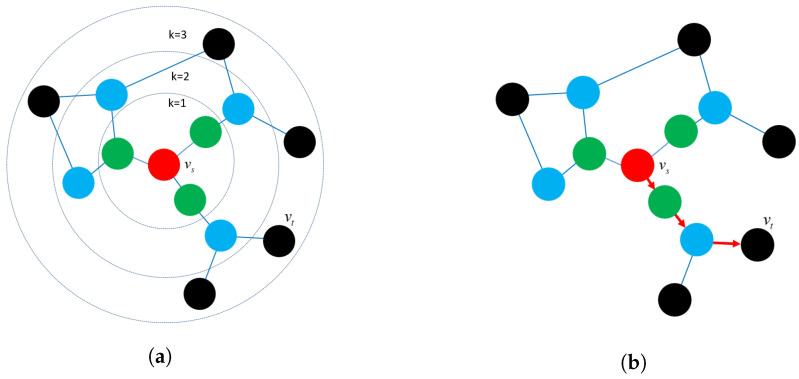
Node sampling techniques. (**a**) *k*-hop sampling; (**b**) Random-walk sampling. The source node $v_{s}$ and the target node $v_{t}$ are taken as the source node and the target node in the graph. In (**a**), the *k*-hop proximity sampling strategy begins from source node $v_{s}$, and the green nodes are considered to be the 1st-hop proximity of node $v_{s}$. The blue and the black nodes are considered 2nd-hop and 3rd-hop proximity of node $v_{s}$, respectively. In (**b**), the random-walk sampling strategy takes a random walk (red arrow) from the source node $v_{s}$ to the target node $v_{t}$.

- Structure preservation: The primary concept of these approaches is to define sampling strategies that could capture the graph structure within fixed-length samples. Several sampling techniques could capture both local and global graph structures, such as random-walk sampling, role-based sampling, and edge reconstruction. The model then applies shallow neural network algorithms to learn vector embeddings in the latent space in an unsupervised learning manner. Figure 6a shows an example of a random-walk-based sampling technique in a graph from a source node $v_{s}$ to a target node $v_{t}$.
- Proximity reconstruction: It refers to preserving a *k*-hop relationship between nodes in graphs. The relation between neighboring nodes in the *k*-hop distance should be preserved in the latent space. For instance, Figure 6b presents a 3-hop proximity from the source node $v_{s}$.

In general, shallow models have achieved many successes in the past decade [4,14,21]. However, there are several disadvantages of shallow models:

- Unseen nodes: When there is a new node in graphs, the shallow models cannot learn embeddings for new nodes. To obtain embedding for new nodes, the models must update new patterns, for example, re-execute random-walk sampling to generate new paths for new nodes, and then the models must be re-trained to learn embeddings. The re-sampling and re-training procedures make it impractical to apply them in practice.
- Node features: Shallow models such as DeepWalk and Node2Vec mainly work suitably on homogeneous graphs and ignore information about the attributes/labels of nodes. However, in the real world, many graphs have attributes and labels that could be informative for graph representation learning. Only a few studies have investigated the attributes and labels of nodes, and edges. However, the limitations of domain knowledge when working with heterogeneous and dynamic graphs have made the model inefficient and increased the computational complexity.
- Parameter sharing: One of the problems of shallow models is that these models cannot share the parameters during the training process. From the statistical perspective, parameter sharing could reduce the computational time and the number of weight updates during the training process.

#### 3.3.1. Structure-Preservation Models

Choosing a strategy to capture the graph structure is essential for shallow models to learn vector embeddings. The graph structure can be sampled through connections between nodes in graphs or substructures (e.g., subgraphs, motifs, graphlets, roles, etc.). Table 5 briefly summarizes structure-preservation models for static and homogeneous graphs.

Over the last decade, various models have been proposed to capture the graph structure and learn embeddings [4,21,147,148]. Among those models, random-walk-based strategies could be considered one of the most typical strategies to sample the graph structures [4,14]. The main idea of the random-walk strategy is to gather information about the graph structure to generate paths that can be treated as sentences in documents. The definition of random walks could be defined as:

**Definition** **6** (Random walk [14]). *Given a graph G=(V,E), where V is the set of nodes and E is the set of edges, a random walk with length l is a process starting at a node $v_{i}\in V$ and moving to its neighbors for each time step. The next steps are repeated until the length l is reached.*

Two models, DeepWalk [14] and Node2Vec [4] could be considered to be pioneer models to open a new direction for learning node embeddings.

Inspired by the disadvantages of the matrix factorization-based models, the DeepWalk model could preserve the node neighborhoods based on random-walk sampling, which could capture global information in graphs. Moreover, both DeeWalk and Node2Vec aim to maximize the probability of observing node neighbors by stochastic gradient descent on each single-layer neural network. Therefore, these models reduce running time and computational complexity. DeepWalk [14] is a simple node embedding model using the random-walk sampling strategy to generate node sequences and treat them as word sentences. The objective of DeepWalk is to maximize the probability of the set of neighbor nodes $N(v_{i})$ given a target node $v_{i}$. Formally, the optimization problem could be defined as:

(16) $$\phi^{*}\left(\cdot \right)=\arg\min_{\phi (\cdot )}-\log p\left(N\left(v_{i}\right)|\phi (v_{i})\right)\;$$

where $v_{i}$ denotes the target node, $N(v_{i})$ is the set of neighbors of $v_{i}$ which could be generated from random-walk sampling, $\phi (v_{i})$ is the mapping function $\phi :v_{i}\in V\to R^{|V|\times d}$. The model uses two strategies for finding neighbors given a source node, based on the Breadth-First Search (BFS) and Depth First Search (DFS) strategies. The BFS strategy aims to represent a microscopic view that captures the local structure. In contrast, the DFS strategy delivers the global structure information in graphs. The DeepWalk then uses a skip-gram model and stochastic gradient descent (SGD) to learn latent representations.

**Table 5:** A summary of structure-preservation models for homogeneous and static graphs. *K* indicates the number of clusters in the graph, and $\mu_{k}$ refers to the mean value of cluster *k*.

Models	Graph Types	Tasks	Loss Function
DeepWalk [14]	Static graphs	Node classification	$-\sum_{v_{i}\in V}y_{i}^{⊺}\log\left({\hat{y}}_{i}\right)$
Node2Vec [4]	Static graphs	Node classification, Link prediction	$-\sum_{v_{i}\in V}y_{i}^{⊺}\log\left({\hat{y}}_{i}\right)$
WalkLets [147]	Static graphs	Node classification	$-\sum_{v_{i}\in V}y_{i}^{⊺}\log\left({\hat{y}}_{i}\right)$
Div2Vec [149]	Static graphs	Link prediction	$-\sum_{v_{i}\in V}y_{i}^{⊺}\log\left({\hat{y}}_{i}\right)$
	Static graphs	Node classification	$-\sum_{v_{i}\in V}y_{i}^{⊺}\log\left({\hat{y}}_{i}\right)$
Node2Vec+ [148]	Static graphs	Node classification	$-\sum_{v_{i}\in V}y_{i}^{⊺}\log\left({\hat{y}}_{i}\right)$
Struct2Vec [21]	Static graphs	Node classification	$-\sum_{v_{i}\in V,\langle v_{i},v_{j}\rangle \in E}\log\left(\sigma (Z_{i}^{⊺}Z_{j})\right)-\left|N_{neg}\right|\sum_{v_{k}∼P_{n}\left(v\right)}\log\left(\sigma (-Z_{i}^{⊺}Z_{k})\right)$
DiaRW [150]	Static graphs	Node classification, Link prediction	$\sum_{v_{i}\in V}-y_{i}^{⊺}\log\left({\hat{y}}_{i}\right)-\left(1-y_{i}\right)\log\left(1-{\hat{y}}_{i}\right)$
Role2Vec [151]	Attributed graphs	Link prediction	$\sum_{k=1}^{K}\sum_{v_{i}\in V_{k}}{∥Z_{i}-\mu_{k}∥}_{2}^{2}$
NERD [152]	Directed graphs	Link Prediction, Graph Reconstruction, Node classification	$\sum_{(v_{i},v_{j})\in E}\log\sigma \left(Z_{i}Z_{j}\right)+\left|N_{neg}\right|E_{v_{k}∼P_{n}\left(v_{k}\right)}\left(\log\sigma (-Z_{i}Z_{k})\right)$
Sub2Vec [153]	Static graphs	Community detection, graph classification	$\sum_{k=1}^{K}\sum_{v_{i}\in V_{k}}{∥Z_{i}-\mu_{k}∥}_{2}^{2}$
Subgraph2Vec [145]	Static graphs	Graph classification, Clustering	$-\sum_{v_{i}\in V}y_{i}^{⊺}\log\left({\hat{y}}_{i}\right)$
RUM [89]	Static graphs	Node classification, Graph reconstruction	$-\sum_{v_{i}\in V}y_{i}^{⊺}\log\left({\hat{y}}_{i}\right)$
Gat2Vec [154]	Attributed graphs	Node classification, Link prediction	$-\sum_{v_{i}\in V,\langle v_{i},v_{j}\rangle \in E}\log\left(\sigma (Z_{i}^{⊺}Z_{j})\right)-\left|N_{neg}\right|\sum_{v_{k}∼P_{n}\left(v\right)}\log\left(\sigma (-Z_{i}^{⊺}Z_{k})\right)$
ANRLBRW [155]	Attributed graphs	Node classification	$-\sum_{v_{i}\in V,\langle v_{i},v_{j}\rangle \in E}\log\left(\sigma (Z_{i}^{⊺}Z_{j})\right)-\left|N_{neg}\right|\sum_{v_{k}∼P_{n}\left(v\right)}\log\left(\sigma (-Z_{i}^{⊺}Z_{k})\right)$
Gl2Vec [88]	Static graphs	Node classification	$-\sum_{v_{i}\in V}y_{i}^{⊺}\log\left({\hat{y}}_{i}\right)$

**Figure 7:**
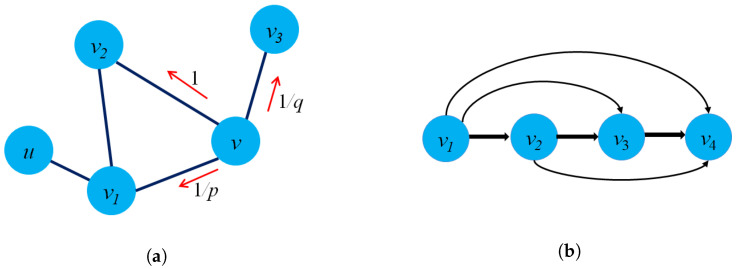
Sampling strategy in Node2Vec and WalkLets model. (**a**) Sampling strategy in Node2Vec model; (**b**) Sampling strategy in WalkLets model. In (**a**), assume a random path from the DeepWalk model is of the form: $\left(v_{1}\to v_{2}\to v_{3}\to v_{4}\right)$, then the corpus of random walk pairs at scale k=3 is: $A_{1}=\left\{\left(v_{1},v_{2}\right),\left(v_{2},v_{3}\right),\left(v_{3},v_{4}\right)\right\}$, $A_{2}=\left\{\left(v_{1},v_{3}\right),\left(v_{2},v_{4}\right)\right\}$, and $A_{3}=\left\{\left(v_{1},v_{4}\right)\right\}$. In (**b**), there are two parameters: The return parameter *p* and the in–out parameter *q*. Parameters 1,1/p, and 1/q are conditional probabilities. Starting at node *u* and now at *v*, the random walk looks at the next node based on the probabilities 1/p and 1/q.

One of the limitations of DeepWalk is that the model can only capture the graph structure but fail to navigate the random-walk sampling to enrich the quality of the sampling graph structure. To overcome the limitations of DeepWalk, Grover and Leskovec introduced Node2Vec [4] with a flexible random-walk sampling strategy to navigate random walks via each time step. The key difference between DeepWalk and Node2Vec is that instead of using a truncated random walk, the model used a biased random-walk sampling process with two parameters (*p* and *q*) to adjust the random walk on graphs. Figure 7a presents two parameters *p* and *q* in Node2Vec model in detail. The model could capture more information on the graph structure locally and globally by introducing constraints when deciding the subsequent nodes visited.

Perozzi et al. [147] presented the WalkLets model, which was extended from the DeepWalk model. They modified the random-walk sampling strategy to capture more graph structure information by skipping and passing over multiple nodes at each time step. Therefore, these sampling strategies can capture more global graph structure by the power of the transition matrix when passing over multiple nodes. The main idea of the WalkLets model is to represent the random-walk paths as pairs of nodes in the multi-scale direction. Figure 7b depicts the sampling strategy of the WalkLets model using multi-scale random-walk paths. However, one of the limitations of the WalkLets is that the model could not distinguish local and global structures when passing and skipping over nodes in graphs. Jisu et al. [149] presented a variation of DeepWalk, named Div2Vec model. The main difference between the two models is the way that Div2Vec chooses the next node in the random-walk path, which will be visited based on the degree of neighboring nodes. The focus on the degree of neighboring nodes could help the models learn the importance of nodes that are popular in social networks. Therefore, at the current node $v_{i}$, the probability of choosing the next node $v_{j}$ in a random-walk path is calculated as:

(17) $$p\left(v_{j}|v_{i}\right)=\frac{f(\deg\left(v_{j}\right)}{\sum_{v_{i}\in N}f(\deg\left(v_{i}\right))}\;$$

where $\deg(v_{j})$ depicts the degree of node $v_{j}$, and $f\left(\deg\left(v_{j}\right)\right)=\frac{1}{\deg(v_{j})}$. Renming et al. [148] presented Node2Vec+, an improved version of Node2Vec. One limitation of the Node2Vec model is that it cannot determine the following nodes based on the target nodes. There is a significant difference between Node2Vec and Node2Vec+. The Node2Vec+ model can determine the state of the potential edge for a given node, therefore enhancing the navigability of the Node2Vec model to capture more graph structure. In particular, they introduced three neighboring edge states from a current node (out edge, noisy edge, and in edge) which are calculated to decide the next step. With potential out edges $(v_{i},v_{j})\in E$ from previous node *t*, the in–out parameters *p* and *q* of Node2Vec model could then be re-defined as bias factor α as:

(18) $$\alpha_{pq}\left(t,v_{i},v_{j}\right)=\left\{\begin{array}{cc} \frac{1}{p}\; & \mathrm{if}\;t=x.\\ 1\; & \mathrm{if}\;w\left(v_{j},t\right)\geq \tilde{d}\left(v_{j}\right).\\ \min\{1,\frac{1}{p}\}\; & \mathrm{if}\;w\left(v_{j},t\right)<\tilde{d}\left(v_{j}\right)\;\mathrm{and}\;w\left(v_{j},t\right)<\tilde{d}\left(v_{i}\right).\\ \frac{1}{q}+\left(1-\frac{1}{q}\right)\frac{w(v_{j},t)}{d(x)}\; & \mathrm{if}\;w\left(v_{j},t\right)<\tilde{d}\left(v_{j}\right)\;\mathrm{and}\;w\left(v_{j},t\right)\geq \tilde{d}\left(v_{i}\right). \end{array}\right.\;$$

where $\tilde{d}\left(v_{i}\right)$ denotes a noisy edge threshold which could consider the next node state $v_{j}$ from the current node *t* and could be viewed as the weights of edges, $w(v_{i},v_{j})$ is the weight of the edge between $v_{i}$ and $v_{j}$.

In contrast to preserving graph topology which mainly focuses on distance relations, several models aimed to preserve the role and importance of nodes in graphs. In the case of social networks, for example, we could discover influencers with the ability to impact several activities of communities. In contrast to the random-walk-based technique, several studies [21,150] used the term “role-based” to preserve the nodes’ role, which random-walk-based sampling strategies cannot capture in a fixed length. Therefore, by preserving the role of nodes, role-based models could capture the structural equivalent. Ribeiro et al. [21] introduced the Struc2Vec model to capture graph structure based on the nodes’ role. Nodes that have the same degree should be encoded close in the vector space. Given a graph *G*, they introduced *k* graphs, each graph can be considered in one layer. Each layer denotes a graph that describes the weighted node degree from different hop distances. Specifically, at layer $L_{k}$, for each node $v_{i}\in V$, there are three probabilities of going to node $v_{j}$ in the same layer, jumping to the previous layer $L_{k-1}$ and next layer $L_{k+1}$:

(19) $$\begin{array}{cc} p_{k}\left(v_{i}^{k},v_{j}^{k}\right) & =\frac{e^{-f_{k}\left(v_{i}^{k},v_{j}^{k}\right)}}{Z_{k}\left(v_{i}^{k}\right)}\; \end{array}$$

(20) $$\begin{array}{cc} p_{k}\left(v_{i}^{k},v_{i}^{k+1}\right) & =\frac{w(v_{i}^{k},v_{i}^{k+1})}{w\left(v_{i}^{k},v_{i}^{k+1}\right)+w\left(v_{i}^{k},v_{i}^{k-1}\right)}\; \end{array}$$

(21) $$\begin{array}{cc} p_{k}\left(v_{i}^{k},v_{i}^{k-1}\right) & =1-p_{k}\left(v_{i}^{k},v_{i}^{k+1}\right)\; \end{array}$$

where $f_{k}\left(v_{i}\;v_{j}\right)$ presents the role-based distance between nodes $v_{i}$ and $v_{j}$, and w(·) denotes the edge weight. Zhang et al. [150] presented the DiaRW model, which uses a random-walk strategy based on the node degree. The difference between other role-based models and the DiaRW model is that they used random walks that can vary in length based on the node degree. One of the limitations of the Struc2Vec model is that the model could not preserve the similarity of nodes in graphs. Motivated by this limitation, the DiaRW model aims to capture structural identity based on node degree and the neighborhood in which nodes have a high degree. The purpose of this model is to collect structural information around higher-order nodes, which is a limitation of models based on fixed-length random walks. Ahmed et al. [151] introduced the Role2Vec model that could capture the node’s similarity and structure by introducing a node-type parameter to guide random-walk paths. The core idea of Role2Vec is that nodes in the same cluster should be sampled together in the random-walk path. By only sampling nodes in the same clusters, Role2Vec could learn correct patterns with reduced computational complexity. The model then uses the skip-gram model to learn node embeddings. Unlike Rol2Vec, the NERD model [152] considers nodes’ asymmetric roles for directed graphs. The model sampled the neighbor’s nodes using an alternative random walk. The probability of the next node $v_{i+1}$ from the current node $v_{i}$ in the random-walk path could be defined as:

(22) $$p\left(v_{i+1}|v_{i}\right)=\left\{\begin{array}{cc} \frac{1}{d^{out}\left(v_{i}\right)}\cdot w\left(v_{i},v_{j}\right)\;\; & \mathrm{if}\;(v_{i},v_{j})\in E\;.\\ \frac{1}{d^{in}\left(v_{i}\right)}\cdot w\left(v_{i},v_{j}\right)\; & \mathrm{if}\;(v_{j},v_{i})\notin E\;.\\ 0\; & \mathrm{otherwise}\;. \end{array}\right.\;$$

where $w(v_{i},v_{j})$ is the weight of the edge $e_{ij}$   $d^{in}\left(v_{i}\right)$ and $d^{out}\left(v_{i}\right)$ present the total in-degree and out-degree of the node $v_{i}$, respectively.

In some types of graphs, nodes in the same subgraphs tend to have similar labels. Studying low-level node representation could not bring significant generalization. Instead of embedding individual nodes in graphs, several studies aimed to learn subgraph similarity or the whole graphs. Inspired by representations of sentences and documents in the NLP (natural language processing) area, Bijaya et al. [153] proposed the Sub2Vec model to embed each subgraph into a vector embedding.

**Figure 8:**
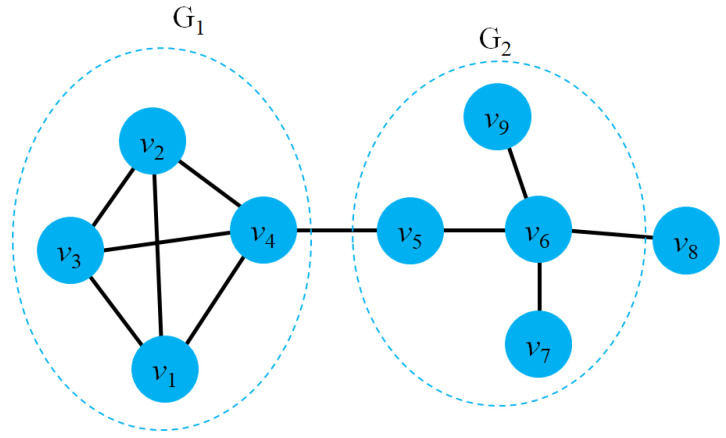
Sampling strategy in Sub2Vec model. Assume that there are two subgraphs $G_{1}=\left\{v_{1},v_{2},v_{3},v_{4}\right\}$, and $G_{2}=\left\{v_{5},v_{6},v_{7},v_{9}\right\}$. For neighborhood properties, the model uses random-walk sampling on all nodes in subgraphs $G_{1}$ and $G_{2}$ to capture the subgraph structure. For structural properties, they introduced a ratio of node degree when sampling. With the length of the random-walk path is 3, then the degree path for $G_{1}$ is 0.75→0.75→0.75, while the degree path from node $v_{5}$ to $v_{9}$ is: 0.25→0.75→0.25.

To learn a subgraph embedding $S=\{G_{1}\;G_{2}\;\cdots \;G_{n}\}$ from an original graph *G*, two properties should be preserved: similarity and structural property. The former ensures the connection between subgraph nodes by collecting sets of paths in a subgraph. The latter ensures that each node in a subgraph should be densely connected to all other nodes in the same subgraph. Figure 8 presents two subgraph properties that could capture each subgraph connection and structure.

In contrast to Sub2Vec, Subgraph2Vec [145] aimed to learn rooted subgraph embeddings for detecting Android malware. One of the advantages of this model with the Sub2Vec model is that Subgraph2Vec could consider different degrees of rooted subgraphs surrounding the target subgraph while Sub2Vec tried to detect the community. Annamalai et al. [156] targeted embedding the entire graph into the latent space. With the same idea as the Subgraph2Vec model, they extracted the set of subgraphs from the original graph using the WL relabeling strategy. However, the difference is that they used the Doc2Vec model by treating documents as graphs to learn graph embeddings.

Most models mentioned above aim to capture the graph structure based on low-level node representation, which could fail to represent the higher-level structure. Therefore, finding the community structure can be difficult for models based on random-walk sampling strategies. Motif-based models are one of the strategies to preserve the local structure and discover the global structure of graphs. Yanlei et al. [89] proposed the RUM (network Representation learning Using Motifs) model to learn small groups of nodes in graphs. The main idea of RUM was to build a new graph $G^{\prime }=\left(V^{\prime },E^{\prime }\right)$ based on the original graph by constructing new nodes and edges as follows:

- Generating nodes in graph $G^{\prime }$: Each new node *v* in graph $G^{\prime }$ is a tuple $v_{ijk}=\langle v_{i},v_{j},v_{k}\rangle$ in the original graph *G*. Therefore, they can map the triangle patterns of the original graph to the new graph for structure preservation.
- Generating edges of graph $G^{\prime }$: Each edge of the new graph is formed from two nodes that have two edges in common in the original graph. For example, the edge $e=(v_{ijk},v_{ijl})$ denotes that we the edge $(v_{i},v_{j})\in E$ in the original graph *G*.

**Figure 9:**
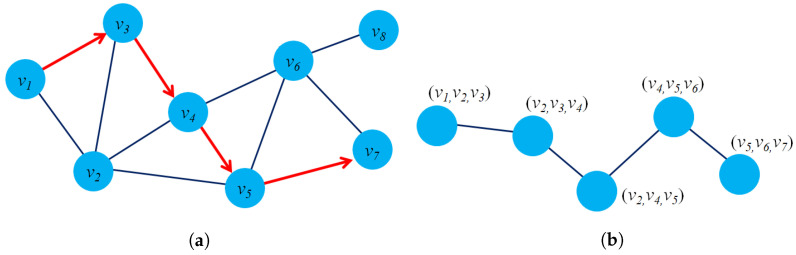
The random-walk sampling based on motif. (**a**) Random-walk sampling; (**b**) Motif-based random-walk sampling. (**a**) presents a random-walk path from node $v_{1}$ to $v_{7}$: $v_{1}\to v_{3}\to v_{4}\to v_{5}\to v_{7}$. In (**b**), the motif-based path is: $\langle v_{1},v_{2},v_{3}\rangle \to \langle v_{2},v_{3},v_{4}\rangle \to \langle v_{2},v_{4},v_{5}\rangle \to \langle v_{4},v_{5},v_{6}\rangle$.

The model then used the skip-gram model to learn the node and motif embeddings. Figure 9b depicts the details of the random-walk sampling strategy based on motifs.

There are also several models based on motifs for heterogeneous graphs [87,90,91]. For instance, Qian et al. [90] proposed the MBRep model (Motif-based representation) with the same idea from the RUM model to generate a hyper-network based on a triangle motif. However, the critical difference is that the MBRep model could extract motifs based on various node and edge types in heterogeneous graphs.

Most of the above models aim to learn node embeddings without side information, which could be informative for learning graph structure. However, graphs in the real world could be composed of side information, such as attributes of nodes and edges. Several models tried to learn node embeddings in attributed graphs by adding node properties presented as attributed graphs. Nasrullah et al. [154] proposed the Gat2Vec model to capture the contextual attributes of nodes. Given by a graph G=(V,E,X) where *X* is the attribute function $X:V\to 2^{X}$, they generated a structural graph $G_{s}$ and a bipartite graph $G_{a}$ as:

(23) $$\begin{array}{cc} \; & G_{s}=\left(V_{s}\;E\right)\; \end{array}$$

(24) $$\begin{array}{cc} \; & G_{a}=\left(V_{a}\;X\;E_{a}\right)\; \end{array}$$

where $V_{s}\subseteq V$, $V_{a}=\left\{v_{i}:X\left(v_{i}\right)\neq ⌀\right\}$, $V_{a}\subseteq V$, and $E_{a}=\left\{\left(v_{i},a\right),a:X\left(v_{i}\right)\right\}$. They then used the random-walk sampling strategy to capture the graph structure in both types of graphs. Similar to Gat2Vec, Wei et al. [155] introduced the ANRLBRW model (Attributed Network Representation Learning Based on Biased Random Walk) with the idea of splitting the original graph *G* into a geological graph and attributed graph. However, there is a slight difference between the two models. ANRLBRW model used a biased random-walk sampling inspired by Node2Vec, which includes two parameters *p* and *q* in the sampling strategy. Kun et al. [88] introduced the Gl2Vec model to learn node embeddings based on graphlets. To generate the feature representation for graphs, they capture the proportion of graphlet occurrences in a graph compared with random graphs.

For social networks, the connections of nodes are far more complex than the node-to-node edge relationship, which constructs hypergraphs. In contrast to homogeneous graphs, edges in hypergraphs could connect more than two nodes in graphs which leads to difficult learning node embeddings. Several models have been proposed to learn node and edge embeddings in the hypergraphs [157,158]. For example, Yang et al. [157] proposed the LBSN2Vec (Location Based Social Networks) model, a hypergraph embedding model to learn hyperedges including both user-user connection and user-check-in locations over time. Since most existing models fail to capture mobility features and co-location rates dynamically, the model could learn the impact of user mobility in social networks for prediction tasks. The objective of this model is to use a random-walk-based sampling strategy on hyperedges with a sequence length to capture the hypergraph structure. They then use cousin similarity to preserve nodes’ proximity in the random-walk sequences. Table 6 lists a summary of representative models for heterogeneous graphs.

Several types of graphs in the real world are heterogeneous, with different node and edge types. Most of the above models fail to capture heterogeneous graphs. Several models have been proposed to capture the heterogeneous graph structure [159,164,166]. Dong et al. [20] introduced the Metapath2Vec model, the idea based on random walks to learn node embeddings in heterogeneous graphs. One of the powers of meta-path is that it can capture the relationship between various types of nodes and edges in heterogeneous graphs. To capture the structure of heterogeneous graphs with different types of nodes and edges, they presented meta-path random walks *P* with length *l*:

(25) $$P:v_{1}\overset{t_{1}}{\to }v_{2}\overset{t_{2}}{\to }\cdots \overset{t_{k-1}}{\to }v_{k}\overset{t_{k}}{\to }\cdots \overset{t_{l-1}}{\to }v_{l}\;$$

where $t_{i}$ presents the relation type between nodes $v_{i}$ and $v_{i+1}$. Therefore, the transition probability of node $v_{i+1}$ given by node $v_{i}$ in the meta-path *P* could be defined as:

(26) $$p(v_{i+1}|v_{i}^{t},P)=\left\{\begin{array}{cc} \frac{1}{|N_{t+1}\left(v_{i}^{t}\right)|}\; & \mathrm{if}\;\left(v_{i+1},v_{i}^{t}\right)\in E\;E^{\left(t\right)}\left(v_{i+1}\right)=t+1\;.\\ 0, & \mathrm{otherwise}\;. \end{array}\right.,$$

where $N_{t+1}\left(v_{i}^{t}\right)$ is the number of the neighbors of node $v_{i}$ with node type t+1. Then, similar to DeepWalk and Node2Vec models, they used the skip-gram model to learn node embeddings. The approach from JUST [159] was conceptually similar to Metapath2Vec but the sampling strategy is performed differently. The model introduced a biased random-walk strategy with two parameters (jumping and staying) which aims to change the current domain or stay in the same domain for the next step.

Since the vanilla meta-path sampling strategy fails to capture different types of graphs, such as multiplex graphs and sparse graphs, several sampling strategies have been proposed for heterogeneous graphs based on meta-path strategies. The work of Zhang et al. [160] was similar to Metapath2Vec which implements random-walk sampling of all node types in the multiplex network. Lee et al. [161] introduced a BHIN2vec model which uses the random-walk strategy to capture sparse and rare patterns in heterogeneous graphs. Some models [162,163,164,165] have been applied to biological areas based on random-walk strategies. Lee et al. [166] used the WL relabeling strategy to capture temporal substructures of graphs. The model targeted the proximity of substructures in graphs instead of node proximity to learn the bibliographic entities in heterogeneous graphs. There are several models [167,168,176,177] that aim to capture entities from multiple networks. Du and Tong et al. [167] presented the MrMine model (Multi-resolution Multi-network) to learn embeddings with multi-resolutions. They first used WL label transformation to label nodes by the degree sequences, then adopted a dynamic time wrapping measure [21] to calculate the distance of each sequence to generate a relation network. The truncated random-walk sampling strategy is adopted to capture the graph structure. In contrast to the MrMine model, Lee and colleagues [168,176,177] explored in-depth multi-layered structure to represent the relation and proximity of individual characters, substructures, and the story network as a whole. To embed the substructure and story network, they first used WL relabeling [33] to extract substructures in the story network and then used Subgraph2Vec and Doc2Vec models to learn node embeddings.

**Figure 10:**
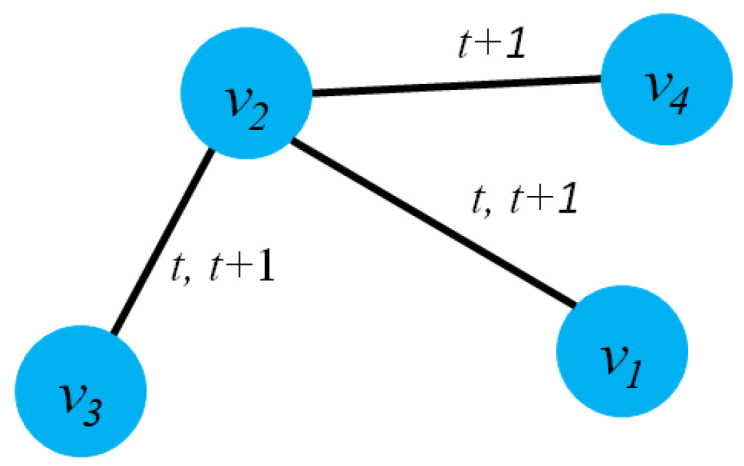
Updating random-walk paths to the corpus on dynamic graphs. At time *t*, the graph has 3 nodes: $v_{1},v_{2},v_{3}$ with two edges:$\left(v_{1},v_{2}\right)$ and $\left(v_{2},v_{3}\right)$. Assuming the length of the random walk is 3, then the set of random walks: $\langle v_{1},v_{2},v_{1}\rangle$, $\langle v_{1},v_{2},v_{3}\rangle$, $\langle v_{2},v_{1},v_{2}\rangle$, $\langle v_{2},v_{3},v_{2}\rangle$, $\langle v_{3},v_{2},v_{1}\rangle$, $\langle v_{3},v_{2},v_{3}\rangle$. At the time t+1: The graph has a new node $v_{4}$ and a new edge $\left(v_{2},v_{4}\right)$. Then, new random walks will be updated on the corpus are: $\langle v_{4},v_{2},v_{1}\rangle$, $\langle v_{4},v_{2},v_{3}\rangle$, and $\langle v_{4},v_{2},v_{4}\rangle$.

Several types of graphs in the real world, however, show dynamic behaviors. Since most graph embedding models aim to learn node embeddings in static graphs, several models have been applied to learn node embeddings in dynamic graphs [10,92,173,174,175]. Most of them were based on the idea of DeepWalk and Node2Vec to capture the graph structure. By representing dynamic graphs as a set of static graphs, some models captured changes in the dynamic graph structure and updated changes in random walks over time. Then, the skip-gram model is used to learn node embeddings. For instance, the key idea of Sajjad et al. [169] is to generate random-walk paths on the first snapshot and then update random-walk paths in the corpus by time. Most existing models re-generate node embeddings for each graph snapshot to capture the dynamic behaviors. By contrast, the model introduced a set of dynamic random walks, which are frequently updated when there are any changes in dynamic graphs. This could reduce the computational complexity when the model handles large graphs. Figure 10 shows an example of how random-walk paths are updated in dynamic graphs.

Since the evolution of graphs only takes place at every few nodes and within a specific range of neighbors, updating the entire random walk is time-consuming. Several models [169,170,171,172,174,175] suggested updating dynamic steps over time for a few nodes and their local neighbors’ relationship. For example, Sedigheh et al. [174] presented the Dynnode2Vec model to capture the temporal evolution from graph $G_{t}$ to $G_{t+1}$ by a set of new nodes and edges $\left(V_{new},E_{new}\right)$ and a set of removed nodes and edges $\left(V_{del},E_{del}\right)$. Motivated by Node2Vec architecture, the Dynnode2Vec model could learn the dynamic structure by inducing an adequate group of random walks for only dynamic nodes. The random-walk strategy, therefore, could be more computational efficiency when the model handles large graphs. Furthermore, the proposed dynamic skip-gram model could learn node embeddings at time *t* by adopting the results of the previous time t−1 as initial weights. As a result, the dynamic skip-gram model could learn the dynamic behaviors over time.

Therefore, the changes in nodes at time t+1 could be described as:

(27) $$\Delta V_{t}=V_{add}\cup \left\{v_{i}\in V_{t+1}\right|\exists e=\left(v_{i},v_{j}\right)\in \left(E_{add}\cup E_{del}\right)\}\;.$$

In summary, structure-preservation methods have succeeded in learning embeddings over the past decade. There are several key advantages of these models:

- Computational complexity: Unlike kernel models and matrix factorization-based models, which require considerable computational costs, structure preservation models could learn embeddings with an efficient time. This effectiveness comes from search-based sampling strategies and the model generalizability from the training process.
- Classification tasks: Since the models aim to find structural neighbor relationships from a target node, these show power in problems involving node classification. In almost all graphs, nodes that have the same label tend to be connected at a small, fixed-length distance. This is a strength of models based on preserving structure in problems related to classification tasks.

However, there are a few limitations that these models suffer when preserving the graph structure:

- Transductive learning: Most models cannot learn node embeddings that have not been seen in the training data. To learn new node embeddings, the model should re-sample the graph structure and learn the new samples again which could be time-consuming.
- Missing connection problem: Many graphs have sparse connections and missing connections between nodes in the real world. However, most structure-preservation models cannot handle missing connections between nodes since the sampling strategies could not be able to capture these connections. In the case of a random-walk-based sampling strategy, for example, these models only capture graph structure when nodes are linked together.
- Parameter sharing: These models could only learn node embeddings for individual nodes and do not share parameters. The absence of sharing parameters could reduce the effectiveness of learning representation.

#### 3.3.2. Proximity Reconstruction Models

The purpose of graph embedding models is not only to preserve the graph structure but also to preserve the proximity of nodes in graphs. Most proximity reconstruction-based models are used for link prediction or node recommendation tasks [178,179,180] due to the nature of the similarity strategies. In this part, we discuss various models attempting to preserve the proximity of entities in graphs. Table 7 describes a summary of representative proximity reconstruction-based graph embedding models.

One of the typical models is LINE [16], which aims to preserve the symmetric proximity of node pairs in graphs. The advantage of the LINE model is that it could learn the node similarity which most structure-preservation models cannot represent this structural information. The main goal of the LINE model is to preserve the 1st-order and 2nd-order proximity of node pairs in graphs. The 1st-order proximity can be defined as follows:

**Definition** **7** (1st-order proximity [16]). *The 1st-order proximity describes the local pairwise similarity between two nodes in graphs. Let $w_{ij}$ be the weight of an edge between two nodes $v_{i}$ and $v_{j}$, and the 1st-order proximity is defined as $w_{ij}$ when two nodes are connected and $w_{ij}=0$ when there is no link between them.*

In the case of binary graphs, $w_{ij}=1$ if two nodes $v_{i}$ and $v_{j}$ are connected, and $w_{ij}=0$ otherwise. To preserve the 1st-order proximity, the objective function of two distribution ${\hat{p}}_{1}\left(v_{i},v_{j}\right)$ and $p_{1}\left(v_{i},v_{j}\right)$ should be minimized:

(28) $$\begin{array}{cc} \; & L_{1}\left(\theta \right)=\underset{\theta }{\arg\min}\;d\left({\hat{p}}_{1}\left(v_{i},v_{j}\right),p_{1}\left(v_{i},v_{j}\right)|\theta \right)\; \end{array}$$

(29) $$\begin{array}{cc} \; & {\hat{p}}_{1}\left(v_{i},v_{j}\right)=\frac{w_{ij}}{\sum_{(v_{k},v_{l})\in E}Z_{k}^{⊺}Z_{l}^{}}\;\;\;p_{1}\left(v_{i},v_{j}\right)=\frac{\exp(Z_{i}^{⊺}Z_{j}^{})}{\sum_{(v_{k},v_{l})\in E}Z_{k}^{⊺}Z_{l}^{}}\; \end{array}$$

where ${\hat{p}}_{1}\left(v_{i},v_{j}\right)$ and $p_{1}\left(v_{i},v_{j}\right)$ depict the empirical probability, and the actual probability of the 1st-order proximity, respectively, $v_{i}$ and $v_{j}$ are two nodes in *G*, $Z_{i}$ and $Z_{j}$ are embedding vectors in latent space corresponding to $v_{i}$ and $v_{j}$, respectively, $d\left(\cdot \;\cdot \right)$ is the distance between the two distributions. The statistical distance, Kullback–Leibler divergence [181], is usually used to measure the difference between two distributions. In addition to preserving the proximity of two nodes that are connected directly, the LINE model also introduced 2nd-order proximity, which could be defined as follows:

**Definition** **8** (2nd-order proximity [16]). *The 2nd-order proximity when k=2 captures the relationship of neighbors of each pair of nodes in the graph G. The idea of the 2nd-order proximity is that nodes should be closed if they share the same neighbors.*

Let $Z_{i}$ and $Z_{j}$ are vector embeddings of nodes $v_{i}$ and $v_{j}$, respectively, the probability of the specific context $v_{j}$ given by the target node $v_{i}$ could be defined as:

(30) $$p_{2}\left(v_{j}|v_{i}\right)=\frac{\exp(Z_{j}^{⊺}Z_{i})}{\sum_{v_{k}\in V}\exp(Z_{k}^{⊺}Z_{i})}\;.$$

Therefore, the minimization of the objective function $L_{2}$ could be defined as:

(31) $$L_{2}\left(\theta \right)=\underset{\theta }{\arg\min}\;\sum_{v_{i}\in V}D_{KL}\left({\hat{p}}_{2}\left(.|v_{i};\theta \right),p_{2}\left(.|v_{i}\right)\right)\;$$

where ${\hat{p}}_{2}\left(v_{j}|v_{i}\right)=\frac{w_{ij}}{\sum_{k\in N(i)}w_{ik}}$ is the observed distribution, $w_{ij}$ is the weighted edge between $v_{i}$ and $v_{j}$.

**Table 7:** A summary of proximity reconstruction models. $v_{i}^{\left(t\right)}$ denotes the type *t* of node $v_{i}$, $w_{ij}$ is the weight between node $v_{i}$ and $v_{j}$, *P* is a meta-path in heterogeneous graphs, $N_{2}$ is the 1st-order and 2nd-order proximity of a node $v_{i}$, and $P_{n}\left(v\right)$ is the noise distribution for negative sampling.

Models	Graph Types	Objective	Loss Function
LINE [16]	Static graphs	Node classification	$-\sum_{v_{i}\in V,\langle v_{i},v_{j}\rangle \in E}\log\left(\sigma (Z_{i}^{⊺}Z_{j})\right)-\left|N_{neg}\right|\sum_{v_{k}∼P_{n}\left(v\right)}\log\left(\sigma (-Z_{i}^{⊺}Z_{k})\right)$
APP [76]	Static graphs	Link prediction	$-\sum_{v_{i}\in V,\langle v_{i},v_{j}\rangle \in E}\log\left(\sigma (Z_{i}^{⊺}Z_{j})\right)-\left|N_{neg}\right|\sum_{v_{k}∼P_{n}\left(v\right)}\log\left(\sigma (-Z_{i}^{⊺}Z_{k})\right)$
PALE [77]	Static graphs	Link prediction	$-\sum_{v_{i}\in V,\langle v_{i},v_{j}\rangle \in E}\log\left(\sigma (Z_{i}^{⊺}Z_{j})\right)-\left|N_{neg}\right|\sum_{v_{k}∼P_{n}\left(v\right)}\log\left(\sigma (-Z_{i}^{⊺}Z_{k})\right)$ $\sum_{\langle v_{i},v_{j}\rangle \in E}{∥Z_{i}-Z_{j}∥}_{F}^{2}$
CVLP [182]	Attributed graphs	Link prediction	$-\sum_{\begin{array}{c} (v_{i},v_{j})\in E\\ (v_{i},v_{k})\notin E \end{array}}\log\sigma \left(Z_{i}^{⊺}Z_{j}-Z_{i}^{⊺}Z_{k}\right)+\alpha_{1}{∥Z_{i}-Z_{j}∥}_{2}^{2}+\alpha_{2}L_{1}+\alpha_{3}L_{2}$
[183]	Static graphs	Link prediction	$-\sum_{\langle v_{i},v_{j}\rangle \in E}w_{ij}\log\left(p_{1}\left(v_{i}|v_{j}\right)\right)-\sum_{\langle v_{i},v_{j}\rangle \in E}w_{ij}\log\left(p_{2}\left(v_{j}|v_{i}\right)\right)$
HARP [178]	Static graphs	Node classification	$-\sum_{v_{i}\in V,\langle v_{i},v_{j}\rangle \in E}\log\left(\sigma (Z_{i}^{⊺}Z_{j})\right)-\left|N_{neg}\right|\sum_{v_{k}∼P_{n}\left(v\right)}\log\left(\sigma (-Z_{i}^{⊺}Z_{k})\right)$
PTE [179]	Heterogeneous graphs	Link prediction	$-\sum_{\langle v_{i}^{\left(t\right)},v_{j}^{\left(t\right)}\rangle \in E^{\left(t\right)}}w_{ij}^{}\log\left(v_{i}^{\left(t\right)}|v_{j}^{\left(t\right)}\right)$
Hin2Vec [180]	Heterogeneous graphs	Node classification, link prediction	$\sum_{v_{i}\in V}-y_{i}^{⊺}\log\left({\hat{y}}_{i}\right)-\left(1-y_{i}\right)\log\left(1-{\hat{y}}_{i}\right)$
[78]	Heterogeneous graphs	Node classification	$\sum_{(v_{i},v_{j})\in E}\log\sigma \left(Z_{i}Z_{j}\right)+\left|N_{neg}\right|E_{v_{k}∼P_{n}\left(v_{k}\right)}\left(\log\sigma (-Z_{i}Z_{k})\right)$
[184]	Signed graphs	Link prediction	$\sum_{v_{i}\in V}-y_{i}^{⊺}\log\left({\hat{y}}_{i}\right)-\left(1-y_{i}\right)\log\left(1-{\hat{y}}_{i}\right)$
[185]	Heterogeneous graphs	Node classification, Node clustering	$-\sum_{(v_{i},v_{j})\in P}\log\left(1+e^{-Z_{i}Z_{j}}\right)+\left|N_{neg}\right|E_{v_{k}∼P_{n}\left(v_{k}\right)}\left[\log\left(1+e^{-Z_{i}Z_{k}}\right)\right]$
[186]	Heterogeneous graphs	Link prediction	$\sum_{\left(v_{i},v_{j}\right)\in N_{2}}\log\sigma \left(Z_{i}Z_{j}\right)+\left|N_{neg}\right|E_{v_{k}∼P_{n}\left(v_{k}\right)}\left(\log\sigma (-Z_{i}Z_{k})\right)$
[187]	Static graphs	Node classification	$\sum_{\left(v_{i},v_{j}\right)\in N_{2}}\log\sigma \left(Z_{i}Z_{j}\right)+\left|N_{neg}\right|E_{v_{k}∼P_{n}\left(v_{k}\right)}\left(\log\sigma (-Z_{i}Z_{k})\right)$
[188]	Heterogeneous graph	Graph reconstruction, link prediction, node classification	$\sum_{v_{i}\in V}{∥\left(Z_{i}-{\hat{Z}}_{i}\right)⊙B∥}_{2}^{2}+\alpha L_{2}$
ProbWalk [189]	Static graphs	Node classification, link prediction	$\sum_{v_{i}\in V,\langle v_{i},v_{j}\rangle \in E}\log\left(\sigma (Z_{i}^{⊺}Z_{j})\right)-\left|N_{neg}\right|\sum_{v_{k}∼P_{n}\left(v\right)}\log\left(\sigma (-Z_{i}^{⊺}Z_{k})\right)$
[190]	Static graphs	Node classification, link prediction	$\frac{1}{\left|V\right|}\sum_{v_{i}\in V}\left[y_{i}\log{\hat{y}}_{i}+\alpha \left(1-y_{i}\right)\log\left(1-{\hat{y}}_{i}\right)\right]$
NEWEE [191]	Static graphs	Node classification, link prediction	$-\sum_{v_{i}\in V,\langle v_{i},v_{j}\rangle \in E}\log\left(\sigma (Z_{i}^{⊺}Z_{j})\right)-\left|N_{neg}\right|\sum_{v_{k}∼P_{n}\left(v\right)}\log\left(\sigma (-Z_{i}^{⊺}Z_{k})\right)$
DANE [192]	Attributed graphs	Node classification, Link prediction	$\sum_{v_{i}\in V}{∥X_{i}-{\hat{X}}_{i}∥}_{2}^{2}+\sum_{v_{i}\in V}{∥M_{i}-{\hat{M}}_{i}∥}_{2}^{2}-\sum_{(v_{i},v_{j})\in E}\log p_{ij}-\sum_{(v_{i},v_{j})\in E}\left[\log p_{ij}-\sum_{(v_{i},v_{j})\notin E}\log(1-p_{ij})\right]$
CENE [193]	Attributed graphs	Node classification	$\sum_{v_{i}\in V}-y_{i}^{⊺}\log\left({\hat{y}}_{i}\right)-\left(1-y_{i}\right)\log\left(1-{\hat{y}}_{i}\right)$
HSCA [194]	Attributed graphs	Node classification	${∥M-W^{⊺}HX∥}_{F}^{2}+\alpha \left({∥W∥}_{F}^{2}+{∥H∥}_{F}^{2}\right)$

However, the LINE model had several limitations as it only handles symmetric proximity pairs of nodes, and the proximity of node pairs was only considered up to 2nd-order proximity. To deal with directed graphs, Chang et al. [76] introduced the APP model, which could preserve the asymmetric proximity of node pairs. They introduced two roles for each node $v_{i}\in V$ as the source role $s_{v_{i}}$ and target role $t_{v_{i}}$. The probability of each pair of nodes that start from a source node to the target node could be defined as:

(32) $$p\left(v_{i}|v_{j}\right)=\frac{\exp(s_{v_{j}}\cdot t_{v_{i}})}{\sum_{v_{k}\in V}\exp(s_{v_{j}}\cdot t_{v_{k}})}\;.$$

Tong et al. [77] presented the PALE (Predicting Anchor Links via Embedding) model to predict the anchor links in social networks. The idea of the PALE model was the same as that of the LINE model, but they sampled only 1st-order proximity. The loss function with the negative sampling could be defined as:

(33) $$L(V)=-\sum_{(v_{i},v_{j})\in E}\log\sigma \left(Z_{i}Z_{j}\right)-\left|N_{neg}\right|E_{v_{k}∼P_{n}\left(v_{k}\right)}\left(\log\sigma \left(-Z_{i}Z_{k}\right)\right)\;.$$

Wei et al. [182] presented the CVLP (Cross-View Link Prediction) model that could predict the connections of nodes in the context of missing and noisy attributes. Given by a triplet $\left(v_{i},v_{j},v_{k}\right)$ where $(v_{i},v_{j})\in E$ and $(v_{i},v_{k})\notin E$, the probability of proximity preservation is defined as:

(34) $$P\left(s_{ij}>s_{ik}|U^{g}\right)=\sigma \left(s_{ij}-s_{ik}\right)\;$$

where $U^{g}$ is the latent representation, $s_{ij}$ is the inner product of the representation $s_{ij}=U_{i}^{g}{\left(U_{j}^{g}\right)}^{⊺}$, and $\sigma \left(\cdot \right)$ is the sigmoid function. Li et al. [183] performed a similar study to deeply learn follower-ship and followee-ship between users across different social networks. The main idea of this model is that the proximity between nodes in a social network should be preserved in another social network. For each node $v_{i}$ in a graph, there are three vector representations (a node vector $Z_{i}$, an input context vector $Z_{i}^{\left(1\right)}$, and output context vector $Z_{i}^{\left(2\right)}$). In particular, if a node $v_{i}$ is following a node $v_{j}$ in a social network, then vector $Z_{i}$ should contribute to the input context of $Z_{j}^{\left(1\right)}$, and vector $Z_{j}$ should contribute to the output context of $Z_{i}^{\left(2\right)}$. Therefore, given a node $v_{i}$, the input and output context probability of node $v_{j}$ could be defined as follows:

(35) $$\begin{array}{c} p_{input}\left(v_{j}|v_{i}\right)=\frac{\exp(Z{{}_{j}^{\left(1\right)}}^{⊺}Z_{i})}{\sum_{k=1}^{N}\left(Z{{}_{k}^{\left(1\right)}}^{⊺}Z_{i}\right)}\;\;\;p_{output}\left(v_{i}|v_{j}\right)=\frac{\exp(Z{{}_{j}^{\left(2\right)}}^{⊺}Z_{j})}{\sum_{k=1}^{N}\left(Z{{}_{k}^{\left(2\right)}}^{⊺}Z_{j}\right)}\;. \end{array}$$

**Figure 11:**
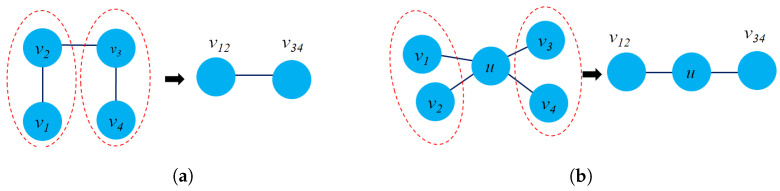
The strategy of edge and node collapsing of HARP model. (**a**) Edge compression; (**b**) Node compression. In (**a**), the super nodes $\langle v_{1},v_{2}\rangle$ and $\langle v_{3},v_{4}\rangle$ are formed by merging edges $e_{12}$ and $e_{34}$, respectively. In (**b**), the super nodes $\langle v_{1},v_{2}\rangle$ and $\langle v_{3},v_{4}\rangle$ are formed by merging node pairs $\left(v_{1},v_{2}\right)$ and $\left(v_{3},v_{4}\right)$, respectively.

Haochen et al. [178] presented HARP (Hierarchical Representation) model with a meta-strategy to capture more global proximity of each pair node in graphs. The critical difference between HARP and LINE models is that they presented the original graph *G* as a series of graphs $G_{1},G_{2},\cdots ,G_{L}$ where each graph can represent the collapse of adjacent edges and nodes. Figure 11 shows the way that two edges and nodes are collapsed in a graph. By representing *L* graphs after multiple collapses of edges and nodes, the graph can compress the proximity of nodes through supernodes.

Several variations and extensions of the LINE model are applied to heterogeneous and dynamic graphs. Jian et al. [179] presented the PTE model to preserve the 1st-order and 2nd-order proximity for heterogeneous graphs. By considering heterogeneous graphs as the set of bipartite graphs, they could independently construct the 1st-order and 2nd-order proximity for each homogeneous graph. Specifically, a bipartite graph *G* could be defined as $G=(V_{A}\cup V_{B},E)$ where $V_{A}$ and $V_{B}$ are the set of nodes with different types. The probability of a node $v_{i}$ in the set $V_{A}$ given by a node $v_{j}$ in the set $V_{B}$ could be defined as follows:

(36) $$p\left(v_{i},v_{j}\right)=\frac{\exp(Z_{i}^{⊺}\cdot Z_{j})}{\sum_{v_{k}\in V_{A}}\exp(Z_{k}^{⊺}\cdot Z_{j})}\;.$$

The PTE model decomposes heterogeneous graphs into *k* homogeneous graphs, and the loss function is the sum of the component loss functions, which could be formulated as:

(37) $$\begin{array}{c} L(V)=-\sum_{\left(v_{i}^{\left(t\right)},v_{j}^{\left(t\right)}\right)\in E^{\left(t\right)}}w_{ij}\log\;p\left(v_{i}^{\left(t\right)}|v_{j}^{\left(t\right)}\right), \end{array}$$

where *K* is the number of bipartite graphs extracted from the heterogeneous graphs. Similar to the PTE model, Tao-yang et al. [180] proposed the Hin2Vec model to capture the 2nd-order proximity in heterogeneous graphs. However, instead of treating heterogeneous graphs as sets of bipartite graphs, the Hin2Vec model captured the relationship between two nodes within 2-hop distance. For instance, in the DBLP network, the relationship set is R={P−P,P−A,A−P,P−P−P,P−P−A,P−A−P,A−P−P,A−P−A} where *P* is the paper node type and *A* is the author node type. Zhipeng and Nikos [185] presented the HINE model (Heterogeneous Information Network Embedding) to preserve the truncated proximity of nodes. They defined an empirical joint probability of two entities in a graph as:

(38) $$\hat{p}\left(v_{i},v_{j}\right)=\frac{s(v_{i},v_{j})}{\sum_{v_{k}\in V}s(v_{i},v_{k})}\;$$

where $v_{i}$ and $v_{j}$ are nodes, and $s(v_{i},v_{j})$ depicts the proximity between $v_{i}$ and $v_{j}$ in *G*. The proximity score $s(v_{i},v_{j})$ could be measured by counting the number of instances of the meta-path containing two nodes or a probability gained from implementing a random-walk sampling from $v_{i}$ to $v_{j}$.

Graphs in the real world, however, could contain attributes where several existing models, such as LINE and APP, fail to capture this information. Several models have been proposed to learn structural similarity in attributed graphs [193,195]. Sun et al. [193] proposed a CENE (content-enhanced network embedding) model to learn structural graphs and side information jointly. The objective of the CENE model is to preserve the similarity between node pairs and node-content pairs. Zhang et al. [194] proposed the HSCA (Homophily, Structure, and Content Augmented network) model to learn the homophily property of node sequences. To gain the node sequences, HSCA uses the DeepWalk model to capture the short random-walk sampling, which could represent the node context. The model then learns node embeddings based on matrix factorization by decomposing the probability transition matrix.

Most models mentioned above mainly consider the edge’s existence and ignore the dissimilarities between edges. Beyond preserving the topology and proximity of the aforementioned nodes, there are a variety of studies on edge reconstruction. The main idea of edge initialization-based models is that the edge weights can be transformed as transition probability. Wu et al. [189] introduced the ProbWalk model to learn weighted edges based on random-walk paths for edges and the skip-gram model to learn edge embeddings. The advantage of random walk on weighted edges is that this could help the model to generate more accurate node sequences and capture more useful structural information. To calculate the probability of weighted edges in graphs, they introduced a joint distribution:

(39) $$p\left(v_{1},v_{2}\;\cdots \;v_{k}|v_{i}\right)=\prod_{v_{j}\in C}\frac{e^{Z_{j}\cdot Z_{i}}}{\sum_{m=1}^{n}e^{Z_{m}\cdot Z_{i}}}\;$$

where $v_{i}$ is the target node, $C=\{v_{1},v_{2},\cdots ,v_{k}\}$ is the context of node $v_{i}$, and $Z_{i}$ is vector embedding of node $v_{i}$.

**Figure 12:**
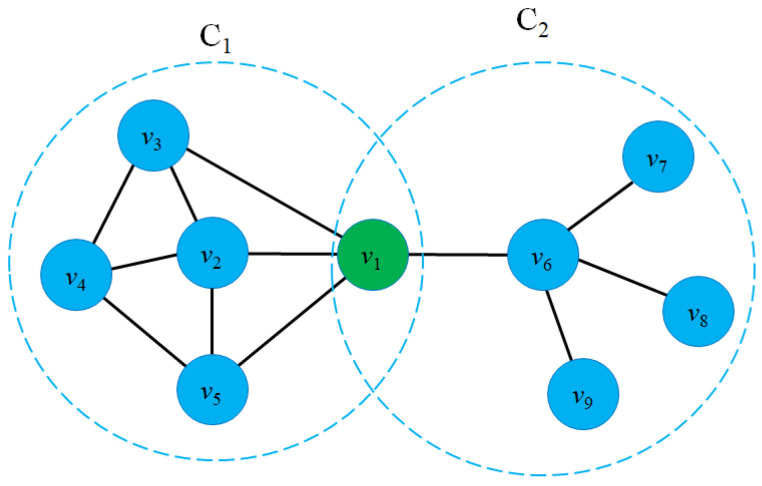
The self-centered network of NEWEE model. For instance, the self-centered of node $v_{2}$ could be defined as $G^{\prime }=\left(V^{\prime },E^{\prime }\right)$ where $V^{\prime }=\left(v_{1},v_{2},v_{3},v_{4},v_{5}\right)$ and $E^{\prime }$ is the set of edges in $G^{\prime }$.

Alternatively, several tasks need to preserve the proximity between different relationship types of nodes. Qi et al. [190,191] proposed the NEWEE model to learn edge embeddings and then adopted a biased random-walk sampling to capture the graph structure. To learn edge embeddings, they first look for a self-centered network of each graph node. In this situation, the model could explore the similarity between edges in the self-centered network since their score tends to be higher than those in the different self-centered networks. Given a node $v_{i}$ in *G*, the self-centered network is a set of nodes containing $v_{i}$ and its neighbors. For example, Figure 12 depicts two self-centered networks $C_{1}$ and $C_{2}$ of node $v_{1}$. The objective of the model is to make all edges embedded in the same self-centered network should be close in the vector space. Therefore, given a self-centered network $G^{\prime }=\left(V^{\prime },E^{\prime }\right)$, the objective function aims to maximize the proximity between edges in the same network, which could be defined as:

(40) $$\begin{array}{c} L\left(E\right)=-\sum_{v_{i}\in V^{\prime }}^{}\sum_{\begin{array}{c} e_{ij}\in E^{\prime }\\ e_{ik}\notin E^{\prime } \end{array}}\log\left[\sigma \left(e_{ij}^{⊺}Z_{i}\right)\right]+\log\left[1-\sigma \left(e_{ij}^{⊺}Z_{k}\right)\right]\; \end{array}$$

where $e_{ij}$ denotes the edge between node $v_{i}$ and $v_{j}$ in a self-centered network $G^{\prime }$, $e_{ik}$ denotes a negative edge that $v_{i}$ and $v_{k}$ coming from different self-centered network.

In summary, compared with structure-preservation models, the proximity construction models bring several advantages:

- Inter-graph proximity: Proximity-based models not only explore proximity between nodes in a single graph but can also are applied for proximity reconstruction across different graphs with common nodes [183]. These methods can preserve the structural similarity of nodes in other graphs which are entirely different from other models. In the case of models based on structure-preservation strategies, these must re-learn node embeddings in other graphs.
- Proximity of nodes belonging to different clusters: In the context of clusters with different densities and sizes, proximity reconstruction-based models could capture nodes that are close to each other but in different clusters. This feature shows an advantage over structure reconstruction-based models, which tend to favor searching for neighboring nodes in the same cluster.
- Link prediction and node classification problem: Since structural identity is based on proximity between nodes, two nodes with similar neighborhoods should be close in the vector space. For instance, the LINE model considered preserving the 1st-order and 2nd-order proximity between two nodes. As a result, proximity reconstruction provides remarkable results for link prediction and node classification tasks [16,76,77].

However, besides the advantages of these models, there are also a few disadvantages of the proximity-based models:

- Weighted edges problems: Most proximity-based models do not consider the weighted edges between nodes. These models consider proximity based only on the number of connections shared without weights which could lead to structural loss.
- Capturing the whole graph structure: Proximity-based models mostly focus on 1st-order and 2nd-order proximity which cannot specify the global structure of graphs. A few models try to capture the higher-order proximity of nodes in graphs, but there is a problem with the computational complexity.

To overcome these limitations, shallow models should be replaced by models based on deep neural networks. Deep neural network-based models can better generalize and capture more of graph entity relationships and graph structure.

### 3.4. Deep Neural Network-Based Models

In recent years, large-scale graphs have challenged the ability of numerous graph embedding models. Traditional models, such as shallow neural networks or statistical methods, cannot efficiently capture complex graph structures due to their simple architecture. Recently, there have been various studies on deep graph neural networks, which are exploding rapidly because of their ability to work with complex and large graphs [11,14,23,196]. Based on the model architecture, we separate deep graph neural networks into four main groups: graph autoencoders, recurrent GNNs, convolutional GNNs, and graph transformer models. This section provides a detailed picture of deep neural network-based methods.

Unlike earlier models, most deep neural network-based models adopt the graph structure (represented as *A*) and node attributes/features (represented as *X*) to learn node embeddings. For instance, users in the social network could have text data, such as profile information. For nodes with missing attribute information, the attributes/features could be represented as node degree or one-hot vectors [72].

#### 3.4.1. Graph Autoencoders

Graph autoencoder models are unsupervised learning algorithms that aim to encode graph entities into the latent space and reconstruct these entities from the encoded information. Based on the encoder and decoder architecture, we can classify graph autoencoder models into multilayer perceptron-based models and recurrent graph neural networks.

**Figure 13:**
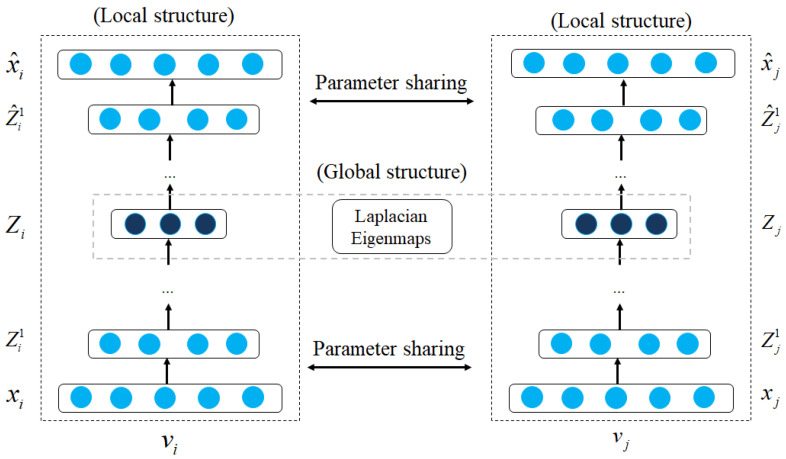
The architecture of SDNE model. The features of nodes $x_{i}$ and $x_{j}$ are the inputs of the SDNE model. The encoder layer compresses the feature data $x_{i}$ and $x_{j}$ into vectors $Z_{i}$ and $Z_{j}$ in the latent space. The decoder layer aims to reconstruct the node features.

Early-stage graph autoencoder models are primarily based on multilayer perceptron (MLP) to learn embeddings [50,51,196]. Table 8 lists a summary of fully connected graph autoencoder models. Daixin et al. [50] introduced the SDNE model (Structural Deep Network Embedding) to capture the graph structure based on autoencoder architecture. Similar to the LINE model, the SDNE model aimed to preserve the 1st-order and 2nd-order proximity between two nodes in graphs, but it used the autoencoder-based architecture. Figure 13 presents the general architecture of the SDNE model with the corresponding encoder and decoder layers. The joint loss function that combines two loss functions for 1st-order proximity and 2nd-order proximity can be formulated as:

(41) $$\begin{array}{cc} L\left(Z,X\right) & =\sum_{i,j=1}^{n}{∥(\hat{X}-X)⊙B∥}_{F}^{2}+\lambda \sum_{i,j=1}^{n}s_{ij}{∥Z_{i}-Z_{j}∥}_{2}^{2}+L_{2}\; \end{array}$$

where $s_{ij}$ denotes the proximity between two nodes $v_{i}$ and $v_{j}$. However, the SDNE model has been proposed to learn node embeddings in homogeneous graphs. Extension of the SDNE model to heterogeneous graphs was suggested by several graph autoencoder models [51,196]. Ke et al. [51] presented the DHNE model (Deep Hyper-Network Embedding) to preserve neighborhood structures, ensuring that the nodes with similar neighborhood structures will have similar embeddings. The autoencoder layer adopts an adjacency matrix *A* of a hypergraph as an input, which can be formulated as:

(42) $$A=HH^{⊺}-D_{v}\;$$

where $D_{v}$ is the diagonal matrix of node degree, and *H* is a matrix of size |V|×|E| presents the relation between nodes and hyperedges in graphs. The autoencoder includes two main layers: an encoder layer and a decoder layer. The encoder part takes the adjacency matrix as input and compresses it to generate node embeddings, and then the decoder part tries to reconstruct the input. Formally, the output of the encoder and decoder layer of node $v_{i}$ could be defined as follows:

(43) $$\begin{array}{cc} \; & Z_{i}=\sigma \left(WA_{i}+b\right)\;\;\;{\hat{A}}_{i}=\sigma \left(\hat{W}Z_{i}+\hat{b}\right)\;. \end{array}$$

One of the limitations of SDNE and DHNE models is that these models cannot handle signed graphs. Shen and Chung [197] proposed the DNE-SBP model (Deep Network Embedding with Structural Balance Preservation) to preserve the proximity of nodes in signed graphs. The DNE-SBP model constructed the input and output of the autoencoder which could be defined as:

(44) $$\begin{array}{c} H^{\left(l\right)}=\sigma \left(X^{\left(l\right)}{\left(W_{1}^{\left(l\right)}\right)}^{T}+B_{1}^{\left(l\right)}\right)\;\;\;{\hat{X}}^{\left(l\right)}=\sigma \left({\hat{H}}^{\left(l\right)}{\left(W_{2}^{\left(l\right)}\right)}^{T}\right)+B_{2}^{\left(l\right)}\; \end{array}$$

where $X^{\left(1\right)}=A$, $X^{\left(l\right)}=H^{\left(l-1\right)}$, and σ is an activation function. The joint loss function is then composed of reconstruction errors with ML and CL pairwise constraints [200].

**Figure 14:**
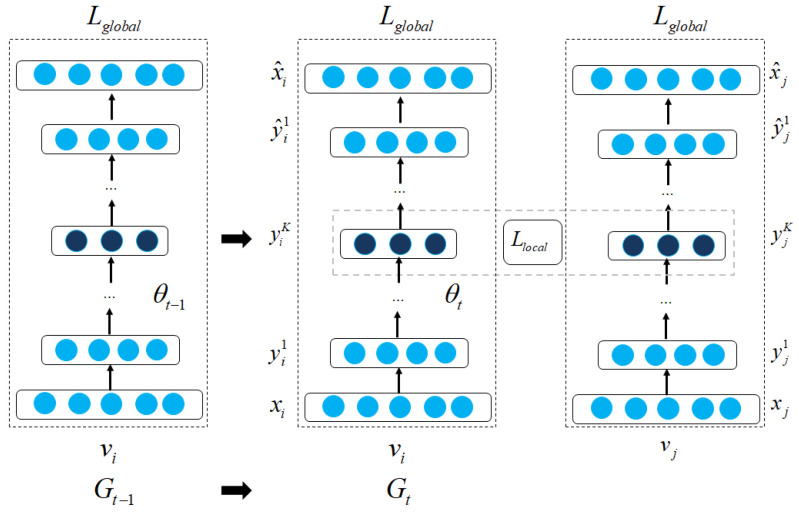
The architecture of DynGEM model. Similarity to the SDNE model, the DynGEM model could capture the 1st-order and 2nd-order proximity between two nodes in graphs with the encoder and decoder layers. The difference is vector embedding $\theta_{t}$ parameters at time *t* are updated from vector embedding $\theta_{t-1}$ at time t−1.

For dynamic graphs, graph autoencoder models take snapshots of graphs as inputs, and the model tries to rebuild snapshots. In several models, the output can predict future graphs by reconstructing coming snapshot graphs. Inspired by the SDNE model for static graphs, Palash et al. [198] presented the DynGEM model for dynamic graph embedding. Figure 14 presents the overview architecture of the DynGEM model. Given a sequence of graph snapshots $G=\{G_{1},G_{2},\cdots ,G_{T}\}$ and a sequence of a mapping function $\phi =\{\phi_{1},\phi_{2},\cdots ,\phi_{T}\}$, the DynGEM model aims to generate an embedding $Z_{t+1}=\phi_{t+1}\left(G_{t+1}\right)$. The stability of embeddings is the ratio of the difference between embeddings over the difference between adjacency matrices over time which could be defined as:

(45) $$A_{abs}\left(\phi ;t\right)=∥\frac{Z_{t+1}\left(V_{t}\right)-Z_{t}\left(V_{t}\right)}{A_{t+1}\left(V_{t}\right)-A_{t}\left(V_{t}\right)}∥\;$$

where $A_{t}$ is the weighted adjacency matrix of graph $G_{t}$, $Z_{t}\left(V_{t}\right)$ presents embeddings of all nodes $V_{t}$ at time *t*. The model learns parameter θ for each graph snapshot $G_{t}$ at time *t*. Similar to the SDNE model, the loss function of the DynGEM model could be defined as:

(46) $$L\left(Z,X\right)={∥(\hat{A}-A)⊙B∥}_{F}^{2}+\lambda \sum_{i,j=1}^{n}s_{ij}{∥Z_{i}-Z_{j}∥}_{2}^{2}+L_{1}+L_{2}\;$$

where $L_{1}$ and $L_{2}$ are regularization terms to prevent the over-fitting, and $s_{ij}$ is the similarity between $v_{i}$ and $v_{j}$. Similar to SDNE, Palash et al. [201] used autoencoder architecture and adopted the adjacency matrix of graph snapshots as input of the encoder layer. However, they updated parameters $\theta_{t}$ at time *t* based on parameter $\theta_{t-1}$ from the previous graph $G_{t-1}$.

Unlike the aforementioned models, Wenchao et al. [199] presented the NetWalk model that composes initial embeddings first and then updates the embeddings by learning paths in graphs, which are sampled by a reservoir sampling strategy. NetWalk model sampled the graph structure using a random-walk strategy as input to the autoencoder model. If there are any changes in dynamic graphs, the Netwalk model first updates the list of neighbors for each node and corresponding edges and then only learns embeddings again for the changes.

The aforementioned autoencoder models, which are based on feedforward neural networks, only focus on preserving pairs of nodes in graphs. Several models focus on integrating recurrent neural networks and LSTM into the autoencoder architecture, bringing prominent results, which we cover in the following section.

#### 3.4.2. Recurrent Graph Neural Networks

One of the first models applying deep neural networks to graph representation learning was based on graph neural networks (GNNs). The main idea of GNNs is that it considers messages shared between target nodes and their neighbors until a steady balance is acquired. Table 9 summarizes graph recurrent autoencoder models.

Scarselli et al. [44,45] proposed a GNN model which could learn embeddings directly for different graphs, such as acyclic/cyclic and directed/undirected graphs. These models assumed that if nodes are directly connected in graphs, the distance between them should be minimized in the latent space. The GNN models used a data diffusion mechanism to aggregate signals from neighbor nodes (units) to target nodes. Therefore, the state of a node describes the context of its neighbors and can be used to learn embeddings. Mathematically, given a node $v_{i}$ in a graph, the state of $v_{i}$ and its output can be defined as:

(47) $$\begin{array}{c} H_{i}=\sum_{v_{j}\in N\left(v_{i}\right)}f_{w}\left(y_{i},e_{ij},H_{j},y_{j}\right),\;\;Z_{i}=g_{w}\left(H_{i},y_{i}\right), \end{array}$$

where $f_{w}\left(\cdot \;\cdot \;\cdot \;\cdot \right)$ and $g_{w}\left(\cdot \;\cdot \right)$ are transition functions, and $y_{i}$, $e_{ij}$ denote the label of node $v_{i}$, edge $\left(v_{i},v_{j}\right)$, respectively. By considering the state $H_{i}$ that is revised by the shift process, $H_{i}$ and its output at layer *l* could be defined as:

(48) $$\begin{array}{c} {H_{i}}^{\left(l\right)}=f_{w}\left(y_{i},e_{ij},{H_{j}}^{\left(l-1\right)},y_{j}\right),\;\;{Z_{i}}^{\left(l\right)}=g_{w}\left({H_{i}}^{\left(l\right)},y_{i}\right)\;. \end{array}$$

However, one of the limitations of GNNs is that the model learns node embeddings as single output, which could cause problems with sequence output. Several studies tried to improve GNNs using recurrent graph neural networks [17,48,49]. Unlike the GNNs which could represent a single output for each entity in a graph, Li et al. [17] attempted to output sequences by applying gated recurrent units. The model used two gated graph neural networks $F_{x}^{\left(l\right)}$ and $F_{o}^{\left(l\right)}$ to predict the output $O^{l}$ and the following hidden states. Therefore, the output of node $v_{i}$ at layer l+1 could be computed as:

(49) $$\begin{array}{c} H_{i}^{\left(l+1\right)}=\sigma \left(H_{i}^{\left(l\right)},\sum_{v_{j}\in N\left(v_{i}\right)}WH_{j}^{\left(l\right)}\right), \end{array}$$

where $N(v_{i})$ denotes the set of neighbors of node $v_{i}$.

**Figure 15:**
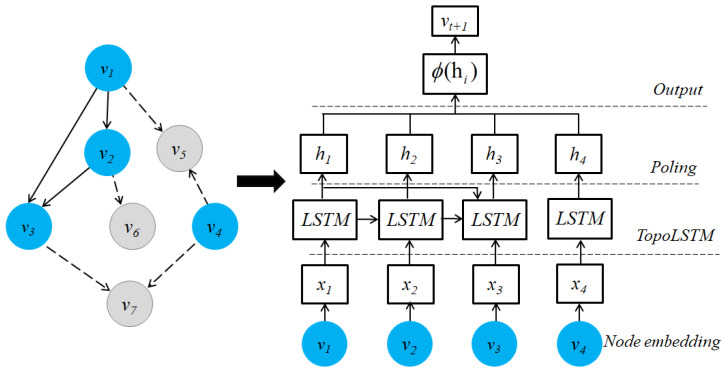
An example of the Topo-LSTM model. Given by a cascade sequence $S=\{\left(v_{1},1\right),\left(v_{2},2\right),\left(v_{3},3\right),\left(v_{4},4\right)\}$, the model first takes features of each node $x_{1}$, $x_{2}$, $x_{3}$, $x_{4}$ as inputs and then infers embeddings via Topo-LSTM model.

Wang et al. [49] proposed Topo-LSTM model to capture the diffusion structure by representing graphs as a diffusion cascade to capture active and inactive nodes in graphs. Given by a cascade sequence $s=\{\left(v_{1},1\right)\;\cdots \;\left(v_{T},T\right)\}$, the hidden state can be represented as follows:

(50) $$\begin{array}{cc} \; & h_{t}^{{}^{\prime }\left(p\right)}=\phi \left(h_{v}|v\in P_{v}\right), \end{array}$$

(51) $$\begin{array}{cc} \; & h_{t}^{{}^{\prime }\left(q\right)}=\phi (h_{v}|v\in Q_{v}\backslash P_{v}), \end{array}$$

where *p* and *q* denote the input aggregation for active nodes connected with $v_{t}$ and not connected with the node $v_{t}$, respectively, $P_{v}$ depicts the precedent sets of active nodes at time *t*, and $Q_{v}$ depicts the set of activated nodes before time *t*. Figure 15 presents an example of the Topo-LSTM model. However, these models could not capture global graph structure since they only capture the graph structure within *k*-hop distance. Several models have been proposed by combining graph recurrent neural network architecture with random-walk sampling structure to capture higher structural information [48,93]. Huang et al. [93] introduced the GraphRNA model to combine a joint random-walk strategy on attributed graphs with recurrent graph networks. One of the powers of the random-walk sampling strategy is to capture the global structure. By considering the node attributes as a bipartite network, the model could perform joint random walks on the bipartite matrix containing attributes to capture the global structure of graphs. After sampling the node attributes and graph structure through joint random walks, the model uses graph recurrent neural networks to learn embeddings. Similar to GraphRNA model, Zhang et al. [48] presented the SHNE model to analyze the attributes’ semantics and global structure in attributed graphs. The SHNE model also used a random-walk strategy to capture the global structure of graphs. However, the main difference between SHNE and GraphRNA is that the SHNE model first applied GRU (gated recurrent units) model to learn the attributes and then combined them with graph structure via random-walk sampling.

**Figure 16:**
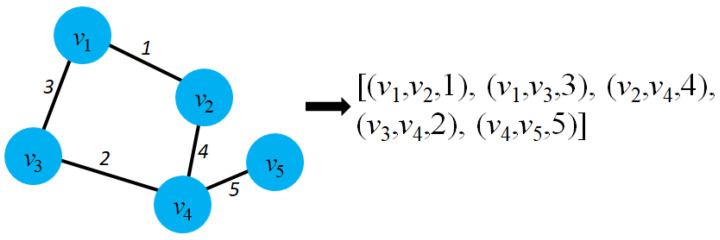
The sampling strategy of [57]. The model lists all the node pairs in respective weights as input of the autoencoder model.

Since the power of autoencoders architecture is to learn compressed representations, several studies [57,205] aimed to combine RGNNs and autoencoders with learning node embeddings in weighted graphs. For instance, Seo and Lee [57] adopted an LSTM autoencoder to learn node embeddings for weighted graphs. They used the BFS algorithm to travel nodes in graphs and extract the node-weight sequences of graphs as inputs for the LSTM autoencoder. The model then could leverage the graph structure reconstruction based on autoencoder architecture and the node attributes by the LSTM model. Figure 16 presents the sampling strategy of this model, which lists the nodes and their respective weighted edges. To capture local and global graph structure, Aynaz et al. [205] proposed a sequence-to-sequence autoencoder model, which could represent inputs with arbitrary lengths. The LSTM-based autoencoder model architecture consists of two main parts: The encoder layer ${\mathrm{LSTM}}_{enc}$ and the decoder layer ${\mathrm{LSTM}}_{dec}$. For the sequence-to-sequence autoencoder, at each time step *l*, the hidden vectors in the encoder and decoder layers can be defined as:

(52) $$\begin{array}{c} h_{enc}^{t}={\mathrm{LSTM}}_{enc}\left(Z_{i}^{\left(t\right)},h_{enc}^{t-1}\right),\;\;h_{dec}^{t}={\mathrm{LSTM}}_{dec}\left(Z_{i}^{\left(t-1\right)},h_{dec}^{t-1}\right)\; \end{array}$$

where $h_{enc}^{t}$ and $h_{dec}^{t}$ are the hidden states at step *t* in the encoder and decoder layers, respectively. To generate the sequences of nodes, the model implemented different sampling strategies, including random walks, shortest paths, and breadth-first search with the WL algorithm to encode the information of node labels.

**Figure 17:**
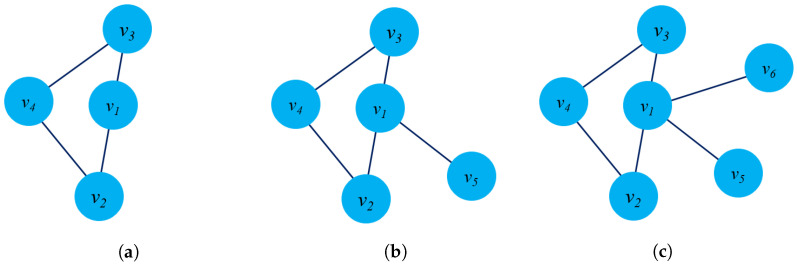
The temporal random-walk sampling strategy of LSTM-Node2Vec model during the graphs’ evolution. (**a**) *t*; (**b**) t+1; (**c**) t+2. At the time *t*, the graph has four nodes and four edges between nodes. At the time t+1 and t+2, the graph has new nodes $v_{5}$ and $v_{6}$, respectively. A temporal random walk for node $v_{1}$ with length L=3 could be: $P=\{\left(v_{2},v_{3},v_{4}\right),\left(v_{3},v_{2},v_{5}\right),\left(v_{3},v_{5},v_{6}\right),\cdots \}$.

Since the aforementioned models learn node embeddings for static graphs, Shima et al. [203] presented an LSTM-Node2Vec model by combining an LSTM-based autoencoder architecture with the Node2Vec model with learning embeddings for dynamic graphs. The idea of the LSTM-Node2Vec model is that it uses an LSTM autoencoder to preserve the history of node evolution with a temporal random-walk sampling. It then adopted the Node2Vec model to generate the vector embeddings for the new graphs. Figure 17 presents a temporal random-walk sampling strategy to travel a dynamic graph.

Jinyin et al. [204] presented the E-LSTM-D model (Encoder-LSTM-Decoder) to learn embeddings for dynamic graphs by combining autoencoder architecture and LSTM layers. Given by a set of graph snapshots $S=\{G_{t-k},G_{t-k+1},\cdots ,G_{t-1}\}$, the objective of the model is to learn a mapping function $\phi :\phi \left(S\right)\to G_{t}$. The model takes the adjacency matrix as the input of the autoencoder model, and the output of the encoder layer could be defined as:

(53) $$\begin{array}{ccc} H_{e,i}^{\left(1\right)} & = & \mathrm{ReLU}\left(W_{e}^{\left(1\right)}s_{i}^{}+b_{i}^{\left(1\right)}\right)\; \end{array}$$

(54) $$\begin{array}{ccc} H_{e,i}^{\left(l\right)} & = & \mathrm{ReLU}\left(W_{e}^{\left(l\right)}b_{e,i}^{\left(l-1\right)}+b_{e}^{\left(l\right)}\right)\; \end{array}$$

(55) $$\begin{array}{ccc} H_{e}^{\left(l\right)} & = & \left[H_{e,0}^{\left(l\right)},H_{e,1}^{\left(l\right)},\cdots ,H_{e,N-1}^{\left(l\right)}\right]\; \end{array}$$

where $s_{i}$ denotes the *i*-th graph in the series of graph snapshots, ReLU(·)=max(0,·) is the activation function. For the decoder layer, the model tried to reconstruct the original adjacency matrix from vector embeddings, which could be defined as follows:

(56) $$\begin{array}{ccc} H_{d}^{\left(1\right)} & = & \mathrm{ReLU}\left(W_{d}^{\left(1\right)}H_{e}+b_{d}^{\left(1\right)}\right)\; \end{array}$$

(57) $$\begin{array}{ccc} H_{d}^{\left(l\right)} & = & \mathrm{ReLU}\left(W_{d}^{\left(l\right)}H_{d}^{\left(l-1\right)}+b_{d}^{\left(l\right)}\right)\; \end{array}$$

where $H_{e}$ depicts the output of the stacked LSTM model, which captures the current graph’s structure $G_{t}$. Similar to E-LSTM-D model, Palash et al. [201] proposed a variant of Dyngraph2Vec model, named Dyngraph2VecAERNN (Dynamic Graph to Vector Autoencoder Recurrent Neural Network) which also considers the adjacency matrix as input for the model. However, the critical difference between the E-LSTM-D model and the Dyngraph2VecAERNN model is that they feed the LSTM layers directly into the encoder part to learn embeddings. The decoder layer is composed of fully connected neural network layers to reconstruct the inputs.

There are several advantages of recurrent graph neural networks compared to shallow learning techniques:

- Diffusion pattern and multiple relations: RGNNs show superior learning ability when dealing with diffuse information, and they can handle multi-relational graphs where a single node has many relations. This feature is achieved due to the ability to update the states of each node in each hidden layer.
- Parameter sharing: RGNNs could share parameters across different locations, which could be able to capture the sequence node inputs. This advantage could reduce computational complexity during the training process with fewer parameters and increase the performance of the models.

However, one of the disadvantages of the RGNNs is that these models use recurrent layers with the same weights during the weight update process. This leads to inefficiencies in representing different relationship constraints between neighbor and target nodes. To overcome the limitation of RGNNs, convolutional GNNs have shown remarkable ability in recent years when it uses different weights in each hidden layer.

#### 3.4.3. Convolutional Graph Neural Networks

CNNs have achieved remarkable success in the image processing area. Since image data can be considered to be a special case of graph data, convolution operators can be defined and applied to graph mining. There are two strategies to implement when applying convolution operators to the graph domain. The first strategy is based on graph spectrum theory which transforms graph entities from the spatial domain to the spectral domain and applies convolution filters on the spectral domain. The other strategy directly employs the convolution operators in the graph domain (spatial domain). Table 10 summarizes spectral CGNN models.

When computing power is insufficient for implementing convolution operators directly on the graph domain, several studies focus on transforming graph data to the spectral domain and applying filtering operators to reduce computational time [18,55,213]. The signal filtering process acts as the feature extraction on the Laplacian matrix. Most models adopted single and undirected graphs and presented graph data as a Laplacian matrix:

(58) $$\begin{array}{c} L=I_{n}-D^{-\frac{1}{2}}AD^{-\frac{1}{2}}\; \end{array}$$

where *D* denotes the diagonal matrix of the node degree, *A* is the adjacency matrix. The matrix *L* is a symmetric positive definite matrix describing the graph structure. Considering a matrix *U* as a graph Fourier basis, the Laplacian matrix then could be decomposed into three components: $L=U\Lambda U^{⊺}$ where Λ is the diagonal matrix which denotes the spectral representation of graph topology and $U=[u_{0},u_{1},\cdots ,u_{n-1}]$ is eigenvectors matrix. The filter function $g_{\theta }$ resembles a *k*-order polynomial, and the spectral convolution acts as diffusion convolution in graph domains. The spectral graph convolution given by an input *x* with a filter $g_{\theta }$ is defined as:

(59) $$\begin{array}{c} g_{\theta }*x=U_{g_{\theta }}U^{⊺}x\; \end{array}$$

where ∗ is the convolution operation. Bruna et al. [56] transformed the graph data to the spectral domain and applied filter operators on a Fourier basis. The hidden state at the layer *l* could be defined as:

(60) $$\begin{array}{c} H_{i}^{\left(l\right)}=\sigma \left(V\sum_{j=1}^{c_{l-1}}D_{ij}^{\left(l\right)}V^{⊺}H_{j}^{\left(l\right)}\right)\; \end{array}$$

where $D_{ij}^{\left(l\right)}$ is a diagonal matrix at layer *l*, $c_{l-1}$ denotes the number of filters at layer l−1, and *V* denotes the eigenvectors of the *L* matrix. Typically, most of the energy of the *D* matrix is concentrated in the first *d* elements. Therefore, we can obtain the first *d* values of the matrix *V*, and the number of parameters that should be trained is $c_{l-1}\cdot c_{l}\cdot d$.

Several studies focused on improving spectral filters to reduce computational time and capture more graph structure in the spectral domain [210,216]. For instance, Defferrard et al. [216] presented a strategy to re-design convolutional filters for graphs. Since the spectral filter $g_{\theta }\left(\Lambda \right)$ indeed generates a kernel on graphs, the key idea is that they consider $g_{\theta }\left(\Lambda \right)$ as a polynomial which includes *k*-localized kernel:

(61) $$\begin{array}{c} g_{\theta }\left(\Lambda \right)=\sum_{k=0}^{K-1}\theta_{k}\Lambda^{\left(k\right)}\; \end{array}$$

where θ is a vector of polynomial coefficients. This *k*-localized kernel provides a circular distribution of weights in the kernel from a target node to *k*-hop nodes in graphs.

Unlike the above models, Zhuang and Ma [211] tried to capture the local and global graph structures by introducing two convolutional filters. The first convolutional operator, local consistency convolution, captures the local graph structure. The output of a hidden layer $Z^{l}$, then, could be defined as:

(62) $$\begin{array}{c} Z^{\left(l\right)}=\sigma \left({\tilde{D}}^{-\frac{1}{2}}\tilde{A}{\tilde{D}}^{-\frac{1}{2}}Z^{\left(l-1\right)}W^{\left(l\right)}\right)\; \end{array}$$

where $\tilde{A}=A+I$ denotes the self-loops adjacency matrix, and ${\tilde{D}}_{i.i}=\sum_{j}{\tilde{A}}_{ij}$ is the diagonal matrix presenting the degree information of nodes. In addition to the first filter, the second filter aims to capture the global structure of graphs which could be defined as:

(63) $$\begin{array}{c} Z^{\left(l\right)}=\sigma \left(D^{-\frac{1}{2}}APD^{-\frac{1}{2}}Z^{\left(l-1\right)}W^{\left(l\right)}\right)\; \end{array}$$

where *P* denotes the PPMI matrix, which can be calculated via frequency matrix using random-walk sampling.

Most of the above models learn node embeddings by transforming graph data to signal domain and use convolutional filters which lead to increased computational complexity. In 2016, Kipf and Welling [18] introduced graph convolutional networks (GCNs), which were considered to be a bridge between spectral and spatial approaches. The spectral filter $g_{\theta }\left(\Lambda \right)$ and the hidden layers of the GCN model followed the layer-wise propagation rule can be defined as follows:

(64) $$\begin{array}{cc} \; & g_{\theta^{\prime }}\left(\Lambda \right)\approx \sum_{k=0}^{K}{\theta^{\prime }}_{k}T_{k}\left(\tilde{\Lambda }\right)\; \end{array}$$

(65) $$\begin{array}{cc} \; & H^{\left(l+1\right)}=\sigma \left({\tilde{D}}^{-\frac{1}{2}}A{\tilde{D}}^{-\frac{1}{2}}H^{\left(l\right)}W^{\left(l\right)}\right)\; \end{array}$$

where $\tilde{\Lambda }=\frac{2}{\lambda_{\max}}\Lambda -I_{N}$ and $\lambda_{\max}$ is the largest eigenvalue of Laplacian matrix *L*, $\theta^{\prime }\in R^{K}$ is Chebyshev coefficients vector, $T_{k}\left(x\right)$ is Chebyshev polynomials could be defined as:

(66) $$\begin{array}{cc} \; & T_{k}\left(x\right)=2xT_{k-1}\left(x\right)-T_{k-2}\left(x\right)\; \end{array}$$

where $T_{0}\left(x\right)=1\;\mathrm{and}\;T_{1}\left(x\right)=x$. Consequently, the convolution filter of an input *x* is defined as:

(67) $$\begin{array}{c} g_{\theta^{\prime }}*x\approx \sum_{k=0}^{K}{\theta^{\prime }}_{k}T_{k}\left(\tilde{L}\right)x,\;\;\tilde{L}=\frac{2}{\lambda_{\max}}L-I_{N}. \end{array}$$

Although spectral CGNNs are effective in applying convolution filters on the spectral domain, they have several limitations as follows:

- Computational complexity: The spectral decomposition of the Laplacian matrix into matrices containing eigenvectors is time-consuming. During the training process, the dot product of the *U*, Λ, and $U^{T}$ matrices also increase the training time.
- Difficulties for handling large-scale graphs: Since the number of parameters for the kernels also corresponds to the number of nodes in graphs. Therefore, spectral models could not be suitable for large-scale graphs.
- Difficulties for considering graph dynamicity: To apply convolution filters to graphs and train the model, the graph data must be transformed to the spectral domain in the form of a Laplacian matrix. Therefore, when the graph data changes, in the case of dynamic graphs, the model is not applicable to capture changes in dynamic graphs.

Motivated by the limitations of spectral domain-based CGNNs, spatial models apply convolution operators directly to the graph domain and learn node embeddings in an effective way. Recently, various spatial CGNNs have been proposed showing remarkable results in handling different graph structures compared to spectral models [52,95]. Based on the mechanism of aggregation from graphs and how to apply the convolution operators, we divide CGNN models into the following main groups: (i) Aggregation mechanism improvement, (ii) Training efficiency improvement, (iii) Attention-based models, and (iv) Autoencoder-CGNN models. Table 11 and Table 12 present a summary of spatial CGNN models for all types of graphs ranging from homogeneous to heterogeneous graphs.

Gilmer et al. [222] presented the MPNN (Message-Passing Neural Network) model to employ the concept of messages passing over nodes in graphs. Given a pair of nodes $\left(v_{i},v_{j}\right)$, a message from $v_{j}$ to $v_{i}$ could be calculated by a message function $M_{ij}$. During the message-passing phase, a hidden state at layer *l* of a node $v_{i}$ could be calculated based on the message-passing from its neighbors, which could be defined as:

(68) $$\begin{array}{cc} \; & m_{i}^{\left(l+1\right)}=\sum_{v_{j}\in N\left(v_{i}\right)}M_{\left(l\right)}\left(h_{i}^{\left(l\right)},h_{j}^{\left(l\right)},e_{ij}\right), \end{array}$$

(69) $$\begin{array}{cc} \; & h_{i}^{\left(l+1\right)}=\sigma \left(h_{i}^{\left(l\right)},m_{i}^{\left(l+1\right)}\right), \end{array}$$

where $M_{\left(l\right)}$ denotes the message function at layer *l* which could be a MLP function, σ is an activation function, and $N(v_{i})$ denotes the set of neighbors of node $v_{i}$.

Most previous graph embedding models work in transductive learning which cannot handle unseen nodes. In 2017, Hamilton et al. [22] introduced the GraphSAGE model (SAmple and aggreGatE) to generate inductive node embeddings in an unsupervised manner. The hidden state at layer l+1 of a node $v_{i}$ could be defined as:

(70) $$\begin{array}{c} h_{i}^{\left(l+1\right)}={\mathrm{AGG}}_{\left(l+1\right)}\left(\{h_{j}^{\left(l\right)},\forall v_{j}\in N\left(v_{i}\right)\}\right)\; \end{array}$$

where $N(v_{i})$ denotes the set of neighbors of node $v_{i}$, $h_{j}^{\left(l\right)}$ is the hidden state of node $v_{j}$ at layer *l*. The function AGG(··) is a differentiable aggregator function. There are three aggregators (e.g., Mean, LSTM, and Pooling) to aggregate information from neighboring nodes and separate nodes into mini batches. Algorithm 1 presents the algorithm of the GraphSAGE model.
**Algorithm 1:** GraphSAGE algorithm. The model first takes the node features as inputs. For each layer, the model aggregates the information from neighbors and then updates the hidden state of each node $v_{i}$.**Input**: G=(V,E): The graph *G* with set of nodes *V* and set of edges *E*.            $x_{i}$: The input features of node $v_{i}$            *L*: The depth of hidden layers, ∀l∈{1⋯L}            ${\mathrm{AGG}}_{k}$: Differentiable aggregator functions            $N(v_{i})$: The set of neighbors of node $v_{i}$.**Output**: $Z_{i}$: Vector representations for $v_{i}$.$h_{i}^{0}\leftarrow x_{i},\forall v_{i}\in V$
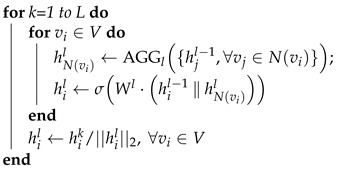
$Z_{i}\leftarrow h_{i}^{L},\;\forall v_{i}\in V$

Lo et al. [231] aimed to apply the GraphSAGE model to detect computer attackers in computer network systems, named E-graphSAGE. The main difference between the two models is that E-graphSAGE used the edges of graphs as aggregation information for learning embeddings. The edge information between two nodes is the data flow between two source IP addresses (Clients) and destination IP addresses (Servers).

By evaluating the contribution of neighboring nodes to target nodes, Tran et al. [229] proposed convolutional filters with different parameters. The key idea of this model is to rank the contributions of different distances from the set of neighbor nodes to target nodes using short path sampling. Formally, the hidden state of a node at layer l+1 could be defined as multiple graph convolutional filters:

(71) $$\begin{array}{c} h^{r,l+1}{\;=\;\|}_{j=0}^{r}\left({\left(D^{j}\right)}^{-1}SP^{j}h^{l}W^{j,l}\right)\; \end{array}$$

where ‖ denotes the concatenation, *r* and $SP^{j}$ denote the *r*-hop distance and the shortest-path distance *j*, respectively. Ying et al. [225] considered random-walk sampling as the aggregation information that can be aggregated to the hidden state of CGNNs. To collect the neighbors of node *v*, the idea of the model is to gather a set consisting of random-walk paths from node *v* and then select the top *k* nodes with the highest probability.

For hypergraphs, several GNN models have been proposed to learn high-order graph structure [27,44,234]. Feng et al. [27] proposed HGNN (Hypergraph Neural Networks) model to learn hypergraph structure based on spectral convolution. They first learn each hyperedge feature by aggregating all the nodes connected by the hyperedge. Then, each node’s attribute is updated with a vector embedding based on all the hyperedges connecting to the nodes. By contrast, Yadati [234] presented the HyperGCN model to learn hypergraphs based on spectral theory. Since each hyperedge could connect several nodes between them, this model’s idea is to filter far apart nodes. Therefore, they adopt the Laplacian operator first to learn node embedding and filter edges, which connect two nodes at a high distance. The GCNs could then be used to learn node embeddings.

One of the limitations of GNN models is that the models consider the set of neighbors as permutation invariant. This limitation then makes the models cannot distinguish between isomorphic subgraphs. By considering the message-passing set from neighbors of nodes as permutation invariant, several works aimed to improve the message-passing mechanism by simple aggregation functions. Xu et al. [24] proposed GIN (Graph Isomorphism Network) model, which aims to learn vector embeddings as powerful as the 1-dimensional WL isomorphism test. Formally, the hidden state of node $v_{i}$ at layer *l* could be defined as:

(72) $$\begin{array}{c} h_{i}^{\left(l\right)}={\mathrm{MLP}}^{\left(l\right)}\left(\left(1+\varepsilon^{\left(l\right)}\right)\cdot h_{i}^{\left(l-1\right)}+\sum_{v_{j}\in N\left(v_{i}\right)}h_{j}^{\left(l-1\right)}\right)\; \end{array}$$

where MLP denotes multilayer perceptions and ε is a parameter that could be learnable or fixed scalar. Another problem of GNNs is the over-smoothing problem when stacking more layers in the models. DeeperGCN [98] was a similar approach that aims to solve the over-smoothing problem by generalized aggregations and skip connections. The DeeperGCN model defined a simple normalized message-passing, which could be defined as:

(73) $$\begin{array}{cc} \; & m_{ij}^{\left(l\right)}=\mathrm{ReLU}\left(h_{i}^{\left(l\right)}+𝟙\left(h_{e_{ij}}^{\left(l\right)}\cdot h_{e_{ij}}^{\left(l\right)}\right)\right)+\varepsilon \; \end{array}$$

(74) $$\begin{array}{cc} \; & h_{i}^{\left(l+1\right)}=\mathrm{MLP}\left(h_{i}^{\left(l\right)}+s\cdot {∥h_{i}^{\left(l\right)}∥}_{2}\cdot \frac{m_{i}^{\left(l\right)}}{{∥m_{i}^{\left(l\right)}∥}_{2}}\right)\; \end{array}$$

where $m_{ij}$ denotes the message-passing from node $v_{j}$ to node $v_{i}$, $h_{e_{ij}}$ is the edge feature of the edge $e_{ij}$, 𝟙(·) presents an indicator procedure which is being 1 if two nodes $v_{i}$ and $v_{j}$ are connected. Le et al. [233] presented the PHC-GNN model, which improves the message-passing compared to the GIN model. The main difference between PHC-GNN and GIN models is that the PHC-GNN model added edge embeddings and a residual connection after the message-passing. Formally, the message-passing and hidden state of a node $v_{i}$ at layer l+1 could be defined as:

(75) $$\begin{array}{cc} \; & {m_{i}}^{\left(l+1\right)}=\sum_{v_{j}\in N\left(v_{i}\right)}\alpha_{ij}^{}\left(h_{i}^{\left(l\right)}+{h_{e}}_{ij}^{\left(l\right)}\right), \end{array}$$

(76) $$\begin{array}{cc} \; & {{\tilde{h}}_{i}}^{\left(l+1\right)}={\mathrm{MLP}}^{\left(l+1\right)}\left(h_{i}^{\left(l\right)}+m_{i}^{\left(l+1\right)}\right), \end{array}$$

(77) $$\begin{array}{cc} \; & {h_{i}}^{\left(l+1\right)}={h_{i}}^{\left(a\right)}+{{\tilde{h}}_{i}}^{\left(l+1\right)}.\; \end{array}$$

A few studies focused on building pre-trained GNN models, which could be used to initialize other tasks [209,246,247]. These pre-trained models are also beneficial to handle the little availability of node labels. For example, the main objective of the GPT-GNN model [247] is to reconstruct the graph structure and the node features by masking the attributes and edges. Given a permutated order, the model maximizes the node attributes based on observed edges and then generates the remaining edges. Formally, the conditional probability could be defined as:

(78) $$\begin{array}{c} p\left(X_{i},E_{i}|X_{<i},E_{<i}\right)=\sum_{m}p\left(X_{i},E_{i,\neg m}|E_{i,m}X_{<i},E_{<i}\right)\cdot p\left(E_{i,m}|X_{<i},E_{<i}\right)\; \end{array}$$

where $E_{i,m}$ and $E_{i,\neg m}$ depict the observed and masked edges, respectively.

**Figure 18:**
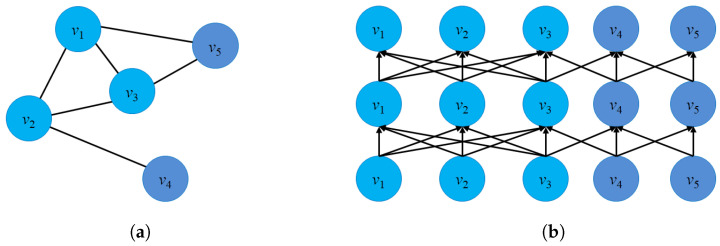
An example of the GraphSAINT model. (**a**) A subgraph has five nodes $v_{1}$, $v_{2}$, $v_{3}$, $v_{4}$, and $v_{5}$; (**b**) A full GCN has three layers. (**a**) presents a subgraph with nodes. In the subgraph, there are 3 nodes $(v_{1}$, $v_{2}$, $v_{3})$ with higher order than the other two nodes ($v_{4}$, $v_{5}$). (**b**) presents a full CGNN-based model with three layers. Three nodes with higher degrees should be sampled from each other in the next layers.

Since learning node embeddings in the whole graphs is time-consuming, several approaches aim to apply standard cluster algorithms (e.g., METIS, K-means, etc.) to cluster nodes into different subgraphs, then use GCNs to learn node embeddings. Chiang et al. [95] proposed a Cluster-GCN model to increase the computational efficiency during the training of the CGNNs. Given a graph *G*, the model first separates *G* into *c* clusters $G=\{G_{1},G_{2},\cdots ,G_{c}\}$ where $G_{i}=\left\{V_{i},E_{i}\right\}$ using Metis clustering algorithm [248]. The model then aggregates information within each cluster. GraphSAINT model [53] had a similar structure to Cluster-GCN and [249] model. GraphSAINT model aggregated neighbor information and samples nodes directly on a subgraph at each hidden layer. The probability of keeping a connection from a node *u* at layer *l* to a node *v* in layer l+1 could be based on the node degree. Figure 18 presents an example of aggregation strategy for the GraphSAINT model. By contrast, Jiang et al. [54] presented a hi-GCN model (hierarchical GCN) that could effectively model the brain network with two-level GCNs. Since individual brain networks have multiple functions, the first level GCN aims to capture the graph structure. The objective of the 2nd GCN level is to provide the correlation between network structure and contextual information to improve the semantic information. The work from Huang et al. [250] was similar to GraphSAGE and FastGCN models. However, instead of using node-wise sampling at each hidden layer, the model provided two strategies: a layer-wise sampling strategy and a skip-connection strategy that could directly share the aggregation information between hidden layers and improve message-passing. The main idea of the skip-connection strategy is to reuse the information from previous layers that could usually be forgotten in dense graphs.

One of the limitations of the CGNNs is that at the hidden layer, the model updates the state of all neighboring nodes. This can lead to slow training and updating because of inactive nodes. Some models aimed to enhance CGNNs by improving the sampling strategy [52,223,224]. For example, Chen et al. [52] presented a FastGCN model to improve the training time and the model performance compared to CGNNs. One of the problems with existing GNN models is scalability which expands the neighborhood and increases computational complexity. The model could learn neighborhood sampling at each convolution layer which mainly focuses on essential neighbor nodes. Therefore, the model could learn the essential neighbor nodes for every batch.

By considering each hidden layer as an embedding layer of independent nodes, FastGCN aims to subsample the receptive area at each hidden layer. For each layer, they chose $t_{k}$ i.i.d. nodes $u_{1}^{\left(l\right)},u_{2}^{\left(l\right)},\cdots ,u_{k}^{\left(l\right)}$ and compute the hidden state which could be defined as:

(79) $$\begin{array}{cc} \; & {\tilde{h}}_{k+1}^{\left(l+1\right)}\left(v\right)=\frac{1}{k}\sum_{j=1}^{k}\tilde{A}\left(v,u_{j}^{\left(l\right)}\right)h_{k}^{\left(l\right)}u_{j}^{\left(l\right)}W_{}^{\left(l\right)}\; \end{array}$$

(80) $$\begin{array}{cc} \; & h_{k+1}^{\left(l+1\right)}\left(v\right)=\sigma \left({\tilde{h}}_{k+1}^{\left(l+1\right)}\left(v\right)\right)\; \end{array}$$

where $\tilde{A}\left(v,u_{j}^{\left(l\right)}\right)$ denotes the kernel, and σ denotes the activation function. Wu et al. [214] introduced SGC (Simple Graph Convolution) model, which could improve 1st-order proximity in the GCN model. The model removed nonlinear activation functions at each hidden layer. Instead, they used a final SoftMax function at the last layer to acquire probabilistic outputs. Chen et al. [224] presented a model to improve the updating of the nodes’ state. Instead of collecting all the information from the neighbors of each node, the model proposed an option to keep track of the activation history states of the nodes to reduce the receptive scope. The model aimed to maintain the history state ${\bar{h}}_{v}^{\left(l\right)}$ for each state $h_{v}^{\left(l\right)}$ of each node *v*.

Similar to [250], Chen et al. [28] presented a GCNII model using an initial residual connection and identity mapping to overcome the over-smoothing problem. The GCNII model aimed to maintain the structural identity of target nodes to overcome the over-smoothing problem. They introduced an initial residual connection $H^{0}$ at the first convolution layer and identity mapping $I_{n}$. Mathematically, the hidden state at layer l+1 could be defined as:

(81) $$\begin{array}{c} H^{\left(l+1\right)}=\sigma \left(\left(\left(1-a_{l}\right)\tilde{P}\cdot H^{\left(l\right)}+a_{l}H^{\left(0\right)}\right)\left(\left(1-b_{l}\right)I_{n}+b_{l}W^{\left(l\right)}\right)\right)\; \end{array}$$

where $\tilde{P}={\tilde{D}}^{-\frac{1}{2}}\tilde{A}{\tilde{D}}^{-\frac{1}{2}}$ denotes the convolutional filter with normalization. Adding two parameters $H^{\left(0\right)}$ and $I_{n}$ is for the purpose of tackling the over-smoothing problem.

Several models aim to maximize the node representation and graph structure by matching a prior distribution. There have been a few studies based on the idea of Deep Infomax [227] from image processing to learn graph embeddings [26,242]. For example, Velickovic et al. [26] introduced the Deep Graph Infomax (DGI) model, which could adopt the GCN as an encoder. The main idea of mutual information is that the model trains the GCN encoder to maximize the understanding of local and global graph structure in actual graphs and minimize that in fake graphs. There are four components in the DGI model, including:

- A corruption function C: This function aims to generate negative examples from an original graph with several changes in structure and properties.
- An encoder $\phi :R^{N\times M}\times R^{N\times N}\to R^{N\times D}$. The goal of function ϕ is to encode nodes into vector space so that $\phi \left(X,A\right)=H=\left\{h_{1},h_{2},\cdots \;h_{N}\right\}$ presents vector embeddings of all nodes in graphs.
- Readout function $R:R^{N\times D}\to R^{D}$. This function maps all embedding nodes into a single vector (supernode).
- A discriminator $D:R^{M}\times R^{M}\to R$ compares vector embeddings against the global vector of the graph by calculating a score between 0 and 1 for each vector embedding.

One of the limitations of the DGI model is that it only works with attributed graphs. Several studies have improved DGI to work with heterogeneous graphs with attention and semantic mechanisms [242,243]. Similar to the DGI model, Park et al. [243] presented the DMGI model (Deep Multiplex Graph Infomax) for attributed multiplex graphs. Given a specific node with relation type *r*, the hidden state could be defined as:

(82) $$\begin{array}{c} H^{\left(r\right)}=\sigma \left({\hat{D}}_{r}^{-\frac{1}{2}}{\hat{A}}^{\left(r\right)}{\hat{D}}_{r}^{-\frac{1}{2}}{\mathrm{XW}}_{}^{r}\right)\; \end{array}$$

where ${\hat{A}}^{\left(r\right)}=A^{\left(r\right)}+\alpha I_{n}$, and ${\hat{D}}_{ii}^{}=\sum_{j}{\hat{A}}_{ij}^{}$, $W_{}^{r}\in R^{n\times d}$ is trainable weights, and σ is the activation function. Similar to the DGI model, the readout function and discriminator can be employed as:

(83) $$\begin{array}{cc} \; & S^{\left(r\right)}=\mathrm{Readout}\left(H^{\left(r\right)}\right)=\sigma \left(\frac{1}{N}\sum_{i=1}^{N}h_{i}^{\left(r\right)}\right)\; \end{array}$$

(84) $$\begin{array}{cc} \; & D\left(h_{i}^{\left(r\right)},S^{\left(r\right)}\right)=\sigma \left(h{{}_{i}^{\left(r\right)}}^{T}M_{}^{\left(r\right)}s_{}^{\left(r\right)}\right)\; \end{array}$$

where $h_{i}^{\left(r\right)}$ is the *i*-th vector of matrix $H^{\left(r\right)}$, $M^{r}$ denotes a trainable scoring matrix, $S^{r}$ is a function with $S^{r}=\sigma \left(\frac{1}{N}\sum_{i=1}^{N}{h_{i}}^{r}\right)$. The attention mechanism is adopted from [251], which could capture the importance of node type to generate the vector embeddings at the last layer. Similarly, Jing et al. [242] proposed HDMI (High-order Deep Multiplex Infomax) model, which is conceptually similar to the DGI model. The HDMI model could optimize the high-order mutual information to process different relation types.

Increasing the number of hidden layers to aggregate more structural information of graphs can lead to an over-smoothing problem [97,252]. Previous models have considered the weights of messages to be the same role in aggregating information from neighbors of nodes. In recent years, various studies have focused on attention mechanisms to extract valuable information from neighborhoods of nodes [19,253,254]. Table 13 presents a summary of attentive GNN models.

Velickovi et al. [19] presented the GATs (graph attention networks) model, one of the first models in applying attention mechanism to graph representation learning. The purpose of the attention mechanism is to compute a weighted message for each neighbor node during the message-passing of GNNs. Formally, there are three steps for GATs which can be explained as follows:

- Attention score: At layer *l*, the model takes a set of features of a node as inputs $h=\{h_{i}\in R^{d}|v_{i}\in V\}$ and the output $h^{\prime }=\left\{{h^{\prime }}_{i}\in R^{d^{\prime }}|v_{i}\in V\right\}$. An attention score measures the importance of neighbor nodes $v_{i}$ to the target node $v_{j}$ could be computed as:
  (85) $$\begin{array}{c} s_{ij}=\sigma \left(a^{⊺}\left(W^{}{h_{i}}^{}∥W{h_{j}}^{}\right)\right)\; \end{array}$$
  where $a\in R^{2d^{\prime }}$, and $W^{\left(k\right)}\in R^{d^{\prime }\times d}$ are trainable weights, ∥ denotes the concatenation.
- Normalization: The score then is normalized comparable across all neighbors of node $v_{i}$ using the SoftMax function:
  (86) $$\begin{array}{c} \alpha_{ij}=\mathrm{SoftMax}\left({s_{ij}}^{}\right)=\frac{\exp\left(s_{ij}\right)}{\sum_{v_{k}\in N(v_{i})}\exp\left(s_{ik}\right)}\;. \end{array}$$
- Aggregation: After normalization, the embeddings of node $v_{i}$ could be computed by aggregating states of neighbor nodes which could be computed as:
  (87) $$\begin{array}{c} {h_{i}}^{\prime }=\sigma \left(\sum_{v_{j}\in N(v_{i})}\alpha_{ij}\cdot Wh_{j}\right)\;. \end{array}$$

Furthermore, the GAT model used multi-head attention to enhance the model power and stabilize the learning strategy. Since the GAT model takes the attention coefficient between nodes as inputs and ranks the attention unconditionally, this results in a limited capacity to summarize the global graph structure.

In recent years, various models have been proposed based on the GAT idea. Most of them aimed to improve the ability of the self-attention mechanism to capture more global graph structures [253,254]. Zhang et al. [253] presented GaAN (Gated Attention Networks) model to control the importance of neighbor nodes by controlling the amount of attention score. The main idea of GaAN is to measure the different weights that come to different heads in target nodes. Formally, the gated attention aggregator could be defined as follows:

(88) $$\begin{array}{cc} \; & {h_{i}}^{\prime }={\mathrm{MLP}}_{\theta }\left(x_{i}⊕\underset{m=1}{\overset{M_{head}}{∥}}\;\left(g_{i}^{\left(m\right)}\sum_{j\in N(v_{i})}w_{ij}^{\left(m\right)}{\mathrm{MLP}}_{\theta^{\left(m\right)}}\left(h_{i}\right)\right)\right)\; \end{array}$$

(89) $$\begin{array}{cc} \; & g_{i}^{}=\left[g_{i}^{\left(1\right)},g_{i}^{\left(2\right)}\;\cdots \;g_{i}^{\left(M_{head}\right)}\right]\; \end{array}$$

where MLP(·) denotes a simple linear transformation, and $g_{i}^{\left(m\right)}$ is the gate value of *m*-th head of node $v_{i}$.

To capture a coarser graph structure, Kim and Oh [258] considered attention based on the importance of nodes to each other. The importance of nodes is based on whether the two nodes are directly connected. By defining the different attention from target nodes to context nodes, the model could solve the permutation equivalent and capture more global graph structure. Based on this idea, they proposed the SuperGAT model with two variants, scaled dot product (SD) and mixed GO and DP (MX), to enhance the attention span of the original model. The attention score $s_{ij}$ between two nodes $v_{i}$ and $v_{j}$ can be defined as follows:

(90) $$\begin{array}{cc} s_{ij,SD}^{\left(l+1\right)} & =\frac{{\left(W^{\left(l+1\right)}h_{i}^{\left(l\right)}\right)}^{⊺}\times W^{\left(l+1\right)}h_{j}^{\left(l\right)}}{\sqrt{d}}\; \end{array}$$

(91) $$\begin{array}{cc} s_{ij,MX}^{\left(l+1\right)} & ={\left(a^{\left(l+1\right)}\right)}^{⊺}\left[W^{\left(l+1\right)}h_{i}^{\left(l\right)}\left|\right|W^{\left(l+1\right)}h_{j}^{\left(l\right)}\right]\cdot \sigma \left({\left(W^{\left(l+1\right)}h_{i}^{\left(l\right)}\right)}^{⊺}\times W^{\left(l+1\right)}h_{j}^{\left(l\right)}\right)\; \end{array}$$

where *d* denotes the number of features at layer l+1. The two attention scores can softly decline the number of nodes that are not connected to the target node $v_{i}$.

Wang et al. [259] aimed to introduce a margin-based constraint to control over-fitting and over-smoothing problems. By assigning the attention weight of each neighbor to target nodes across all nodes in graphs, the proposed model can adjust the influence of the smoothing problem and drop unimportant edges.

Extending the GAT model to capture more global structural information using attention, Haonan et al. [256] introduced the GraphStar model using a virtual node (a virtual start) to maintain global information at each hidden layer. The main difference between the GraphStar and GATs models is that they introduce three different types of relationships: node-to-node (self-attention), node-to-start (global attention), and node-to-neighbors (local attention). Using different types of relationships, GraphStar could solve the over-smoothing problem when staking more neural network layers. Formally, the attention coefficients could be defined as:

(92) $$\begin{array}{c} h_{i}^{\left(t+1\right)}=\underset{m}{\overset{M_{head}}{\left|\right|}}\;\sigma \left(\sum_{r\in R}\sum_{j\in N_{i}^{r}}\alpha_{ijr}^{m}W_{1}^{m(t)}h_{j}^{t}+\alpha_{is,r=s}^{m}W_{2}^{m(t)}S_{}^{t}+\alpha_{i0,r=0}^{m}W_{3}^{m(t)}h_{i}^{t}\right)\; \end{array}$$

where $W_{1}^{m(t)}$, $W_{2}^{m(t)}$, and $W_{3}^{m(t)}$ denotes the node-to-node, node-to-start and node-to-neighbors relations at the *m*-th head of node $v_{i}$, respectively.

One of the problems with the GAT model is that the model only provides static attention which mainly focuses the high-weight attention on several neighbor nodes. As a result, GAT cannot learn universal attention for all nodes in graphs. Motivated by the limitations of the GAT model, Brody et al. [58] proposed the GATv2 model using dynamic attention which could learn graph structure more efficiently from a target node $v_{i}$ to neighbor node $v_{j}$. The attention score can be computed with a slight modification:

(93) $$\begin{array}{c} s_{ij}=a^{⊺}\sigma (W\cdot [h_{i}\left|\right|h_{j}])\;. \end{array}$$

Similar to Wang et al. [259], Zhang et al. [260] presented ADSF (ADaptive Structural Fingerprint) model, which could monitor attention weights from each neighbor of the target node. However, the difference between GraphStar [259] and the ADSF model is that the ADSF model introduced two attention scores $s_{ij}$ and $e_{ij}$ for each node $v_{i}$ which can capture the graph structure and context, respectively.

Besides the GAT-based models applied to homogeneous graphs, several models tried to apply attention mechanism to heterogeneous and knowledge graphs [25,261,262]. For example, Wang et al. [25] presented hierarchical attention to learn the importance of nodes in graphs. One of the advantages of this model is to handle heterogeneous graphs with different types of nodes and edges by deploying local and global level attention. The model proposed two levels of attention: node and semantic-level attention. The node-level attention aims to capture the attention between two nodes in meta-paths. Given a node pair $\left(v_{i},v_{j}\right)$ in a meta-path *P*, the attention score of *P* could be defined as:

(94) $$\begin{array}{c} s_{ij}^{P}={\mathrm{Att}}_{node}\left(h_{i}^{{}^{\prime }},h_{j}^{{}^{\prime }};P\right)\; \end{array}$$

where $h_{i}^{\prime }$ and $h_{j}^{\prime }$ denote the original and projected features of node $v_{i}$ and $v_{j}$ via a projection function $M_{\phi }$, respectively, and ${\mathrm{Att}}_{node}$ is a function which scores the node-level attention. To make the coefficients across other nodes in a meta-path *P* which contain a set of neighbors $N_{i}^{P}$ of a target node $v_{i}$, the attention score $\alpha_{ij}^{P}$, and node embedding with *k* multi-head attention can be defined as:

(95) $$\begin{array}{cc} \; & \alpha_{ij}^{P}=\frac{\exp\left(\sigma (s_{ij}^{T}\cdot [h_{i}^{{}^{\prime }}\left|\right|h_{j}^{{}^{\prime }}])\right)}{\sum_{k\in N_{i}^{P}}\exp\left(\sigma (s_{ik}^{T}\cdot [h_{i}^{{}^{\prime }}\left|\right|h_{k}^{{}^{\prime }}])\right)}\; \end{array}$$

(96) $$\begin{array}{cc} \; & z_{i}^{P}=\underset{k=1}{\overset{K}{\left|\right|}}\;\sigma \left(\sum_{j\in N_{i}^{P}}\alpha_{ij}^{P}{h^{\prime }}_{j}\right)\;. \end{array}$$

The score $z_{i}^{P}$ indicates how the importance of the set of neighbors based on meta-path *P* contributes to node $v_{i}$. Furthermore, the semantic-level aggregation aims to score the importance of meta-paths. Given an attention coefficient $z_{i}^{P}$, the importance of meta-path *P* and its normalization could be defined as $w_{P}$:

(97) $$\begin{array}{cc} \; & w_{P}=\frac{1}{\left|V\right|}\left(\sum_{i\in V}q^{⊺}\cdot \tanh\left(W\cdot z_{i}^{P}+b\right)\right)\; \end{array}$$

(98) $$\begin{array}{cc} \; & {\bar{w}}_{P}=\frac{\exp\left(w_{P}\right)}{\sum_{p=1}^{l}\exp\left(w_{p}\right)}\;. \end{array}$$

In addition to applying CGNNs to homogeneous graphs, several studies focused on applying CGNNs for heterogeneous and knowledge graphs [224,241,243,263,264,266]. Since heterogeneous graphs have different types of edges and nodes, the main problem when applying CGNN models is the aggregation of messages based on different edge types. Schlichtkrull et al. [241] introduced the R-GCNs model (Relational Graph Convolutional Networks) to model relational entities in knowledge graphs. R-GCNs is the first model to be applied to learn node embeddings in heterogeneous graphs to several downstream tasks, such as link prediction and node classification. In addition, they also use parameter sharing to learn the node embedding efficiently. Formally, given a node $v_{i}$ under relation r∈R, the hidden state at layer l+1 could be defined as:

(99) $$\begin{array}{c} h_{i}^{\left(l+1\right)}=\sigma \left(\sum_{r\in R}\sum_{j\in N_{i}^{r}}\frac{1}{c_{i,r}}W_{r}^{\left(l\right)}h_{j}^{\left(l\right)}+W_{0}^{\left(l\right)}h_{i}^{\left(l\right)}\right), \end{array}$$

where $c_{i,r}$ is the normalization constant, and $N_{i}^{r}$ denotes the set of neighbors of node $v_{i}$ with relation *r*. Wang et al. [265] introduced HANE (Heterogeneous Attributed Network Embedding) model to learn embeddings for heterogeneous graphs. The key idea of the HANE model is to measure attention scores for different types of nodes in heterogeneous graphs. Formally, given a node $v_{i}$, the attention coefficients $s_{ij}^{\left(l\right)}$, attention score $\alpha_{ij}^{\left(l\right)}$, and the hidden state $h_{i}^{\left(l+1\right)}$ at layer l+1 could be defined as:

(100) $$\begin{array}{cc} z_{i}^{\left(l\right)} & =W_{i}^{\left(l\right)}x_{i}^{\left(l\right)}\;\;s_{ij}^{\left(l\right)}=(z_{i}^{\left(l\right)}\left|\right|z_{j}^{\left(l\right)})\;\;\alpha_{ij}^{\left(l\right)}=\frac{\exp(s_{ij}^{\left(l\right)})}{\sum_{v_{k}\in N(v_{i})}\exp(s_{ik}^{\left(l\right)})}\; \end{array}$$

(101) $$\begin{array}{cc} h_{i}^{\left(l+1\right)} & =\sigma \left(z_{i}^{\left(l\right)}\right)⊕\left(\sum_{v_{k}\in N(v_{i})}\alpha_{ik}^{\left(l\right)}z_{l}^{\left(l\right)}\right)\; \end{array}$$

where $N(v_{i})$ denotes the set of neighbors of node $v_{i}$, $x_{i}$ denotes the feature of $v_{i}$, and $W_{i}^{\left(l\right)}$ is the weighted matrix of each node type.

Several studies focused on applying CGNNs for recommendation systems [228,267,268,269]. For instance, Wang et al. [267] presented KGCN (Knowledge Graph Convolutional Networks) model to extract the user preferences in the recommendation systems. Since most existing models suffer from the cold start problem and sparsity of user–item interactions, the proposed model can capture users’ side information (attributes) on knowledge graphs. The users’ preferences, therefore, could be captured by a multilayer receptive field in GCN. Formally, given a user *u*, item *v*, $N_{v}$ denotes the set of items connected to *u*, the user–item interaction score could be computed as:

(102) $$\begin{array}{c} {\tilde{\pi }}_{r_{v,e}}^{u}=\frac{\exp(\pi_{r_{v,e}}^{u})}{\sum_{e\in N(v)}\exp(\pi_{r_{v,e}}^{u})},\;\;v_{N(v)}^{u}=\sum_{e\in N(v)}\exp({\tilde{\pi }}_{r_{v,e}}^{u}e)\; \end{array}$$

where $\pi_{r_{v,e}}^{u}$ denotes an inner product where the score between user *u* and relation *r*, *e* is the representation of item *v*.

Since the power of the autoencoder architecture is to learn a low-dimensional node representation in an unsupervised manner, several studies focused on integrating the convolutional GNNs into autoencoder architecture to leverage the power of the autoencoder architecture [72,270]. Table 14 summarizes graph convolutional autoencoder models for static and dynamic graphs.

**Figure 19:**
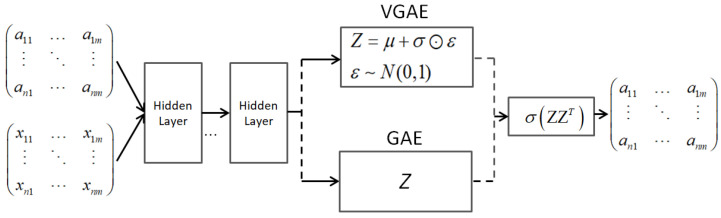
The architecture of GAE and VGAE model. The model adopts the adjacency matrix *A* and the feature matrix *X* as inputs. The encoder part includes two convolutional GNN layers. In the GAE model, the decoder part adopts the embedding matrix *Z* as input and reconstructs the adjacency matrix *A* using an inner product. In the VAGE model, the output of GNN could be represented as a Gaussian distribution.

Most graph autoencoder models were designed based on VAE (variational autoencoders) architecture to learn embeddings [274]. Kipf and Welling [72] introduced the GAE model, one of the first studies on applying autoencoder architecture to graph representation learning. GAE model [72] aimed to reconstruct the adjacency matrix *A* and feature matrix *X* from original graphs by adopting the CGNNs as an encoder and an inner product as the decoder part. Figure 19 presents the detail of the GAE model. Formally, the output embedding *Z* and the reconstruction process of the adjacency matrix input could be defined as:

(103) $$\begin{array}{cc} \; & Z=\mathrm{GCN}\left(X,A\right)\;\;\;\mathrm{error}=\sigma \left(ZZ^{⊺}\right)\; \end{array}$$

where $\mathrm{GCN}\left(\cdot ,\cdot \right)$ function could be defined by Equation (65), and σ is an activation function $\mathrm{ReLU}\left(\cdot \right)=max\left(0,\cdot \right)$. The model aims to reconstruct the adjacency matrix *A* by an inner product decoder part:

(104) $$\begin{array}{c} p\left(A,Z\right)=\prod_{i=1}^{N}\prod_{j=1}^{N}p(A_{ij}|Z_{i},Z_{j}),\;\;p\left(A_{ij}=1|Z_{i},Z_{j}\right)=\sigma \left(Z_{i}^{⊺}Z_{j}\right)\; \end{array}$$

where σ is the sigmoid function and $A_{ij}$ is the value at row *i*-th and column *j*-th in the adjacency matrix *A*. In the training process, the model tries to minimize the loss function by gradient descent:

(105) $$\begin{array}{c} L\left(\theta \right)=E_{q(Z|X,A)}\left[logp(A|Z;\theta )\right]-\mathrm{KL}\left[q(Z|Z,A)||p(Z)\right]\; \end{array}$$

where KL[q(Z|Z,A)||p(Z)] is the Kullback–Leibler divergence between two distributions *p* and *q*.

Several models attempted to incorporate the autoencoder architecture into the GNN model to reconstruct graphs. For example, the MGAE model [270] combined the message-passing mechanism from GNNs and GAE architecture for graph clustering. The primary purpose of MGAE is to capture information about the features of the nodes by randomly removing several noise pieces of information from the feature matrix to train the GAE model.

The GNNs have shown outstanding performance in learning complex structural graphs that shallow models could not solve [245,275,276]. There are several main advantages of deep neural network models:

- Parameter sharing: Deep neural network models share weights during the training phase to reduce training time and training parameters while increasing the performance of the models. In addition, the parameter-sharing mechanism allows the model to learn multi-tasks.
- Inductive learning: The outstanding advantage of deep models over shallow models is that deep models can support inductive learning. This makes deep-learning models capable of generalizing to unseen nodes and having practical applicability.

However, the CGNNs are considered the most advantageous in the line of GNNs and have limitations in graph representation learning.

- Over-smoothing problem: When capturing the graph structure and entity relationships, CGNNs rely on an aggregation mechanism that captures information from neighboring nodes for target nodes. This results in stacking multiple graph convolutional layers to capture higher-order graph structure. However, increasing the depth of convolution layers could lead to over-smoothing problems [252]. To overcome this drawback, models based on transformer architecture have shown several improvements compared to CGNNs using self-attention.
- The ability on disassortative graphs: Disassortative graphs are graphs where nodes with different labels tend to be linked together. However, the aggregation mechanism in GNN samples all the features of the neighboring nodes even though they have different labels. Therefore, the aggregation mechanism is the limitation and challenge of GNNs for disassortative graphs in classification tasks.

#### 3.4.4. Graph Transformer Models

Transformers [277] have gained tremendous success for many tasks in natural language processing [278,279] and image processing areas [280,281]. In documents, the transformer models could tokenize sentences into a set of tokens and represent them as one-hot encodings. With image processing, the transformer models could adopt image patches and use two-dimensional encoding to tokenize the image data. However, the tokenization of graph entities is non-trivial since graphs have irregular structures and disordered nodes. Therefore, applying transformers to graphs is still an open question of whether the graph transformer models are suitable for graph representation learning.

The transformer architecture consists of two main parts: a self-attention module and a position-wise feedforward network. Mathematically, the input of the self-attention model at layer *l* could be formulated as $H=\left[h_{1}^{l},h_{2}^{l},\cdots ,h_{N}^{l}\right]$ where $h_{i}^{l}$ denotes the hidden state of position of node $v_{i}$. Then, the self-attention could be formulated as:

(106) $$\begin{array}{cc} \; & Q=HW_{Q}\;\;K=HW_{K}\;\;V=HW_{V}\; \end{array}$$

(107) $$\begin{array}{cc} \; & S=\frac{QK^{T}}{\sqrt{d_{K}}}\;\;S\left(H\right)=\mathrm{Softmax}\left(S\right)V\; \end{array}$$

where *Q*, *K*, and *V* depict the query matrix, key matrix, and value matrix, respectively, and *d* is the hidden dimension embedding. The matrix *S* measures the similarity between the queries and keys.

The architecture of graph transformer models differs from GNNs. GNNs use message-passing to aggregate the information from neighbor nodes to target nodes. However, graph transformer models use a self-attention mechanism to capture the context of target nodes in graphs, which usually denotes the similarity between nodes in graphs. The self-attention mechanism could help capture the amount of information aggregated between two nodes in a specific context. In addition, the models use a multi-head self-attention that allows various information channels to pass to the target nodes. Transformer models then learn the correct aggregation patterns during training without pre-defining the graph structure sampling. Table 15 lists a summary of graph transformer models.

In this section, we divide graph transformer models for graph representation learning into three main groups based on the strategy of applying graph transformer models.

- Structural encoding-based graph transformer: These models focus on various positional encoding schemes to capture absolute and relative information about entity relationships and graph structure. Structural encoding strategies are mainly suitable for tree-like graphs since the models should capture the hierarchical relations between the target nodes and their parents as well as the interaction with other nodes of the same level.
- GNNs as an auxiliary module: GNNs bring a powerful mechanism in terms of aggregating local structural information. Therefore, several studies try integrating message-passing and GNN modules with a graph transformer encoder as an auxiliary.
- Edge channel-based attention: The graph structure could be viewed as the combination of the node and edge features and the ordered/unordered connection between them. From this perspective, we do not need GNNs as an auxiliary module. Recently, several models have been proposed to capture graph structure in depth as well as apply graph transformer architecture based on the self-attention mechanism.

Several models tried to apply vanilla transformers to tree-like graphs to capture the node position [64,65,277,288]. Preserving tree structure depicts preserving a node’s relative and absolute structural positions in trees. Absolute structural position describes the positional relationship of the current node to the parent nodes (root nodes). In contrast, relative structural position describes the positional relationship of the current node to its neighbors.

Shiv and Quirk [64] proposed to build a positional encoding (PE) strategy for programming language translation tasks. The significant advantage of tree-based models is that they can explore nonlinear dependencies. By custom positional encodings of nodes in the graph in a hieratical manner, the model could strengthen the transformer model’s power to capture the relationship between node pairs in the tree. The key idea is to represent programming language data in the form of a binary tree and encode the target nodes based on the location of the parent nodes and the relationship with neighboring nodes at the same level. Specifically, they used binary matrices to encode the relationship of target nodes with their parents and neighbors.

Similarly, Wang et al. [65] introduced structural position representations for tree-like graphs. However, they combine sequential and structural positional encoding to enrich the contextual and structural language data. The absolute position and relative position encoding for each word $w_{i}$ could be defined as:

(108) $$\begin{array}{cc} \; & {\mathrm{PE}}_{i}=f\left(\frac{\mathrm{Abs}(v_{i})}{{10,000}^{2i/d}}\right)\; \end{array}$$

(109) $$\begin{array}{cc} \; & {\mathrm{PE}}_{ij}=\frac{x_{i}W^{Q}{\left(x_{j}W^{K}\right)}^{⊺}+x_{i}W^{Q}{\left(a_{ij}^{K}\right)}^{⊺}}{\sqrt{d}}\; \end{array}$$

where Abs is the absolute position of the word in the sentence, *d* denotes the hidden size of *K*, *Q* matrix, $f\left(\cdot \right)$ is the sin/cos function depending on the even/old dimension, respectively, and *R* is the matrix presenting relative position representation.

The sentences also are represented in an independent tree which could represent the structural relations between words. For structural position encoding, the absolute and relative structural position of a node $v_{i}$ could be encoded as:

(110) $$\begin{array}{cc} \; & {\mathrm{PE}}_{i}=d\left(v_{i},root\right)\; \end{array}$$

(111) $$\begin{array}{cc} \; & {\mathrm{PE}}_{ij}=\left\{\begin{array}{cc} {\mathrm{PE}}_{i}-{\mathrm{PE}}_{j}\; & \mathrm{if}\;(v_{i},v_{j})\in E.\\ {\mathrm{PE}}_{i}+{\mathrm{PE}}_{j}\; & \mathrm{if}\;(v_{i},v_{j})\notin E,i>j.\\ -({\mathrm{PE}}_{i}+{\mathrm{PE}}_{j})\; & \mathrm{if}\;(v_{i},v_{j})\notin E,i<j.\\ 0\; & \mathrm{otherwise}\;. \end{array}\right.\; \end{array}$$

where d(··) denotes the distance between the root node and the target nodes. They then use a linear function to combine sequential PE and structural PE as inputs to the transformer encoder.

To capture more global structural information in the tree-like graphs, Cai and Lam [282] also proposed an absolute position encoding to capture the relation between target and root nodes. Regarding the relative positional encoding, they use attention score to measure the relationship between nodes in the same shortest path sampled from graphs. The power of using the shortest path is that it can capture the hieratical proximity and the global structure of the graph. Given two nodes $v_{i}$ and $v_{j}$, the attention score between two nodes can be calculated as:

(112) $$\begin{array}{c} S_{ij}=H_{i}W_{q}^{⊺}W_{k}H_{j}\; \end{array}$$

where $W_{q}$ and $W_{k}$ are trainable projection matrices, $H_{i}$ and $H_{j}$ depict the node presentation $v_{i}$ and $v_{j}$, respectively. To define the relationship $r_{i\to j}$ between two nodes $v_{i}$ and $v_{j}$, they adopt a bi-directional GRUs model, which could be defined as follows:

(113) $$\begin{array}{cc} \; & {\overset{\to }{s}}_{i}=\mathrm{GRU}\left({\overset{\to }{s}}_{i-1},SPD_{i\to j}\right)\; \end{array}$$

(114) $$\begin{array}{cc} \; & {\overset{\leftarrow }{s}}_{i}=\mathrm{GRU}\left({\overset{\leftarrow }{s}}_{i+1},SPD_{i\to j}\right)\; \end{array}$$

where SPD denotes the shortest path from node $v_{i}$ to node $v_{j}$, ${\vec{s}}_{i}$ and ${\overset{\leftarrow }{s}}_{i}$ are the states of the forward and backward GRU, respectively.

Several models tried to encode positional information of nodes based on subgraph sampling [63,283]. Zhang et al. [63] proposed a Graph-Bert model, which samples the subgraph structure using absolute and relative positional encoding layers. In terms of subgraph sampling, they adopt a top-*k* intimacy sampling strategy to capture subgraphs as inputs for positional encoding layers. Four layers in the model are responsible for positional encoding. Since several strategies were implemented to capture the structural information in graphs, the advantage of Graph-Bert is that it can be trainable with various types of subgraphs. In addition, Graph-Bert could be further fine-tuned to learn various downstream tasks. For each node $v_{i}$ in a subgraph $G_{i}=\left(V_{i},E_{i}\right)$, they first embed raw feature $x_{i}$ using a linear function. They then adopt three layers to encode the positional information of a node, including absolute role embedding, relative positional embedding, and hop-based relative distance embedding. Formally, the output of three embedding layers of the node $v_{i}$ from subgraph $G_{i}$ could be defined as follows:

(115) $$\begin{array}{cc} \; & {\mathrm{PE}}_{i}^{\left(1\right)}=f\left(WL(v_{i})\right), \end{array}$$

(116) $$\begin{array}{cc} \; & {\mathrm{PE}}_{i}^{\left(2\right)}=f\left(P(v_{i})\right), \end{array}$$

(117) $$\begin{array}{cc} \; & {\mathrm{PE}}_{i}^{\left(3\right)}=f\left(H(v_{j},v_{i})\right), \end{array}$$

(118) $$\begin{array}{cc} \; & f\left(x_{i}\right)={\left[sin\left(\frac{x_{i}}{{10,000}^{2l/d}}\right),cos\left(\frac{x_{i}}{{10,000}^{2l+1/d}}\right)\right]}_{l=0}^{\left\lfloor \frac{d}{2}\right\rfloor }, \end{array}$$

where $WL(v_{i})$ denotes the WL code that labels node $v_{i}$, which can be calculated from whole graphs, *l* and *d* are the numbers of interactions throughout all nodes, and the vector dimension of nodes, P(·) is a position metric, H(··) denotes the distance metric between two nodes, and ${\mathrm{PE}}_{i}^{\left(1\right)}$, ${\mathrm{PE}}_{i}^{\left(2\right)}$, ${\mathrm{PE}}_{i}^{\left(3\right)}$ denote the absolute, relative structure intimacy, and relative structure hop PE, respectively. They then aggregate all the vector embeddings together as initial embedding vectors for the graph transformer encoder. Mathematically, the transformer architecture could be explained as follows:

(119) $$\begin{array}{cc} \; & h_{i}^{\left(0\right)}={\mathrm{PE}}_{i}^{\left(1\right)}+{\mathrm{PE}}_{i}^{\left(2\right)}+{\mathrm{PE}}_{i}^{\left(3\right)}+X_{i}\; \end{array}$$

(120) $$\begin{array}{cc} \; & H^{\left(l\right)}=\mathrm{Transformer}\left(H^{\left(l-1\right)}\right)\; \end{array}$$

(121) $$\begin{array}{cc} \; & Z_{i}=\mathrm{Fusion}\left(H^{\left(l\right)}\right)\;. \end{array}$$

Similar to Graph-Bert, Jeon et al. [283] tried to present subgraphs for the paper citation network and capture the contextual citation of each paper. Each paper is considered a subgraph with nodes as reference papers. To extract the citation context, they encode the order of the referenced papers in the target paper based on the position and order of the referenced papers. In addition, they use the WL label to capture the structural role of the references. The approach by Liu et al. [289] was conceptually similar to [283]. However, there is a significant difference between them. They proposed an MCN sampling strategy to capture the contextual neighbors from a subgraph. The purpose of MCN sampling is based on the importance of the target node based on the frequency of occurrence when sampling.

In several types of graphs, such as molecular networks, the edges could bring features presenting the chemical connections between atoms. Several models adopted Laplacian eigenvectors to encode the positional node information with edge features [29,284]. Dwivedi and Bresson [29] proposed the positional encoding strategy using node position and edge channel as inputs to the transformer model. The idea of this model is to use Laplacian eigenvectors to encode the node position information from graphs and then define edge channels to capture the global graph structures. The advantage of using the Laplacian eigenvector is that it can help the transformer model learn the proximity of neighbor nodes by maximizing the dot product operator between *Q* and *K* matrix. They first pre-computed Laplacian eigenvectors from the Laplacian matrix that could be calculated as:

(122) $$\begin{array}{c} \Delta =I-D^{-\frac{1}{2}}AD^{-\frac{1}{2}}=U^{⊺}\Lambda U\; \end{array}$$

where Δ is the Laplacian matrix, and Λ and *U* denote the eigenvalues and eigenvectors, respectively. The Laplacian eigenvectors $\lambda_{i}$ then could denote the positional encoding for node $v_{i}$. Given node $v_{i}$ with feature $x_{i}$ and the edge feature $e_{ij}$, the first hidden layer and edge channel could be defined as:

(123) $$\begin{array}{cc} \; & h_{i}^{\left(0\right)}=A^{0}x_{i}+\lambda_{i}^{\left(0\right)}+a^{\left(0\right)}\; \end{array}$$

(124) $$\begin{array}{cc} \; & e_{ij}^{\left(0\right)}=B^{\left(0\right)}e_{ij}+b^{\left(0\right)}\;.\; \end{array}$$

The hidden layers ${\hat{h}}_{i}^{\left(l+1\right)}$ of node $v_{i}$ and the edge channel ${\hat{e}}_{i}^{\left(l+1\right)}$ at layer l+1 could be defined as follows:

(125) $$\begin{array}{cc} \; & {\hat{h}}_{i}^{\left(l+1\right)}=O_{h}^{\left(l\right)}\underset{k=1}{\overset{H}{\left|\right|}}\;\left(\sum_{j\in N_{i}}A_{ij}^{k,l}V^{k,l}h_{j}^{l}\right), \end{array}$$

(126) $$\begin{array}{cc} \; & {\hat{e}}_{i}^{\left(l+1\right)}=O_{e}^{\left(l\right)}\underset{k=1}{\overset{H}{\left|\right|}}\;\left(A_{ij}^{k,l}\right), \end{array}$$

(127) $$\begin{array}{cc} \; & S_{ij}^{k,l}=\left(\frac{Q^{k,l}h_{i}^{l}\cdot K^{k,l}h_{j}^{l}}{\sqrt{d_{k}}}\right)\cdot E^{k,l}e_{ij}^{l}\; \end{array}$$

where *Q*, *K*, *V*, *E* are learned output projection matrices, *H* denotes the number of attention head.

Similar to [29], Kreuzer at al. [284] aimed to add edge channels to all pairs of nodes in an input graph. However, the critical difference between them is that they combine full-graph attention with sparse attention. One of the advantages of the model is that it could capture more global structural information since they implement self-attention to nodes in the sparse graph. Therefore, they use two different types of similarity matrices to guide the transformer model to distinguish the local and global connections between nodes in graphs. Formally, they re-define the similarity matrix for pair of connected and disconnected nodes, which could be defined as follows:

(128) $$\begin{array}{c} {\hat{S}}_{ij}^{k,l}=\left\{\begin{array}{cc} \frac{Q^{1,k,l}h_{i}^{l}\cdot K^{1,k,l}h_{i}^{l}E^{1,k,l}e_{ij}}{\sqrt{d}}\; & \mathrm{if}\;(v_{i},v_{j})\in E.\\ \frac{Q^{2,k,l}h_{i}^{l}\cdot K^{2,k,l}h_{i}^{l}E^{2,k,l}e_{ij}}{\sqrt{d}}\; & \mathrm{otherwise}. \end{array}\right.\; \end{array}$$

where ${\hat{S}}_{ij}^{k,l}$ denotes the similarity between two nodes $v_{i}$ and $v_{j}$, $\left(Q^{1},K^{1},E^{1}\right)$ and $\left(Q^{2},K^{2},E^{2}\right)$ are the keys, queries, and edge projections of connected and disconnected pair nodes, respectively.

In some specific cases where graphs are sparse, small, or fully connected, the self-attention mechanism could lead to the over-smoothing problem and structure loss since it cannot learn the graph structure. To overcome these limitations, several models adopt GNNs as an auxiliary model to maintain the local structure of the target nodes [99,100,285]. Rong et al. [100] proposed the Grover model, which integrates the message-passing mechanism into the transformer encoder for self-supervised tasks. They used the dynamic message-passing mechanism to capture the number of hops compatible with different graph structures. To avoid the over-smoothing problem, they used a long-range residual connection to strengthen the awareness of local structures.

Several models attempted to integrate GNNs on top of the multi-attention sublayers to preserve local structure between nodes neighbors [63,99,290]. For instance, Lin et al. [99] presented Mesh Graphormer model to capture the global and local information from 3D human mesh. Unlike the Grover model, they inserted a sublayer graph residual block with two GCN layers on top of the multi-head attention layer to capture more local connections between connected pair nodes. Hu et al. [285] integrated message-passing with a transformer model for heterogeneous graphs. Since heterogeneous graphs have different types of node and edge relations, they proposed an attention score, which could capture the importance of nodes. Given a source node $v_{i}$ and a target node $v_{j}$ with the edge $e_{ij}$, the attention score could be defined as:

(129) $$\begin{array}{cc} \; & S\left(v_{i},e_{ij},v_{j}\right)=\mathrm{Softmax}\left(\overset{M_{head}}{\underset{m=1}{\left|\right|}\;}\;\alpha^{m}\left(v_{i},e_{ij},v_{j}\right)\right)\; \end{array}$$

(130) $$\begin{array}{cc} \; & \alpha^{m}\left(v_{i},e_{ij},v_{j}\right)=K_{}^{m}\left(v_{i}\right)W_{\tau (e_{ij})}Q_{}^{m}\left(v_{j}\right)\frac{\mu }{\sqrt{d}}\; \end{array}$$

where $\alpha^{m}\left(\cdot ,\cdot ,\cdot \right)$ denotes the *m*-th attention head, $W_{\tau (e_{ij})}$ is the attentive trainable weights for each edge types, *K* and *Q* are linear projection of all type of source node $v_{i}$ and $v_{j}$, respectively, and μ is the importance of each relationship.

Nguyen et al. [61] introduced the UGformer model, which uses a convolution layer on top of the transformer layer to work with sparse and small graphs. Applying only self-attention could result in structure loss in several small-sized and sparse graphs. A GNN layer is stacked after the output of the transformer encoder to maintain local structures in graphs. One of the advantages of the GNN layer is that it can help the transformer model retain the local structure information since all the nodes in the input graph are fully connected.

In graphs, the nodes are arranged chaotically and non-ordered compared to sentences in documents and pixels in images. They can be in a multidimensional space and interact with each other through connection. Therefore, the structural information around a node can be extracted by the centrality of the node and its edges without the need for a positional encoding strategy. Recently, several proposed studies have shown remarkable results in understanding graph structure.

Several graph transformer models have been proposed to capture the structural relations in the natural language processing area. Zhu et al. [62] presented a transformer model to encode abstract meaning representation (AMR) graphs to word sequences. This is the first transformer model that aims to integrate structural knowledge in AMR graphs. The model aims to add a sequence of edge features to the similarity matrix and attention score to capture the graph structure. Formally, the attention score and the vector embedding could be defined as:

(131) $$\begin{array}{cc} \; & S_{ij}=\frac{\left(x_{i}W^{Q}\right){\left(x_{j}W^{K}+r_{ij}W^{R}\right)}^{⊺}}{\sqrt{d}}\; \end{array}$$

(132) $$\begin{array}{cc} \; & Z_{i}=\sum_{j=1}^{n}\mathrm{Softmax}\left(S_{ij}\right)\left(x_{j}W^{V}+r_{ij}W^{F}\right)\; \end{array}$$

where $W^{R}$ and $W^{F}$ are parameter matrices, $r_{ij}$ is the vector representation for the relation between $v_{i}$ and $v_{j}$, which could be computed by several methods, such as average values or summation. Khoo et al. [286] introduced the StA-PLAN model, which aims to detect fake news on social networking sites. Given a node $v_{i}$, the attention score and the node embedding could be defined as:

(133) $$\begin{array}{cc} \; & S_{ij}=\frac{q_{i}K_{j}^{⊺}+a_{ij}^{K}}{\sqrt{d}}\; \end{array}$$

(134) $$\begin{array}{cc} \; & Z_{i}=\sum_{j=1}^{n}\mathrm{Softmax}\left(S_{ij}\right)\left(Z_{j}+a_{ij}^{V}\right)\; \end{array}$$

where $a_{ij}^{K}$ and $a_{ij}^{V}$ denotes the learned parameter vectors, which represent the relation types between $v_{i}$ and $v_{j}$. The $a_{ij}^{K}$ matrix aims to capture the structural information surrounding target nodes, while the purpose of $a_{ij}^{V}$ matrix is to spread to other nodes.

The study from [66] aims to add the edge information between nodes to the similarity matrix. However, the difference is that they add a label information matrix combined with node features as input for the graph transformer. Formally, the feature propagation at the first layer could be defined as:

(135) $$\begin{array}{c} H^{\left(l+1\right)}=\sigma \left(\left(\left(1-\beta \right)\tilde{A}+\beta I\right)H^{\left(l\right)}\right)\;\;\;H^{\left(0\right)}=X+\hat{Y}W_{d}\; \end{array}$$

where *X* and $\hat{Y}$ denote the input feature and partially labeled matrix, respectively. $\tilde{A}=D^{-1}A$ and β is a predefined hyper-parameter. They then put a message-passing layer on top of the multi-head attention layers to capture the local graph structures.

Schmitt et al. [47] proposed a model that adds the relative position embedding parameter to the proximity and attention score matrices in the graph-to-text problem. The main objective of this model is to define the attention score of relationships between nodes based not only on the topology of the nodes but also on their connection weights extracted from shortest paths. Specifically, the proximity matrix of a node and its attention score can be defined as:

(136) $$\begin{array}{cc} \; & S_{ij}=\frac{H_{i}K_{}^{Q}\left(H_{j}W_{}^{K}\right)}{\sqrt{d}}+\gamma R_{ij}\; \end{array}$$

where γ denotes a scalar embedding, and $R_{ij}$ presents a relative positional encoding between node $v_{i}$ and $v_{j}$ which are sampled from shortest paths *P*.

Ying et al. [67] introduced the Graphormer model, which aims to encode effectively graph structures. The model first captures the importance of nodes in graphs by describing the node centrality.The hidden state at the first layer of a node $v_{i}$ could be defined as:

(137) $$\begin{array}{c} h_{i}^{\left(0\right)}=x_{i}+z_{\deg^{-}\left(v_{i}\right)}^{-}+z_{\deg^{+}\left(v_{i}\right)}^{+}\; \end{array}$$

where $z_{\deg^{-}\left(v_{i}\right)}^{-}$ and $z_{\deg^{+}\left(v_{i}\right)}^{+}$ depict the embedding vectors of in-degree and out-degree of node $v_{i}$, respectively. To capture the global structure and the connection between nodes, they add more information about the node pairwise and edge features to the similarity matrix *S*. Mathematically, the similarity matrix *S* that captures the relation between keys and queries matrix could be defined as:

(138) $$\begin{array}{cc} \; & S_{ij}=\frac{\left(h_{i}W_{Q}\right){\left(h_{j}W_{K}\right)}^{⊺}}{\sqrt{d}}+b_{\varphi (v_{i},v_{j})}+c_{ij}\; \end{array}$$

(139) $$\begin{array}{cc} \; & c_{ij}=\frac{1}{N}\sum_{n=1}^{N}x_{e_{n}}{\left(w_{n}^{E}\right)}^{⊺}\; \end{array}$$

where $b_{\varphi (v_{i},v_{j})}$ is the learnable scalar indexed by the shortest-path distance from node $v_{i}$ to node $v_{j}$, $\left(w_{n}^{E}\right)$ denotes the weight embedding of edge, and $x_{e_{n}}$ denotes the *n*-th edge feature in the shortest path from $v_{i}$ to $v_{j}$. Using the centrality encoding strategy, the Graphormer model could capture the importance of nodes in graphs that are significant in several graphs, such as the social network. Furthermore, the spatial encoding based on the shortest path could help the model capture the local and global structural information in graphs.

By contrast, Hussain et al. [30] proposed EGT (Edge-augmented Graph Transformer) model to capture the graph structure more in-depth by only using edge channels. The main idea of this model is to consider the proximity in an input graph matrix of size *k*-hop. In this case, the self-attention captures the edge information channels obtained using the shortest-path distance (SPD) between two nodes in an input matrix. They added edge channels to the proximity matrix of the two nodes and hidden layers for each target node. The attention matrix at layer *l*-th and *m*-th attention head could be defined as:

(140) $$\begin{array}{cc} \; & A^{m,l}=\mathrm{Softmax}\left(H_{}^{m,l}\right)⊙\sigma \left(G^{m,l}\right)\; \end{array}$$

(141) $$\begin{array}{cc} \; & H_{}^{m,l}=\left(\frac{Q^{m,l}{\left(K^{m,l}\right)}^{⊺}}{\sqrt{d_{k}}}\right)+E^{m,l}\; \end{array}$$

where *m*, *l* denote the *m*-th attention head and *l*-th hidden layer, respectively, $G^{m,l}$ and $E^{m,l}$ are the two matrices obtained from edge channels between two nodes by a linear function, $\sigma \left(\cdot \right)$ is the sigmoid function. To capture the importance of nodes, they introduced a centrality score for each node which could be obtained from a *k*-hop distance. The main idea is to make the model capable of distinguishing non-isomorphic subgraphs, and the model’s performance is at least better than the 1-WL test. Formally, the centrality scaler matrix could be defined as:

(142) $$\begin{array}{c} s_{i}^{m,l}=\ln\left(1+\sum_{j=1}^{N}\sigma \left(G^{m,l}e_{ij}\right)\right)\; \end{array}$$

where *N* denotes the number of nodes in a matrix input and $e_{ij}$ is the edge between two nodes $v_{i}$ and $v_{j}$. In addition, they also added positional encoding, which is based on SVD. They first decompose the adjacency matrix $A\approx \hat{U}{\hat{V}}^{⊺}$, then concatenate two matrices *U* and *V* as positional encoding. However, the experimental results show that the model’s performance is not significantly improved compared to the original version.

To sum up, since the graph structure differs from the text and images mentioned above, various models have adjusted self-attention to apply the transformer to graph data. Moreover, it can also be considered that graph transformer architecture is a GAT in fully connected graphs. Therefore, in some specially structured graphs, ideal models combining GNNs as an auxiliary module for transformers also yield remarkable results. Several models [30,47] showed remarkable success in an in-depth understanding of the graph structure using edge channels based on the shortest-path distance. These results could bring a new approach to applying transformer architecture to graph representation learning.

### 3.5. Non-Euclidean Models

Graph representation learning models in Euclidean space have shown significant results for various applications [4,291]. In Euclidean space, graph representation learning models aim to map the graph entities to low-dimensional vector points. However, in the real world, graphs could have complex structures and various shapes, and the number of nodes could increase exponentially over time [292]. Representing such graphs in Euclidean space could lead to an incomplete representation of the graph structure and information loss [68,293]. Several recent studies have focused on representing complex structural graphs in non-Euclidean space and different metrics, which yielded some desirable results [68,70,102,293]. Each type of geometry has the advantage of describing differently shaped graph structures. For graph representation in non-Euclidean spaces, there are two typical spaces, spherical and hyperbolic, each one has its advantages. Spherical space could represent graph structures with large cycles, while hyperbolic space is suitable for hierarchical graph structures. Another method is Gaussian-based models, which could learn embeddings as a probability distribution in a latent space. This can be appropriate with the distribution in several graphs since a node could belong to different clusters based on probability density. This section covers various models in non-Euclidean space and Gaussian models.

#### 3.5.1. Hyperbolic Embedding Models

Hyperbolic geometry has the advantage of representing hierarchical graph data, which is tree-like and mostly obeys the power law [292]. Since the Euclidean operators could not be implemented directly in hyperbolic space, most models focus on transforming the properties of models from hyperbolic space (e.g., operators, optimization) to a tangent space where we are familiar with Euclidean operators. We first briefly introduce some basic notions and definitions of hyperbolic geometry and then cover graph embedding models later.

**Definition** **9** (Hyperbolic space [102]). *A hyperbolic space (sometimes called Bolyai–Lobachevsky space) is an n-dimensional Riemannian manifold of constant negative curvature. When n=2, it is also called the hyperbolic plane.*

Due to the complex structure of hyperbolic space, the visual representation of data and implementing operators in hyperbolic space seems complicated. Most models use a tangent space to approximate a manifold as an *n*-dimensional vector space. Formally, the manifold and tangent space could be defined as follows:

**Definition** **10** (Manifold and Tangent space [293]). *A manifold M of multi-dimension n is a topological space where the Euclidean space $R^{n}$ could locally approximate its neighborhood. When n=2, it is also called surfaces. A tangent space $T_{v}M$ is a Euclidean space $R^{n}$ that approximates the manifold M at any node v in graphs.*

The hyperbolic space is a smooth Riemannian manifold, considered a locally Euclidean space where we could generate Euclidean operations. The Riemannian manifold could be defined as follows:

**Definition** **11** (Riemannian manifold [293]). *A Riemannian manifold is defined as a tuple $\left(M,g\right)$, where g denotes a Riemannian metric which is a smooth collection of inner products on the associated tangent space: ${\langle \cdot \;\cdot \rangle }_{v}:T_{v}M\times T_{v}M$. The metric space g denotes curvature properties, such as the angle and the volume.*

There are several isometric models which are different metrics. However, two hyperbolic models, Poincaré and Lorentz, are widely studied in graph representation learning. Mathematically, the Poincaré and Lorentz models could be defined as:

**Definition** **12** (Poincaré Model [70]). *A Poincaré ball is a Riemannian manifold with a tuple $\left(B_{c}^{n},g_{x}^{B}\right)$, where c is a negative curvature, and $B_{c}^{n}=\left\{x\in R^{n}:{∥x∥}^{2}<-\frac{1}{c}\right\}$ is a open ball with radius $r=1/\sqrt{\left|c\right|}$. The matrix tensor $g_{x}^{B}={\left(\lambda_{x}^{2}\right)}^{2}g_{}^{E}$ denotes a conformal factor $\lambda_{x}^{c}=\frac{2}{1+c{∥x∥}_{2}^{2}}$ and $g_{}^{E}$ is a Euclidean matrix. Since $R^{2}$ could present a single hierarchical structure sufficiently, the Poincaré disk $B_{2}^{n}$ is commonly used to define hyperbolic geometry.*

Unlike the Poincaré disk, the Lorentz model is suitable for representing cyclic graphs. The Lorentz model has different characteristics from the Poincaré disk, but they are equivalent and could be transformed into each other. Mathematically, the Lorentz model is defined as follows:

**Definition** **13** (Lorentz/hyperboloid Model [102]). *A Lorentz or hyperboloid model is a Riemannian manifold with a tuple $\left(L_{c}^{n},g_{x}^{L}\right)$, where $L_{c}^{n}=\left\{x\in R^{n+1}:{\langle x,x\rangle }_{L}<\frac{1}{c}\right\}$ with a negative curvature c, and $g_{c}^{n}=diag{\left(\left[-1\;1\;1\;\cdots \;1\right]\right)}_{n}$.*

Most studies flatten a hyperbolic manifold and then apply graph operations in tangent space, which are similar to Euclidean space. Once the results are available, they will be mapped back into the hyperbolic space. The projection of components from hyperbolic space to the manifold and back projection is handed through exponential and logarithmic mapping functions, which will be shown in the models below in detail. Table 16 summarizes hyperbolic models for graphs.

Nickel Kiela [70] was among the first studies to learn graph embeddings in Poincaré ball based on similarities and hierarchies of nodes. They first put all nodes in graphs into the Poincaré disk and optimize the distance between pairwise nodes. Mathematically, the distance measure in Poincaré disk between two nodes $v_{i}$ and $v_{j}$ could be defined as:

(143) $$\begin{array}{c} d\left(Z_{i},Z_{j}\right)=\mathrm{arccosh}\left(1+2\frac{{∥Z_{i}-Z_{j}∥}^{2}}{\left(1-{∥Z_{i}∥}^{2}\right)\left(1-{∥Z_{j}∥}^{2}\right)}\right)\;. \end{array}$$

They then define operators and compute the loss function on the tangent space. The loss function can be minimized using Riemannian SGD (RSGD) optimization. Formally, the loss function is defined as:

(144) $$\begin{array}{c} L\left(V\right)=\sum_{(v_{i},v_{j})\in E}\log\frac{e^{-d(v_{i},v_{j})}}{\sum_{v_{k}\in N_{neg}\left(v_{i}\right)}e^{-d(v_{i},v_{k})}}\;. \end{array}$$

Similarly, the study of Nickel and Kiela [102] tried to improve embeddings in the Poincaré model by learning pairwise hierarchical relations in graphs. However, the difference between [70,102] is that they adopted the Lorentz model to learn embeddings. Wang et al. [71] tried to learn embeddings of heterogeneous graphs in the Poincaré disk. The meta-paths are generated using random-walk sampling strategies. They then use Equation (143) to calculate the distance between two nodes in the vector space. The Riemannian stochastic gradient descent (RSGD) is used to optimize the objective function, which minimizes the proximity between target nodes and their neighbors. Mathematically, given a node $v_{i}$ and set of its neighbors $N(v_{i})$, the objective function could be defined as:

(145) $$\begin{array}{c} L\left(V\right)=\sum_{(v_{i},v_{j})\in E}\left[\alpha \log\sigma \left(Z_{i}^{⊺}Z_{j}\right)+\left(1-\alpha \right)\sum_{k=1}^{n}\left(E_{v_{k}∼P\left(v_{i}\right)}\log\sigma \left(Z_{i}^{⊺}Z_{k}\right)\right)\right]\;. \end{array}$$

Since there is no definition of GNN operations in the hyperbolic space, most models tried to transform GNN operators from the hyperbolic space to the tangent manifold and performed the operators in this space. The work of Chami et al. [293] aimed to transform features from the Euclidean space to a tangent manifold and perform aggregation and activation functions on this space. The results are then projected to the *H* space. Exponential and logarithmic functions are used to map between *T* and *H* space. Given a vector $x^{0,E}\in R^{d}$ in Euclidean space, the mapping features from Euclidean space into hyperboloid manifold could be defined as:

(146) $$\begin{array}{c} x^{0,H}=\exp_{o}^{C}\left(0,x^{0,E}\right)=\left(\sqrt{C}\cosh\left(\frac{\left|\right|x^{0,E}|{|}_{2}}{\sqrt{K}}\right),\sqrt{C}\sinh\left(\frac{\left|\right|x^{0,E}|{|}_{2}}{\sqrt{K}}\right)\frac{x^{0,E}}{\left|\right|x^{0,E}|{|}_{2}}\right)\; \end{array}$$

where $o:=\left\{\sqrt{C},0,\cdots ,0\right\}\in H^{d,C}$ denotes the original pole in the hyperbolic space. The model defines trainable curves *C* at different layers and mapping operations between the hyperbolic space and manifold. After mapping input features into hyperbolic space, the definition operators for the message mapping mechanism can be defined as:

(147) $$\begin{array}{cc} \; & h_{i}^{l,H}=\left(W^{l}{⊗}^{K_{l-1}}x_{i}^{l-1,H}\right){⊕}^{C_{l-1}}b^{l}\; \end{array}$$

(148) $$\begin{array}{cc} \; & m_{i}^{l,H}={\mathrm{AGG}}^{C_{l-1}}{\left(h_{}^{l,H}\right)}_{i}\; \end{array}$$

(149) $$\begin{array}{cc} \; & Z_{i}^{l,H}=\sigma^{{⊗}^{C_{l-1},C_{l}}}\left(m_{i}^{l,H}\right)\; \end{array}$$

where AGG(·) denotes the hyperbolic aggregation, which is based on the attention mechanism and could be calculated as:

(150) $$\begin{array}{c} {\mathrm{AGG}}^{C}{\left(x_{}^{H}\right)}_{i}=\exp_{x_{i}^{H}}^{C}\left(\sum_{v_{j}\in N\left(v_{i}\right)}w{}_{ij}\log_{x_{i}^{H}}^{C}x_{j}^{H}\right)\;. \end{array}$$

Similarly, Zhang et al. [68] used Gyrovector space to build GNN layers in hyperbolic space. The Gyrovector space is an open *d*-dimensional ball which could be defined as:

(151) $$\begin{array}{c} D_{c}^{d}:=\left\{x\in R^{d}:c{∥x∥}^{2}<1\right\}\; \end{array}$$

where *c* denotes the radius of the ball. They first put input features *x* from Euclidean space into the Gyrovector ball by an exponential mapping:

(152) $$\begin{array}{c} x^{H}=\exp_{o}^{c}\left(x\right)=\tanh\left(\sqrt{c}∥x∥\right)\frac{x}{\left(\sqrt{c}∥x∥\right)}\;. \end{array}$$

After exponential mapping, a linear transform is used as a latent representation of each node. Formally, the hidden state of a node is obtained by applying a shared linear transformation matrix *M*:

(153) $$\begin{array}{c} h_{i}=M{⊗}^{c}x^{H}=\frac{1}{\sqrt{c}}\tanh\left(\frac{∥Mx^{H}∥}{∥x^{H}∥}\tanh^{-1}\left(\sqrt{c}∥x∥\right)\right)\;. \end{array}$$

Liu et al. [294] employed a similar approach for HGNNs. However, the main objective of this study aimed to compare which space could be suitable for graph data representation between Poincaré disk and Lorentz space in terms of implementing GNN models. Zhang et al. [69] proposed an LGCN model to learn embeddings on the Lorentzian model. They first map input features from Euclidean space to hyperbolic space and rebuild GNN operators, such as dot product and linear transformation. In addition, they aggregate information from neighborhood nodes by computing the centroid of nodes in the hyperbolic space. Given a node $v_{i}$ and its feature $h_{i}^{d,C}\in H^{d\times C}$ and a set of its neighbors $N(v_{i})$, finding a centroid of nodes could be considered as an optimization problem:

(154) $$\begin{array}{cc} \; & {\bar{c}}^{d,C}=\underset{c^{d,C}\in H^{d,C}}{arg min}\;\sum_{j\in N(v_{i})}w{}_{ij}d_{L}^{2}\left(h_{j}^{d,C},c^{d,C}\right)\; \end{array}$$

(155) $$\begin{array}{cc} \; & c^{d,C}=\sqrt{C}\frac{\sum_{j\in N(v_{i})}w{}_{ij}h_{j}^{d,C}}{\left|{∥\sum_{j\in N(v_{i})}w{}_{ij}h_{j}^{d,C}∥}_{L}\right|}\; \end{array}$$

where $w_{ij}$ denotes the weights that could be normalized and computed via an attention coefficient μ as:

(156) $$\begin{array}{cc} \; & w_{ij}=\frac{\exp(\mu {}_{ij})}{\sum_{v_{m}\in N\left(v_{i}\right)}\exp(\mu {}_{im})}\; \end{array}$$

(157) $$\begin{array}{cc} \; & \mu {}_{ij}=-d_{L}^{2}\left(M{⊗}^{C}h_{i}^{d,C},M{⊗}^{C}h_{j}^{d,C}\right)\; \end{array}$$

where $d_{L}^{2}$ denotes a squared Lorentzian distance [295], *M* is a matrix to transform node feature to attention-based space.

#### 3.5.2. Spherical Embedding Models

Spherical geometry is a topological space that could represent graph structure with large cycles [296]. A spherical space is an *n*-dimensional Riemannian manifold of constant positive curvature (c>0). The implementation of operators is similar to hyperbolic space. For each point *x* in the spherical space *S*, the connection between the spherical space *S* and a tangent space $T_{x}S_{c}^{n}$ could be computed through exponential and logarithmic mapping, which could be defined as:

(158) $$\begin{array}{cc} \; & \exp_{x}^{c}\left(v\right)=x{⊕}^{c}\left(\tanh\left(\sqrt{c}\frac{\lambda_{x}∥v∥}{2}\right)\frac{v}{\sqrt{c}∥v∥}\right)\; \end{array}$$

(159) $$\begin{array}{cc} \; & \log_{x}^{c}\left(y\right)=\frac{2}{\sqrt{c}\lambda_{x}}\tanh^{-1}\left(\sqrt{c}∥-x{⊕}^{c}y∥\right)\frac{-x{⊕}^{c}y}{∥-x{⊕}^{c}y∥}\; \end{array}$$

where *x* and *y* are two points in the *S* space and $v\in T_{x}S_{c}^{n}$. The distance between *x* and *y*, and the operator ${⊕}_{c}$ is the Möbius addition for any x,y∈S which could be defined as:

(160) $$\begin{array}{cc} d_{c}\left(x,y\right) & =\frac{2}{\sqrt{c}}\tanh^{-1}\left(\sqrt{c}∥-x{⊕}^{c}y∥\right)\; \end{array}$$

(161) $$\begin{array}{cc} x{⊕}^{c}y & =\frac{\left(1+2c\langle x,y\rangle +c{∥y∥}^{2}\right)x+\left(1-c{∥x∥}^{2}\right)y}{1+2c\langle x,y\rangle +c^{2}{∥x∥}^{2}{∥y∥}^{2}}\; \end{array}$$

A few studies on spherical space have yielded promising results in recent years [103,297]. For instance, Cao et al. [103] proposed combining the representation of the knowledge graphs into three different spaces, including Euclidean, hyperbolic, and spherical spaces. Specifically, each entity *e* of the knowledge graph could be presented by three embeddings: Euclidean space $E_{e}$, hyperbolic space $E_{h}$, and hypersphere space $E_{s}$. For a triplet (h,r,t) denotes the head, relation, and tail, respectively, in the knowledge graph, the embedding of an entity *e* in the hyperbolic and hypersphere space could be defined as:

(162) $$\begin{array}{cc} H_{e} & =r{⊗}^{v}\exp_{o}^{v}\left(e\right)\; \end{array}$$

(163) $$\begin{array}{cc} S_{e} & =r{⊗}^{u}\exp_{o}^{u}\left(e\right)\; \end{array}$$

where $H_{e}$ and $S_{e}$ denote the embedding of entity *e* in the hyperbolic and hypersphere space with two negative and positive curvatures *u* and *v*, respectively. They then can obtain the embedding for each entity by combining embedding components from different spaces through the exponential function.

#### 3.5.3. Gaussian Embedding Models

Most of the aforementioned graph embedding models represent graph entities as vector points in latent space. However, several models proposed using probability distributions to learn embeddings, considering each entity as density-based embedding. Unlike vector-point embedding models, density-based models learn embeddings as continuous density in latent space. Vector embeddings could be represented as a multivariate Gaussian distribution $P∼N\left(\mu ,\Sigma \right)$. Table 17 presents a summary of Gaussian embedding models for various types of graphs.

Most Gaussian embedding models are inspired by the Word2Gauss approach [300] in natural language processing. Each word is projected into an infinite-dimensional space rather than a vector which could enable a rich geometry for better quantification of the word-type properties in the latent space. Kipf and Welling [72] introduced a VGAE (Variational Graph Autoencoder) model based on an autoencoder architecture. The encoder part includes two convolutional graph layers. The model takes an adjacency matrix *A* and features *X* as input for GCNs layers. Mathematically, the μ and $\log\Sigma^{2}$ parameters can be defined as:

(164) $$\begin{array}{cc} \mu & =\sigma_{\mu }\left(H^{0},A\right)=\tilde{A}H^{0}W_{1}\; \end{array}$$

(165) $$\begin{array}{cc} \log\Sigma^{2} & =\sigma_{\Sigma }\left(H^{0},A\right)=\tilde{A}H^{0}W_{1}\; \end{array}$$

The vector embedding $Z_{i}$ for each node $v_{i}$ could be defined as:

(166) $$\begin{array}{c} q\left(Z_{i}|X,A\right)=N\left(Z_{i}|\mu_{i},diag\left(\Sigma_{i}^{2}\right)\right)\;. \end{array}$$

Zhu et al. [298] proposed DVNE (Deep Variational Network Embedding) model to preserve the similarity between the distributions based on autoencoder architecture. The DVNE model aims to preserve 1st-order and 2nd-order proximity in Wasserstein space. The main objective is to minimize the Wasserstein distance between distributions over the Gaussian distribution. For $p\in \left[0,\infty \right)$, the Wasserstein *p*-distance between two distributions *P* and *Q* could be defined as:

(167) $$\begin{array}{c} d_{p}\left(P,Q\right):=\underset{}{\underset{x,y}{\inf}\;{∥x-y∥}_{p}}\; \end{array}$$

where (x,y) is all pairs of random variables. Since Gaussian distribution is used to present the uncertainty of nodes in latent space, they aim to preserve the Wasserstein distance, which could be formulated as:

(168) $$\begin{array}{c} W_{2}{\left(N\left(\mu_{1},\Sigma_{1}\right);N\left(\mu_{2},\Sigma_{2}\right)\right)}^{2}={∥\mu_{1}-\mu_{2}∥}_{2}^{2}+{∥\Sigma_{1}-\Sigma_{2}∥}_{F}^{2}\; \end{array}$$

where $\Sigma_{1}$ and $\Sigma_{2}$ are diagonal covariance matrices. They use the square-exponential loss to minimize the proximity and the reconstruction loss that could be defined as:

(169) $$\begin{array}{cc} \; & L\left(V\right)=L_{1}\left(V\right)+\alpha L_{2}\left(V\right)\; \end{array}$$

(170) $$\begin{array}{cc} \; & L_{1}\left(V\right)=\sum_{v_{i},v_{j},v_{k}\in V}E_{ij}^{2}+\exp\left(-E_{ik}\right)\; \end{array}$$

(171) $$\begin{array}{cc} \; & L_{2}\left(V\right)=\underset{X,\hat{X}}{inf}\;{∥X\circ \left(X-\hat{X}\right)∥}_{2}^{2}\; \end{array}$$

where (i,j,k) denotes a tuple $\left(v_{i},v_{j},v_{k}\right)$ from *k*-hop neighborhood of $v_{i}$ with constraints defined in Equation (174).

Santos et al. [104] targeted node representation associated with the uncertainty in classification tasks for heterogeneous graphs. Specifically, each node $v_{i}$ is projected into latent space, which is followed by a Gaussian distribution $Z_{i}=N\left(\mu_{i},\Sigma_{i}\right)$. The key objective of the model is to minimize the loss function for the classification problem and regularization for structural loss using stochastic gradient descent. In terms of structure preservation, they aim to preserve the 1-hop distance for each target node in graphs. They use KL Divergence to minimize the difference between two probability distributions which could be defined as:

(172) $$\begin{array}{cc} \; & L\left(V\right)=\sum_{v_{i}\in V}\sum_{v_{j}\in N\left(v_{i}\right)}w_{ij}D_{KL}\left(Z_{i}\left|\right|Z_{j}\right), \end{array}$$

(173) $$\begin{array}{cc} \; & D_{KL}\left(Z_{i}\left|\right|Z_{j}\right)=\frac{1}{2}\left(tr\left(\Sigma_{i}^{-1}\Sigma_{j}^{}\right)+{\left(\mu_{i}-\mu_{j}\right)}^{⊺}\Sigma_{i}^{-1}\left(\mu_{i}-\mu_{j}\right)-d-\log\frac{\det\left(\Sigma_{j}^{}\right)}{\det\left(\Sigma_{i}^{}\right)}\right)\; \end{array}$$

where $w_{ij}$ denotes the weight of $e_{ij}$.

Similar to [104], Bojchevski and Gunnemann [23] proposed a G2G (Graph2Gauss) model, an idea of learning node embeddings as uncertain. The difference between G2G and [104] is that the G2G model could preserve up to *k*-hop neighborhood proximity, which captures the global graph structure. Given a target node $v_{i}$ and set of its neighbors within *k*-hop distance $N_{ik}$, the objective of G2G is to build a set of constraints that the dissimilarity measure from node $v_{i}$ to all nodes in $N_{i1}$ should be smaller compared to all nodes in $N_{i2}$ and so on, up to *k*-hop. Mathematically, the pairwise constraints could be defined as:

(174) $$\begin{array}{c} E\left(Z_{i},Z_{j}\right)<E\left(Z_{i},Z_{m}\right),\forall v_{i}\in V,\forall v_{m}\in N_{ik},\forall j<m\;. \end{array}$$

Similar to [104], Equation (173) is used to measure the dissimilarity between two distributions, and they adopt square-exponential loss for optimization. Since there has been an uncertain lack of information about node embedding in latent space, the G2G model could learn node embeddings efficiently by representing nodes as Gaussian distribution. In addition, the personalized ranking could learn the order of nodes in graphs and the distance between them, eventually capturing local and global structural information.

To learn embeddings in knowledge graphs, He et al. [299] proposed the KG2E model to learn the certainty of entities and relations in knowledge graphs. This first study aims to learn node embeddings based on density in knowledge graphs. Furthermore, KG2E adopted two methods to measure the scores of triplets to learn embeddings based on symmetric and asymmetric similarity. For each triplet (h,r,t) which denotes head, relation, and tail, respectively, there are three different Gaussian distributions $H∼N\left(\mu_{h},\Sigma_{h}\right)$, $R∼N\left(\mu_{r},\Sigma_{r}\right)$,$T∼N\left(\mu_{t},\Sigma_{t}\right)$.

The score function of the KG2E model could be defined as:

(175) $$\begin{array}{c} s\left(h,r,t\right)=s\left(P_{e},P_{r}\right)=D_{KL}\left(P_{e},P_{r}\right)\; \end{array}$$

where $P_{e}$ denotes probability distribution $P_{e}∼N\left(\mu_{h}-\mu_{t},\Sigma_{h}-\Sigma_{t}\right)$, and $D_{KL}\left(\cdot \;\cdot \right)$ is defined in Equation (173).

## 4. Applications

This section focuses on practical applications of graph representation learning in various fields. We first explain how a graph can be constructed in different contexts and then discuss how graph-based models could be applied in practice. In several areas, graph embedding models may not be applied directly to solve specific tasks in the real world. However, they could act as auxiliary modules to help improve the performance of specific tasks.

### 4.1. Computer Vision

In image processing, a graph could be constructed for image processing problems by representing each pixel as a node and each edge describing the relationship between nodes. Several CGNNs have been proposed for the task of learning convolutional filters in the frequency domain [56,96,301,302] for classification tasks. For instance, Defferrard et al. [96] transform images from the spatial domain to the spectral domain using a Fourier transform. They then learn the convolution filter on the frequency domain to produce a sparse Laplacian matrix as input for classification tasks.

Each image segment or an entire image could be considered to be nodes and edges describing the relationships between them. Several graph-based methods adopt this strategy for the clustering tasks [303,304]. For example, Yang et al. [304] first extract image features from a CNN model and then build a large face image dataset. By considering *k* nearest neighbors as super nodes and the relationship between them as edges, they could construct graphs and use CGNNs to learn the cluster labels.

By considering each object in images as nodes and the relations between them as edges, several GNNs are applied to learn the proximity between the objects [305,306]. The graph embedding models can help image processing algorithms understand images’ semantic relationships and spatial structure more deeply. CGNNs could aid in connecting relationships between objects in images and scene graphs [307,308]. For example, Johnson et al. [308] used scene graphs to predict corresponding layouts by calculating embeddings for objects and their relationships in the image. The model is used to learn the vector embeddings for objects. CGNNs could also help build a reasoning network for objects in images to capture the interaction between objects [309,310]. Chen et al. [309] proposed CGNNs for relation reasoning for new actions, which should be more friendly in interaction space. CGNNs with a self-attention mechanism could help enhance the object representation in images combined with text guidance [310]. This strategy could capture relations between arbitrary regions in images and the interactions between objects in images.

Graphs are also constructed by combining general knowledge of text and images with image-question-facts. Specifically, each node in the graph is an embedding processed from the word and image processing algorithms, and edges represent the relationship between them. CGNNs are used to learn embeddings to retrieve the correct fact. For instance, Cui et al. [306] build a joint model by combining the semantic and spatial scene graphs to find internal correlations across object instances in images. They first use object detection approaches to detect objects in the images. Then, a semantic graph is constructed with nodes as objects, and edges connect objects in the image.

The sequence of skeletons is treated as a dynamic graph consisting of a sequence of snapshots. Each snapshot corresponds to a skeleton frame where each node is a joint, and the edge describes the connection of bones. Several graph-based models effectively learn features containing joint and bone information and their dependencies, which can facilitate action recognition. For instance, spatial and motion information in skeleton data could be presented in graphs for pose prediction [311]. CGNNs could also help to understand and recognize action sequences in videos and object relationships [312,313,314]. The models could assign candidate moments by structural reasoning to model relations between moments in videos. Each moment could be considered to be a node, and the edges are relations between them.

### 4.2. Natural Language Processing

A graph can be built by considering each word/document as a node, and edges could describe the relationship between the nodes or their occurrence frequency in a given context. Recently, graph-based models, which are mainly based on GNNs have attracted much attention in several applications to text classification tasks [16,18,22,211]. These models can capture the rich relational structure and preserve global structure information of documents. For instance, the DGCN model [211] was proposed to classify scientific publications by considering each paper as a node and edges as reference citations. Hamilton et al. [22] build document graphs from Reddit post data and citation data to predict paper and post categories.

Each sentence could be represented as a graph, with each node being a word and an edge describing the dependency between them. Recently, the graph-based models applied in machine translation show the potential of syntax-aware feature representations of words [315,316]. For instance, CGNNs could be used to predict syntactic dependency trees of source sentences to produce representations of words [315,316]. Bastings et al. [315] first transformed sentences into syntactic dependency trees. They then use convolution layers to learn dependency relation types which could support language models to understand the meanings of words in depth. SynGCN [317] could capture the structural relation between words in sentences from a dependency graph. They consider nodes as words and edges as the co-occurrence frequencies of two words in the entire corpus. The structural semantics could then be used to improve the performance of the Elmo model. F-GCN model (fusion GCN) from [318] can help a dialog system deal with diagram questions. They then use RNNs to capture the meaning of answers by considering the answer representation obtained from F-GCN as inputs.

### 4.3. Computer Security

The development of technology has increased the cyber security risk that is a social concern. Researchers have proposed various solutions, such as firewalls and intrusion detection systems against network attacks. The intrusion detection system could be divided into two main approaches: predefined rule-based and artificial intelligence-based. In recent years, several GNNs have also been applied to improve the detection of network attacks [231,319].

A graph network is constructed by nodes that are IP addresses and edges that are packet data flows exchanged between IP addresses. Hao et al. [319] proposed a Packet2Vec model to capture the proximity features based on graph representation to build an intrusion detection system. They consider each network traffic flow as a graph where nodes are packets and edges denote the similarity between two packets. They then prune the relational graph to obtain a local proximity feature for each graph and use this as input for an autoencoder that could learn embeddings for each network flow. By contrast, Lo et al. [231] built an intrusion detection system by improving the GraphSAGE model for building intrusion detection systems. They construct a computer graph by considering each IP address as a node and the edges as links between IP addresses. By constructing the computer network graph, they can train the model with packet information from clients to the server to detect anomalous information.

Since the source code can be represented as an abstract syntax tree, several graph embedding models have been proposed to help detect malware code by learning dependency graphs. The dependency graph is built with API function nodes and directed edges representing other functional queries from the current function [320,321]. For instance, Narayanan et al. [320] built rooted subgraphs that capture the connection between API functions in source code. The model learns latent representations of rooted subgraphs and detects malware code in an Android operating system.

### 4.4. Bioinformatics

Drug discovery is vital in finding new chemical properties to treat diseases. A graph could represent the interaction between drug–drug, drug–target, and protein–protein by considering each node as a drug or a protein, and the edges describe the interaction between them. Since searching for successful drug candidates is challenging, graph-based models can aid experiments in the chemistry area. Several models [322,323,324] use a matrix-factorization-based model to predict the interaction between the clinical manifestations of diseases and their molecular signatures. This could contribute to predicting potential diseases based on human genomic databases. Yoshihiro et al. [325] constructed a bipartite graph as a chemical and genomic space to capture the interaction between drug and protein nodes. The matrix factorization-based model is used to learn embeddings and detect potential drug interactions [326,327]. The matrix factorization-based model is also used to project drugs and targets into a common low-rank feature space and create new drugs and targets for predicting drug–target interactions [328,329,330].

For protein–protein interaction presentation, the atoms could be considered to be nodes and edges are bonds that link two atoms. CGNNs help to predict the properties of molecular and classification tasks [323,324]. The attention-based CGNN model could predict chemical stability [331]. For identifying drug targets, several CGNNs are used to present the structure of protein–protein interaction assessment and function prediction [332]. The DeepWalk model measures similarities within a miRNA-disease association network [333].

In recent years, various GNN-based models have been proposed to predict drug–drug interactions [334,335,336,337]. A knowledge graph is constructed by a set of entity-relation-entity triples that describe the interactions between drug–drug nodes. Most knowledge graphs comprise drug features gained from DrugBank or KEGG dataset. GNN-based models could then explore the topological structure of drugs in the knowledge graph to predict the potential drug–drug interactions. For example, Lin et al. [337] proposed a GNN model to learn drug features and knowledge graph structure to predict the drug–drug interaction. Su et al. [334] proposed a DDKG model based on attentive GNNs to learn the drug embedding. The key idea of this model is first to initialize the node features based on SMILE sequences gained from a random-walk sampling strategy. This could construct the node features, bringing a global structure at the initial step. The model then learns node embeddings based on attention from the neighborhood and triple facts.

For drug–target interaction, which is a crucial area in drug discovery, several graph-based models could help to predict the drug–target interactions [325,338,339,340]. For example, Hao et al. [338] proposed a GNN-based model to learn drug–target interaction. A heterogeneous graph is constructed by nodes denoting a drug–target pair, and the edges describe the connection strength between the pairs. The model then applies the graph convolution filter to learn the feature of drug–protein pairs. Peng et al. [340] introduced EEG-DTI (end-to-end graph drug–target interactions) model to predict the relations between drugs and targets based on the GCN model. A heterogeneous graph represents the interactions between drugs and targets (e.g., drug–drug interaction and drug–protein interaction). Each edge type denotes the interactions between two entities in the heterogeneous graph, computed based on Jaccard similarity. GCN model then could help to learn node representation and predict the drug–target relation.

### 4.5. Social Media Analysis

Social networks have played an essential role in communication among users worldwide. Various graph embedding models have been applied to social media to learn embeddings [72,216]. In social networks, most graphs are initialized by defining nodes as users and edges describing user relationships (e.g., messages). Several GNNs are applied to help detect fake news shared on social networking platforms [341,342]. Nguyen et al. [343] employed GraphSAGE to classify fake news in social media.

For social interaction network representation, directed graphs can be built with nodes as users and edges describing user social relationships or action interactions [344,345]. GAT model [346] is used to predict the influence of essential users in the social network. Piao et al. [344] proposed a motif-based graph attention network to predict the social relationships between customers and companies. CGNNs [345] could classify relations between political and regular news media users.

### 4.6. Recommendation Systems

Bipartite graphs could be used to represent user–item interactions in recommendation systems. In the graph, nodes can be presented as users and categories, and directed edges denote interactions between users and items. Several traditional models based on matrix factorization have been applied to help the system understand the predictions of users’ ratings on items or click actions [347,348].

The side information is mainly the attributes of categories and users. This information helps to represent the relationship between users and items [349,350]. Heterogeneous graphs with properties of nodes and relationship types have been proposed to represent side information. Several shallow models [351,352] and GNNs [353,354] have been proposed to capture the interaction between users and items with side information.

Knowledge graphs can represent entities and their relationships from the knowledge base. Knowledge graphs, therefore, can collect high-order proximity between items and user interactions [267,355]. Exploiting social correlations such as homophily and social influence can improve the performance of online recommendation systems. Several applications put user and item interaction into CGNNs to learn embeddings and solve collaborative filtering problems [356,357].

### 4.7. Smart Cities

People encounter current traffic-related issues in big cities, such as traffic jams and difficulty finding parking spaces. Addressing these issues that play an essential role in building smart cities and transportation has been studied in the literature. Traffic forecasting is one of the crucial factors in improving traffic efficiency and solving related problems.

In this context, a graph can be considered a whole city map with nodes as intersections and edges describing paths connecting the nodes [358]. For the traffic prediction problem, nodes and edges can have properties that describe the traffic state. Besides static graphs, dynamic graphs with dynamic adjacency matrices are also used to describe the dynamic state of overtime traffic. In recent years, GNNs have been widely applied to predict traffic conditions [359,360,361]. CGNNs are applied to predict traffic flow conditions in big cities. For example, the attention-based GNNs are applied to predict traffic congestion status [359]. The self-attention mechanism can capture the state around the target vehicles by considering connections to its ego network.

Dynamic graphs can represent a spatial-temporal dependency. Several applications are also practical to spatial-temporal transportation networks [358,360,361] to predict traffic flow. A study from [360] applied CGNNs to capture the traffic’s current state and historical conditions to predict the next state of the traffic condition. They construct a dynamic graph including a collection of snapshots, and each snapshot is the current state of the traffic. The model then uses temporal convolution layers to learn dynamic node features.

There are several applications of graph embedding for energy-related problems, such as predicting electricity consumption and predicting wind and solar energy through IoT systems [362,363,364]. For example, in the problem of solar irradiance forecasting, a graph can be presented with nodes being the locations of energy measurements and edges describing the correlation between them according to historical data. By contrast, with wind speed forecasting systems, nodes describe wind farms, and edges represent two nodes as neighbors. For instance, a convolutional graph autoencoder-based model is used to help predict the radiative state of solar energy [362]. Khodayar et al. [363] presented a CGNN model to predict wind speed and direction.

### 4.8. Computational Social Science

The analysis of social issues and human behavior has been expanded due to the increased availability of big data. The application of computational science has created new opportunities for researchers in social science to achieve more detailed information by examining the trends and patterns of social phenomena.

Graph-based models provide an improved understanding of social issues, ranging from social inequity to the spread of child maltreatment across generations, using data, theory, and diverse media sources. In existing studies, directed acyclic graphs are typically used to represent the research hypotheses about causal relationships among variables based on existing literature [365]. They encode nodes in DAG graphs using color and predicting factors affecting children’s psychology.

The graph-based models have also been applied to political problems to explore the phenomena and trends of influence of political populations in social networks [366,367,368]. For example, the Community2Vec model [369] is used in [366] to identify political populations in a community. They measure the similarity between politically different communities and identify changes and trends in the community.

### 4.9. Digital Humanity

There is a growing interest in computational narrative analysis in the field of digital humanities. A character graph is one of the essential ways of expressing narratives, representing various relationships formed between characters as the story progresses. There are various methods of constructing a character graph. Typically, they use conversations in the story [370,371], consider events that make up the story [372,373], or are based on the co-occurrence of characters [374,375]. Recently, high-quality distributed representations of characters have been attempted for efficient and easy machine learning of character graphs. Lee and Jung [168] applied a subgraph-based graph embedding model to the dynamic networks of movie characters to compare similarities between stories. Inoue et al. [376] presented GNNs that could help to learn character embedding. If the characters in different works share similar properties, their connection relationships can be represented. Kounelis et al. [377] presented the movie’s plot to improve the movie recommendation system’s performance using the Graph2Vec model. First, a character relationship graph containing all necessary information for plot representation was built using the movie script. Graph embedding was then generated from the character relationship graph through the embedding method.

Since the digitization of large-scale literature works enables computer analysis of narratives, character graph embedding can be used in various ways in digital humanities. First, it is easy to measure similarities between stories. Second, since the unique aesthetic characteristics of a specific writer can be identified through machine learning on character graph embedding, it can be used to compare the styles of writers or to develop a story generation system that imitates the writing style of a specific writer. Third, characters can be classified based on their roles and personalities through character graph embedding. Fourth, character graph embedding can play an essential role in improving the computer’s narrative understanding in research on the narrative intelligence of computers, which has been attracting significant interest in recent years. Riedl [378] defined narrative intelligence as the ability to create and understand stories and argued that when computers are equipped with narrative intelligence, systems benefit humans, such as human-computer dialog systems can be developed.

### 4.10. Semiconductor Manufacturing

Recently, graph representation learning models have expanded their field of applications to semiconductor research and development, including semiconductor material screening [379], circuit design [380,381], chip design [382], and semiconductor manufacturing and supply chain management [383,384]. A graph could be constructed from crystal networks with nodes being atoms and edges describing the relation between them. GNNs could help to predict material properties for the fast screening of candidate materials. A tuples graph neural network exhibits an improved generalization capability for unseen data for bandgap prediction in perovskite crystals, 2D material, materials for solar cells, and binary and ternary inorganic compound semiconductors [379].

For circuit [380,381] (or chip [382]) design tasks, a graph could be constructed with nodes being transistors (or macro-cells/blocks) and edges being wires (or routings). A computer chip could be considered to be a hypergraph of circuit components as a netlist graph. Chip designers adopted GNNs to unleash themselves from extensive design space exploration, i.e., running many parallel physical design implementations to achieve the best timing closure [385]. It can be significantly fast and efficient by combining the GNN and LSTM, responsible for netlist encoding and sequential flow modeling [382].

For semiconductor manufacturing tasks, a graph could be constructed as nodes representing an operation of a job on a device and directed edges representing a relation between nodes (e.g., process flow). Graph2Vec model was adopted to learn fab states, which are the processing of lots on machines and transfer between machines and setup and maintenance activities [386].

### 4.11. Weather Forecasting

Graph-based models have shown great effectiveness in learning correlations of spatial and temporal features for weather prediction tasks. Typically, a graph is built with nodes that describe stations that collect information in different geographical locations, edges that describe the spatial neighbors of the stations, and attributes that describe meteorological variables. Meteorological variables include measurements over a specified time period, such as temperature, humidity, soil moisture, seismic source, etc. Several CGNNs have been proposed to capture spatial relations between different geographical locations [363,387,388]. The models could help to combine with an LSTM model to process temporal time series in solar radiation prediction.

Since the interactions of meteorological variables at different locations could show dynamic behaviors and mutual influence, several graph-based models could help to capture these dynamic influences. For example, Lira et al. [389] proposed spatio-temporal attention-based GNNs to predict frost by capturing the influences between round environmental sensors (nodes). GNNs could help to capture the spatial dependency patterns for predicting several weather tasks (e.g., temperature and humidity prediction) [390]. Jeon et al. [391] proposed the MST-GCN model (Multi-attributed Spatio-Temporal GCN) to predict hourly solar irradiance using GCNs to learn the spatio-temporal correlations between meteorological variables (e.g., temperature, wind speed, relative humidity, etc.). A graph could be constructed by considering each station as a node, and edges were defined in two ways: distances between stations and correlations between historical meteorological variables of stations.

For air quality prediction, several graph-based models could help to predict air quality by learning the correlations between air pollution variables (e.g., CO${}_{2}$, O${}_{3}$, etc.) and meteorological variables. Since the diffusion of air pollutants is affected by multiple factors (e.g., meteorological conditions, vehicle emissions, and industrial sources), Xiao et al. [392] used CGNNs to help predict the diffusion of PM${}_{2.5}$ concentration. A dynamic-directed graph could be constructed by considering nodes as stations, and edges denote the distance of stations that denotes the edges’ strength. Several studies [393,394] used a heterogeneous graph to represent the type of each station as a node type and the connection between them as an edge. They then adopt RGNNs to learn spatial and temporal correlations to predict air quality.

Graph-based models could also help to predict surface-related tasks, such as seismic source characterization, seismic wave analysis, and earthquakes [395,396,397]. A graph could be constructed by nodes as stations and edges are the relationships of nodes if seismic events can occur simultaneously. For example, GNNs could help to estimate earthquake location by leveraging waveform information from multiple stations [397].

Several graph-based models could help predict sea surface temperature (SST), which plays an important role in various ocean-related predictions (e.g., global warming, oceanic environmental protection, and disaster reduction) [398,399,400]. A graph could be constructed as longitude and latitude grids where nodes are coordinates and edges represent the relationship between nodes. For example, GCNs [401] could help to learn temporal shifts to predict the sea surface temperature.

A graph can be constructed as a hierarchical tree representing different variables’ influences on global-scale weather forecasting. Lam et al. [402] transformed the 3D data into a multi-resolution icosahedral network as a mesh hierarchy. GNNs could help to capture long-range spatial interactions for modeling global forecasting systems. Shi et al. [403] designed an adaptive mesh grid based on Voronoi polygons for ocean simulations and used GNNs to investigate environmental parameters for arbitrary visual mapping.

For El Niño-Southern Oscillation (ENSO) prediction and global ocean-atmosphere interaction, graph-based models could help to improve climate prediction tasks. For example, Cachay et al. [404] constructed the climate graph that defines each grid cell as a node, and the edge denotes the similarity between nodes. GNNs could help to capture correlations between spatio-temporal samples to improve the El Niño forecasting task. CGNNs is used to capture interactions of different air-sea coupling strengths in various period of time [405].

## 5. Evaluation Methods

Since we cannot evaluate the performance of learned graph embedding models, numerous benchmarks have been used to investigate the performance of various models to solve specific downstream tasks. A good graph embedding model should provide vector representations of graph entities that preserve the graph structure and entity relationship. In this section, we first discuss benchmark datasets and then examine typical downstream tasks such as classification, ranking, and regression tasks.

### 5.1. Benchmark Datasets

The goal of benchmark datasets is the standard for developing, evaluating, and comparing graph representation learning models. Table 18 presents a summary of benchmark datasets for graph embedding models. Typically, the benchmark datasets are categorized into four main groups: citation networks, social networks, webpages, and biochemical networks.

Citation networks depict a network of documents linked together in a particular manner. The citation graph could be constructed by considering each node as a document, and each edge of two nodes describes the citation. Since citations are directed from a source document to a destination document, citation graphs usually are directed graphs. Since the labels of the citation network could represent document topics, there are several downstream tasks for citation network analysis, such as link prediction and node classification.

The social networking datasets describe the connections between users on social networking sites such as Facebook [409], Twitter, or blog forums [422]. An online social network describes the links between users or groups, usually through the link of adding friends. In addition, the user properties could also be included in the graphs. Due to privacy policies, several user information could be hidden in social networks. Therefore, there are several downstream tasks for social network analysis, such as missing node classification and link prediction.

Webpage datasets are a term used to refer to a collection of webpages of information organized and linked together to represent information such as text and images. A webpage can be an article, a category, or any information page. For instance, Wikipedia dataset [406] in Table 18 is a directed network with 2405 nodes and 17,981 edges linking nodes. There are several downstream tasks for webpage analysis, such as node classification and link prediction.

Biochemical networks are data sources containing information in the field of biochemistry area. Several downstream tasks are used for the biochemical networks, such as predicting the composition of cancer classification proteins [423] or drug–drug interaction prediction. Protein dataset [411], for example, are biochemical graph sets with 1113 graphs. The protein dataset includes more than 435,000 nodes and 1,621,000 links between nodes.

### 5.2. Downstream Tasks and Evaluation Metrics

After the models learn vector embeddings, various downstream tasks can benefit from such embeddings, such as classification tasks, regression tasks, and prediction tasks. Therefore, we first discuss the downstream tasks and then examine the standard evaluation metrics for each task.

The classification problem denotes the graph entities classification tasks, including node classification, edge classification, subgraph classification, and graph classification. There are also link prediction tasks that can be considered to be classification problems where the output is discrete. The goal of classification tasks is to predict the classes of unlabeled graph entities given a set of labeled entities. For example, in the Cora citation network, the task of node classification is to classify publications grouped into seven main classes that correspond to the research area. Several evaluation metrics could be used for classification tasks, such as Accuracy (*A*), Precision (*P*), Recall (*R*), and $F_{\beta }$ score.

Consider a dataset consisting *n* multi-label examples $D=\{x_{i},Y_{i}\}$ where 1≤i≤n and $Y_{i}={\left\{0,1\right\}}^{m}$ with a labelset L: $\left|L\right|=m$. Let *C* be a multi-label classifier and ${\hat{Y}}_{i}=C\left(x_{i}\right)={\left\{0,1\right\}}^{m}$ denotes the set of the label for the classification of the sample $x_{i}$. Accuracy measures the number of correct classifications over all the number (predicted and actual) of labels for that instance. The higher the accuracy, the more accurate the models. The precision metric *P* is measured as the ratio of predicted correct labels to the total number of actual labels. The Recall metric *R* is measured as the ratio of predicted correct labels to the total number of predicted labels. In several classification tasks, where both Precision and Recall metrics are important in the model evaluation, a common metric that combines both Recall and Precision is called $F_{\beta }$-score. Mathematically, the Accuracy (*A*), Precision (*P*), Recall (*R*), and $F_{\beta }$ score for all instances could be computed as:

(176) $$\begin{array}{c} A=\frac{1}{n}\sum_{i=1}^{n}\frac{\left|Y_{i}\cap {\hat{Y}}_{i}\right|}{\left|Y_{i}\cup {\hat{Y}}_{i}\right|},P=\frac{1}{n}\sum_{i=1}^{n}\frac{\left|Y_{i}\cap {\hat{Y}}_{i}\right|}{\left|{\hat{Y}}_{i}\right|},R=\frac{1}{n}\sum_{i=1}^{n}\frac{\left|Y_{i}\cap {\hat{Y}}_{i}\right|}{\left|Y_{i}\right|},F_{\beta }^{}={\left(1+\beta \right)}^{2}\frac{PR}{\beta^{2}P+R}, \end{array}$$

where β denotes a positive factor to change the impact between *P* score and *R* score. Besides measuring based on samples, we could measure the performance based on label evaluation. This could be beneficial when the number of labels is large, and it is challenging to compute a performance snapshot. Therefore, we can compute the score in each class label first and then average over all classes (macro averaging) or across all the classes and samples (micro averaging).

Several regression metrics could be used for rating prediction in recommendation systems to evaluate the user–item interaction pairs [424,425]. Similar to graph classification, graph regression problems aim to predict the labels of entities in a graph by learning neighbor node labels. However, the difference between classification and regression problems is that the metrics for regression problems are explained in error, which measures the difference between predicted and actual labels. Another metric that is widely used for measuring the performance of regression models is the Coefficient of Discrimination ($R^{2}$). $R^{2}$ measures the ratio between the unexplained variations over total variations. The standard metrics, which are Mean Square Error (*MSE*), Root Mean Square Error (*RMSE*), and Mean Absolute Error (*MAE*), and $R^{2}$ could be computed as:

(177) $$\begin{array}{cc} \; & MSE=\frac{{∥Y_{i}-{\hat{Y}}_{i}∥}_{2}^{2}}{N},\;MAE=\sum_{i=1}^{n}\left|Y_{i}^{}-{\hat{Y}}_{i}^{}\right|,\; \end{array}$$

(178) $$\begin{array}{cc} \; & RMSE=\sqrt{\frac{{∥Y_{i}-{\hat{Y}}_{i}∥}_{2}^{2}}{N}},\;R^{2}=1-\frac{{∥Y_{i}-{\hat{Y}}_{i}∥}_{2}^{2}}{{∥Y_{i}-\bar{Y}∥}_{2}^{2}}, \end{array}$$

where $\bar{Y}$ denotes the mean of the dependent variable in the dataset.

In graph ranking tasks, the models try to predict the rank (or relevance index) of a list of items for a particular task. The models can learn the order of the predicted labels for multi-label classification problems where each sample has more than one label. For example, in the case of most recommendation systems, a user could have more than one preference. Several commonly used metrics evaluate model performance for the raking problems, including Mean Reciprocal Rank (MRR), P@k, MAP@k, and R@k.

The Mean Reciprocal Rank (*MRR*) metric is one of the simplest metrics in evaluating ranking models. The MRR metric calculates the average of the corresponding terms of the first related item for a set of queries *Q*, which can be defined as:

(179) $$MRR=\frac{1}{\left|Q\right|}\sum_{i=1}^{Q}\frac{1}{rank_{i}}\;.$$

One of the limitations of the MRR metric is that it only counts from the first item to the rank of actual labels in the query list. Precision at *k* (P@k) is a metric that could compute the proportion of the number of the first *k* predicted labels in the actual labelset over the *k*. The predicted label order is not taken into account in the P@k metric. Similar to the P@k metric, Recall@k is a metric that computes the proportion of the number of the first *k* predicted labels in the actual labelset over all relevant items.

(180) $$P@k=\frac{\left|\left\{Y_{i}\right\}\cap \left\{{\hat{Y}}_{i}\left[:k\right]\right\}\right|}{\left|\{{\hat{Y}}_{i}\left[:k\right]\}\right|}\;\;R@k=\frac{\left|\left\{Y_{i}\right\}\cap \left\{{\hat{Y}}_{i}\left[:k\right]\right\}\right|}{\left|\{{\hat{Y}}_{i}\}\right|}\;.$$

Mean Average Precision (MAP@k) can be applied to the entire dataset because of the stability in ranking the labels. Compared to P@k, MAP focuses more on how many predicted labels are in the actual labelset, where the order of predicted labels is taken into account. Mathematically, MAP@k is the average across all instances, which could be calculated as:

(181) $$MAP=\frac{1}{n}\sum_{i=1}^{n}\frac{1}{K}\sum_{k=1}^{K}P@k\times rel\left(k\right)\;$$

where rel(k) denotes the relevance at *k* for each sample.

### 5.3. Libraries for Graph Representation Learning

Several libraries provide state-of-the-art graph representation learning models which have a variety of sampling strategies and downstream tasks. To ease researchers to develop graph representation learning models, this section introduces a collection of libraries, which are summarized in Table 19.

PyTorch Geometric (PyG) [426] is a graph neural network framework based on PyTorch. PyG can handle and process large-scale graph data, multi-GPU training, multiple classic graph neural network models, and multiple commonly used graph neural network training datasets. PyG already contains numerous benchmark datasets, including Cora, Citeseer, etc. It is also effortless to initialize such a dataset, which will automatically download the corresponding dataset and process it into the required format for various GNNs. Furthermore, many real-world datasets are stored as heterogeneous graphs, which prompted the introduction of specialized functions in PyG.

Deep Graph Library (DGL) [427] is an easy-to-use, high-performance, scalable Python package for building graph representation learning models. DGL has better memory management for GNNs that can be expressed as sparse matrix multiplication. Therefore, the DGL library provides flexible, efficient strategies for building new GNN layers. Furthermore, DGL has a programming interface for flexible applications, which helps researchers understand the process of designing GNNs for large graphs.

OpenNE is a standard Network Representation Learning framework that enables graph embedding models with multi-GPU training. Most of the graph embedding models in OpenNE framework are matrix factorization-based and shallow models, including DeepWalk, LINE, Node2Vec, GraRep, TADW, GCN, HOPE, GF, and SDNE. Furthermore, the framework could also provide dimension-reduction techniques, such as t-SNE and PCA, for visualization.

Developed by Tsinghua University, CogDL [428] framework could integrate various downstream tasks and match evaluation methods. Therefore, the framework could help researchers efficiently run the results of various baseline models and develop new graph embedding models. Furthermore, the framework could integrate algorithms task-oriented and assigns each algorithm to one or more tasks. In addition, CogDL also supports researchers in customizing models and datasets and is embedded in the overall framework of CogDL to help them improve efficiency.

For complex downstream tasks, such as graph generation and graph neural network interpretability, DIG [429] provides APIs for data interfaces, commonly used algorithms and evaluation standards. DIG is designed to make it easy for researchers to develop algorithms and conduct experimental comparisons with benchmark models. The framework could help researchers solve tasks, including graph generation, graph self-supervised learning, graph neural network interpretability, and 3D graph deep-learning tasks.

GraphVite [430] is a general-purpose graph embedding framework to help researchers learn embeddings with high speed and large scale. One of the advantages of the framework is that GraphVite can support multi-GPU parallelism. Therefore, the framework could quickly handle large-scale graphs with millions of nodes and learn the node representation. GraphVite provides complete training and evaluation for various types of graphs, including homogeneous and knowledge graphs.

GraphLearn [431] is a graph learning framework designed to develop and apply large-scale GNN models in practical situations. The framework could help researchers parallel negative sampling from industrial application scenarios to speed up training. Therefore, the framework could implement sampling optimization, sparse scene model optimization, and GPU acceleration for PyTorch. As a result, GraphLearn has been successfully applied in Alibaba and several scenarios, such as recommendation systems and security risks.

Another library for graph representation learning is Connector which can help researchers develop new graph embedding models efficiently. The framework provides various widespread graph representation learning models, such as matrix factorization-based, shallow, and GNN models. Furthermore, Connector can analyze various types of graphs, ranging from homogeneous and heterogeneous graphs to knowledge graphs with different sampling processes. Therefore, Connector could help researchers efficiently construct various baseline models and design new graph embedding models.

## 6. Challenges and Future Research Directions

Graph representation learning models have gained significant results recently, showing the model’s power and practical applications in the real world. However, there are still several challenges for existing models since graph data are complicated (e.g., nodes are disordered and have a complex relationship). Therefore, this section presents challenges and promising directions for future research. The main challenges and future research directions of graph embedding models are summarized as follows:

- Graph representation in a suitable geometric space: Euclidean space may not capture the graph structure sufficiently and lead to structural information loss.
- The trade-off between the graph structure and node features: Most graph embedding models suffer from noise from non-useful neighbor node features. This could lead to a trade-off between structure preservation and node feature representation, which can be the future research direction.
- Dynamic graphs: Many real-world graphs show dynamic behaviors representing entities’ dynamic structure and properties, bringing a potential research direction.
- Over-smoothing problem: Most GNN models suffer from this problem. The graph transformer model could only handle the over-smoothing problem in several cases.
- Disassortative graphs: Most graph representation learning models suffer from this problem. Several solutions have been proposed but have yet to fully solve to the whole extent.
- Pre-trained models: Pre-trained models could be beneficial to handle the little availability of node labels. However, a few graph embedding models have been pre-trained on specific tasks and small domains.

The performance of graph embedding models is determined by how well the geometric space for graph representation matches the graph structure [292]. Therefore, choosing a suitable geometric space to represent the graph structure is a crucial step in building efficient graph representation learning models. Most existing graph embedding models represent the graph structure in Euclidean space, which defines the similarity between entities by the inner product, Euclidean distance, and so on. However, representing the graph structure in Euclidean space may not capture the graph structure sufficiently and lead to structural information loss [432]. For example, models in Euclidean space fail to represent adequate tree-like graph data where the nodes grow exponentially and follow the power law. In the case of webpage networks with millions of nodes, there are a few important websites that are hubs and dominate the network, while most other websites have few connections, which leads to most existing models in the Euclidean space failing to learn embeddings. Recently, several studies have been trying to represent graph data in the non-Euclidean space, and the results are relatively promising [69,103,432]. Nevertheless, it still needs to be resolved whether representing graph data in non-Euclidean space is more efficient and significantly improves accuracy. One major issue is the choice of suitable isometric models, and the reasons why and when to use the models are still an open question that existing models have yet to analyze to a whole extent [294]. Another problem is that developing operators and optimization in the non-Euclidean space for deep neural networks is challenging. Most existing models aim to approximate graph data in a tangent space where we are familiar with Euclidean operators. However, several studies presented that tangent space approximation could negatively influence the training phase [293,433]. Therefore, developing operators, manifold space, and optimization for various embedding models are significant problems for implementing models in non-Euclidean space.

A good graph representation learning model should preserve the graph structure and represent appropriate features for nodes in graphs. This inspires many shallow models to explore various substructures of graph data (e.g., random walk [4,14], *k*-hop distance [16], motifs [87,89,90,91], subgraphs [145], graphlets [88], and roles [21]). Several of these sampling strategies ignore the substructures surrounding target nodes [4,14,16], while others omit the node features which could also carry significant information [145]. Recently, models based on message-passing mechanisms effectively capture graph structures and represent node feature embeddings. The message-passing could suffer from noise coming from non-useful neighbor node features, which cause a barrier to the downstream tasks and eventually reduce the performance of models. There are several studies have been proposed to overcome weaknesses of message-passing, such as structural identity [60], and dropout [434,435]. However, collecting sufficient structural topology and a trade-off between structure preservation and node feature representation still needs to be explored to a full extent.

Most existing graph embedding models work with static graphs where the graph structure and entity properties do not change over time [4,14]. However, in the real world, graphs are dynamic, consisting of both graph structure and properties that evolve over time [10,82]. There are several dynamic behaviors of graph evolution, including topological evolution (the set of nodes and edges change over time), feature evolution (the node and edge feature or its label changes over time), degree distribution, and the node role changes over time. However, most existing models only aim to find out which patterns of evolution should be captured and represented that do not represent fully dynamic behaviors in general [10,112]. For example, in the case of social networks, users could change personal attributes such as hometown, occupation, and their role in a specific small group over time. This leads to how models can represent the dynamic structure and properties of entities bringing a potential research direction.

Graph neural networks have shown significant advantages in working with large-scale graphs for specific tasks. However, these existing models still have limitations regarding the over-smoothing problem when stacking more GNN layers. Recently, several works have attempted to handle the over-smoothing problem, such as adding initial residual connection [28], using dropout [436], and PageRank [437]. However, most of them need to be effectively adaptable to a wide and diverse scope of various graph structures. Several graph transformer models have been proposed in recent years to overcome the limitation of the message-passing mechanism by self-attention [63,438]. However, the self-attention mechanism considers input graphs as fully connected graphs that have yet to entirely solve the over-smoothing problems, especially in small and sparse graphs [61]. Therefore, building a deep-learning model to address the over-smoothing problem is still an open question and a promising research direction.

Another challenge for graph embedding models is the problem of working with disassortative graphs for various downstream tasks, especially classification tasks. Disassortative graphs are graphs where pairs of nodes with different labels tend to be connected. For example, in the case of amino acid networks, amino acids with different labels tend to be connected by peptide bonds [439]. Looking back at the sampling mechanism of GNNs and graph transformer models, the target nodes update the vector embeddings based on the *k*-hop neighbor features [24,310]. This is a problem for classification tasks where the aggregation mechanisms assume that interconnected nodes should have the same label, which is completely different from the disassortative graph structure. Several methods have been proposed in recent years to overcome classification problems for disassortative graphs [58,440]. However, the message-passing-based mechanisms are still a problem and challenge when working with disassortative graphs.

Another problem in challenging deep-learning models is to pre-train the graph embedding models and then fine-tune the models on various downstream tasks. Most current models are designed independently to be suitable for some specific tasks that have yet to be generalized, even with graphs in the same domain [8]. Although several graph transformer models have been pre-trained on related tasks, the transfer of the models across other tasks is still limited in a few specific graph data [30,63]. This leads to the problem that the models must train from scratch when we have new graph data and other tasks, which is time-consuming and limits practical applicability. The pre-trained models are also beneficial to handle the little availability of node labels. Therefore, if the graph embedding models are pre-trained, they could be transferred and used to handle new tasks.

## 7. Conclusions

This paper has presented a comprehensive view of graph representation learning. Specifically, most models have been discussed, ranging from traditional models, such as graph kernels and matrix factorization models, to deep-learning models with various graphs. One of the most thriving models is the GNN with the power of an aggregation mechanism in learning the local and global structures of the graph. The achievements of GNN-based models have been seen in various real-world tasks with large-scale graphs. Recently, graph transformer models have shown promising results in applying self-attention to learn embeddings. However, the self-attention mechanism need also be improved to solve the over-smoothing problem to a whole extent.

Practical applications in various fields are also presented, showing the contribution of graph representation learning to society and related areas. Our paper not only shows the applications of graph embedding models but also describes how a graph is initialized in each specific domain and the application of the graph embedding model to each application. In addition, evaluation metrics and downstream tasks were also discussed to understand more about graph embedding models. Although deep graph embedding models have shown great success in recent years, they still have several limitations. The balance between the graph structure and the node features is still challenging for deep graph embedding models in various downstream tasks. Our paper also points out the current challenges and future directions of promising research.

## Figures and Tables

**Table 1:** A summary of notations.

Notations	Descriptions
*V*	The set of nodes in the graph *G*
*E*	The set of edges in graph *G*
*N*	The number of nodes in graph *G*
$E^{t}$	The set of edges with type *t* in heterogeneous graphs
$v_{i}$	The node $v_{i}$ in the graph *G*
$e_{ij}$	The edge $\left(v_{i},v_{j}\right)$ in the graph *G*
*A*	The adjacency matrix of the graph *G*
*X*	The feature matrix of nodes in graph *G*
*D*	The degree matrix of nodes in graph *G*
ϕ	Projection function
$Z_{i}$	The embedding vector of node $v_{i}$
*M*	The transition matrix
$N(v_{i})$	The set of neighbors of node $v_{i}$
*k*	The *k*-hop distance from a target node to other nodes
*d*	The dimension of vector in latent space
$y_{i}$	The label of node $v_{i}$

**Table 2:** A summary of graph kernel models.

Models	Graph Types	Tasks	Loss Function
[83]	Static graphs	Graph comparison	$\sum_{v_{i}\in V}\max\left(0,1-y_{i}^{⊺}{\hat{y}}_{i}\right)+L_{2}$
[106]	Static graphs	Graph comparison	$\sum_{v_{i}\in V}\max\left(0,1-y_{i}^{⊺}{\hat{y}}_{i}\right)+L_{2}$
[33]	Static graphs	Graph classification	$\sum_{v_{i}\in V}\max\left(0,1-y_{i}^{⊺}{\hat{y}}_{i}\right)+L_{2}$
[34]	Static graphs	Graph classification	$\sum_{v_{i}\in V}\max\left(0,1-y_{i}^{⊺}{\hat{y}}_{i}\right)+L_{2}$
[84]	Static graphs	Graph classification	$\sum_{v_{i}\in V}\max\left(0,1-y_{i}^{⊺}{\hat{y}}_{i}\right)+L_{2}$
[35]	Static graphs	Graph classification	$\sum_{v_{i}\in V}\max\left(0,1-y_{i}^{⊺}{\hat{y}}_{i}\right)+L_{2}$
[85]	Static graphs	Graph comparison	$\sum_{v_{i}\in V}\max\left(0,1-y_{i}^{⊺}{\hat{y}}_{i}\right)+L_{2}$
[107]	Attributed graphs	Graph classification	$\sum_{v_{i}\in V}\max\left(0,1-y_{i}^{⊺}{\hat{y}}_{i}\right)+L_{2}$
[37]	Attributed graphs	Graph classification	$\sum_{v_{i}\in V}\max\left(0,1-y_{i}^{⊺}{\hat{y}}_{i}\right)+L_{2}$
[108]	Attributed graphs	Graph classification	$\sum_{v_{i}\in V}\max\left(0,1-y_{i}^{⊺}{\hat{y}}_{i}\right)+L_{2}$
[36]	Attributed graphs	Graph classification	$\sum_{v_{i}\in V}\max\left(0,1-y_{i}^{⊺}{\hat{y}}_{i}\right)+L_{2}$
[109]	Attributed graphs	Graph classification	$\sum_{v_{i}\in V}\max\left(0,1-y_{i}^{⊺}{\hat{y}}_{i}\right)+L_{2}$
[110]	Attributed graphs	Graph classification	$\sum_{v_{i}\in V}\max\left(0,1-y_{i}^{⊺}{\hat{y}}_{i}\right)+L_{2}$
GraTFEL [111]	Dynamic graphs	Graph reconstruction Link prediction	$\frac{1}{N}\sum_{v_{i}\in V}{∥Z_{i}-{\hat{Z}}_{i}∥}_{2}^{2}+L_{1}+L_{2}$
[112]	Dynamic graphs	Link prediction	$\frac{1}{N}\sum_{v_{i}\in V}{∥Z_{i}-{\hat{Z}}_{i}∥}_{2}^{2}+L_{1}+L_{2}$
[113]	Dynamic graphs	Link prediction	$\frac{1}{N}\sum_{v_{i}\in V}{∥Z_{i}-{\hat{Z}}_{i}∥}_{2}^{2}$

**Table 6:** A summary of structure-preservation models for heterogeneous graphs and dynamic graphs. *K* is the number of clusters in graphs, $N_{neg}$ refers to the number of negative samples, and $P_{n}$ means the noise distribution.

Models	Graph Types	Tasks	Loss Function
MBRep [90]	Hypergraphs	Link prediction	$-\sum_{v_{i}\in V,\langle v_{i},v_{j}\rangle \in E}\log\left(\sigma (Z_{i}^{⊺}Z_{j})\right)-\left|N_{neg}\right|\sum_{v_{k}∼P_{n}\left(v\right)}\log\left(\sigma (-Z_{i}^{⊺}Z_{k})\right)$
Motif2Vec [87]	Heterogeneous graphs	Node classification link prediction	$\sum_{v_{i}\in V}\sum_{v_{j}\in N\left(v_{i}\right)}-\log\left(p(v_{j}|Z_{i})\right)$
JUST [159]	Heterogeneous graphs	Node classification Node clustering	$\sum_{(v_{i},v_{j})\in E}\log\sigma \left(Z_{i}Z_{j}\right)+\left|N_{neg}\right|E_{v_{k}∼P_{n}\left(v_{k}\right)}\left(\log\sigma (-Z_{i}Z_{k})\right)$
[160]	Multiplex graphs	Link prediction	$\sum_{(v_{i},v_{j})\in E}\log\sigma \left(Z_{i}Z_{j}\right)+\left|N_{neg}\right|E_{v_{k}∼P_{n}\left(v_{k}\right)}\left(\log\sigma (-Z_{i}Z_{k})\right)$
BHIN2Vec [161]	Heterogeneous graph	Node classification	$-\sum_{v_{i}\in V}\left(y_{i}^{⊺}\log\left({\hat{y}}_{i}\right)+\left(1-y_{i}\right)\log\left(1-{\hat{y}}_{i}\right)\right)$
[162]	Heterogeneous graphs	Link prediction	$\frac{1}{\left|V\right|}\sum_{v_{i}\in V}{∥y_{i}-{\hat{y}}_{i}∥}_{2}^{2}$
[163]	Heterogeneous graphs	Link prediction	$\frac{1}{\left|V\right|}\sum_{v_{i}\in V}{∥y_{i}-{\hat{y}}_{i}∥}_{2}^{2}$
[164]	Heterogeneous graphs	Link prediction	$\frac{1}{\left|V\right|}\sum_{v_{i}\in V}{∥y_{i}-{\hat{y}}_{i}∥}_{2}^{2}$
[165]	Heterogeneous graphs	Link prediction	$\frac{1}{\left|V\right|}\sum_{v_{i}\in V}{∥y_{i}-{\hat{y}}_{i}∥}_{2}^{2}$
[166]	Heterogeneous graphs	Entities prediction	$\frac{1}{\left|V\right|}\sum_{v_{i}\in V}{∥y_{i}-{\hat{y}}_{i}∥}_{2}^{2}$
MrMine [167]	Multiplex graphs	Graph classification	$-\sum_{v_{i}\in V,\langle v_{i},v_{j}\rangle \in E}\log\left(\sigma (Z_{i}^{⊺}Z_{j})\right)-\left|N_{neg}\right|\sum_{v_{k}∼P_{n}\left(v\right)}\log\left(\sigma (-Z_{i}^{⊺}Z_{k})\right)$
[168]	Heterogeneous graph	Link prediction	$\sum_{(v_{i},v_{j})\in E}\left[\log\sigma \left(Z_{i}^{⊺}Z_{j}\right)-\sum_{k=1}^{n}\left(E_{v_{k}∼P\left(v_{i}\right)}\log\sigma \left(Z_{i}^{⊺}Z_{k}\right)\right)\right]$
[169]	Dynamic graphs	Node classification	$-\sum_{v_{i}\in V}y_{i}^{⊺}\log\left({\hat{y}}_{i}\right)$
[170]	Dynamic graphs	Node classification	$-\sum_{v_{i}\in V,\langle v_{i},v_{j}\rangle \in E}\log\left(\sigma (Z_{i}^{⊺}Z_{j})\right)-\left|N_{neg}\right|\sum_{v_{k}∼P_{n}\left(v\right)}\log\left(\sigma (-Z_{i}^{⊺}Z_{k})\right)$
[171]	Dynamic graphs	Link prediction	$-\sum_{i=1}^{N}\left[y_{i}\log{\hat{y}}_{i}+\alpha \left(1-y_{i}\right)\log\left(1-{\hat{y}}_{i}\right)\right]$
STWalk [172]	Dynamic graphs	Node classification	
[173]	Dynamic graphs	Node classification, Link prediction	$-\sum_{v_{i}\in V}y_{i}^{⊺}\log\left({\hat{y}}_{i}\right)$
[174]	Dynamic graphs	Link prediction, Node classification	$-\sum_{v_{i}\in V}\sum_{v_{j}\in N\left(v_{i}\right)}\log({\hat{Z}}_{j})$
Dyn2Vec [10]	Dynamic graphs	Node classification	$-\sum_{v_{i}\in V,\langle v_{i},v_{j}\rangle \in E}\log\left(\sigma (Z_{i}^{⊺}Z_{j})\right)-\left|N_{neg}\right|\sum_{v_{k}∼P_{n}\left(v\right)}\log\left(\sigma (-Z_{i}^{⊺}Z_{k})\right)$
[92]	Dynamic graphs	Link prediction	$\frac{1}{\left|V\right|}\sum_{v_{i}\in V}\left[y_{i}\log{\hat{y}}_{i}+\alpha \left(1-y_{i}\right)\log(1-{\hat{y}}_{i}\right]$
T-EDGE [175]	Dynamic graphs	Node classification	$-\sum_{v_{i}\in V,\langle v_{i},v_{j}\rangle \in E}\log\left(\sigma (Z_{i}^{⊺}Z_{j})\right)-\left|N_{neg}\right|\sum_{v_{k}∼P_{n}\left(v\right)}\log\left(\sigma (-Z_{i}^{⊺}Z_{k})\right)$
LBSN2Vec [157]	Hyper graphs	Link prediction	$\sum_{i=1}^{n}\left(1-\cos\left(Z_{i},Z_{j}\right)\right)$
[158]	Hyper graphs	Link prediction	$\underset{Z}{\arg\min}\;Z^{⊺}LZ$

**Table 8:** A summary of fully connected graph autoencoder models. *A* and $\hat{A}$ are the input adjacency matrix and reconstructed adjacency matrix, respectively, *B* is the penalty matrix, $A^{t}$ is the adjacency matrix of node type *t*, *L* denotes the number of layers, *k* is the length of random-walk steps, $s_{ij}$ denotes the proximity between $v_{i}$ and $v_{j}$, and $Z_{i}^{\left(l\right)}$ is the hidden vector of node $v_{i}$ at layer *l*.

Models	Graph Types	Objective	Loss Function
SDNE [50]	Static graphs	1st-order proximity, 2nd-order proximity	${∥(\hat{A}-A)⊙B∥}_{F}^{2}+\lambda \sum_{i,j=1}^{n}s_{ij}{∥Z_{i}-Z_{j}∥}_{2}^{2}+L_{2}$.
DHNE [51]	Hyper graphs	1st-order proximity, 2nd-order proximity	${∥\hat{A}-A∥}_{F}^{2}+\lambda \sum_{t}^{n}{∥A^{t}-{\hat{A}}^{t}∥}_{F}^{2}$.
DNE-SBP [197]	Signed graphs	1st-order proximity	$\sum_{l=1}^{L}\left({∥({\hat{A}}^{\left(l\right)}-A^{\left(l\right)})⊙B∥}_{F}^{2}+\alpha_{l}A{∥Z_{i}^{\left(l\right)}-Z_{j}^{\left(l\right)}∥}_{F}^{2}+\beta_{l}L_{1}+\gamma_{l}L_{2}\right)$.
DynGEM [198]	Dynamic graphs	1st-order proximity, 2nd-order proximity	${∥(\hat{A}-A)⊙B∥}_{F}^{2}+\lambda \sum_{i,j=1}^{n}s_{ij}{∥Z_{i}-Z_{j}∥}_{2}^{2}+L_{1}+L_{2}$.
NetWalk [199]	Dynamic graphs	Random walk	$\sum_{l=1}^{L}{∥Z_{i}^{\left(l\right)}-Z_{j}^{\left(l\right)}∥}_{2}^{2}+\sum_{l=1}^{L}{∥|A_{i}^{\left(l\right)}-A_{j}^{\left(l\right)}∥}_{2}^{2}+L_{2}$.
DNGR [196]	Static graphs	PPMI matrix	${∥\hat{A}-A∥}_{F}^{2}$.

**Table 9:** A summary of graph recurrent autoencoder models. $G_{i,t}$ is the diffusion graph of a cascade at time *t*, $y_{i}$ is the label of node $v_{i}$, *T* is the timestamp window, $A_{ij}^{t}$ is the adjacency matrix at time *t*, σ(·) is the sigmoid function. $w_{i,j}$ is the weight between two nodes $v_{i}$ and $v_{j}$, $N_{s}\left(v_{i}\right)$ is the set of neighbors of node $v_{i}$, and triple $\left(v_{i},v_{j},v_{k}\right)$ denotes $(v_{i},v_{j})\in P$, and $v_{k}$ is the negative sample.

Model	Graph Type	Sampling Strategy	Loss Function
[44]	Hypergraphs	Local transition function	$\sum_{v_{i}\in V}{∥Z_{i}-{\hat{Z}}_{i}∥}_{2}^{2}$
[45]	Homogeneous graphs	Local transition function	$\sum_{v_{i}\in V}{∥Z_{i}-{\hat{Z}}_{i}∥}_{2}^{2}$
[57]	Weighted graphs	Node-weight sequences	$\frac{1}{N}\sum_{v_{i}\in V}{∥Z_{i}-{\hat{Z}}_{i}∥}_{2}^{2}$, $\sum_{<v_{i},v_{j}>\in E}^{}w_{ij}\log\left(p(v_{i},v_{j})\right)$ *p*(*v*_*i*_, *v_j_*) = SoftMax ($Z_{i}^{⊺}$*Z*_*j*_)
[202]	Dynamic graphs	Random walk, Shortest paths, BFS	$\frac{1}{N}\sum_{v_{i}\in V}{∥Z_{i}-{\hat{Z}}_{i}∥}_{2}^{2}$
LSTM-Node2Vec [203]	Dynamic graphs	Temporal random walk	$-\sum_{v_{i}\in V}\log p\left(N_{s}\left(v_{i}\right)|Z_{i}\right)$
E-LSTM-D [204]	Dynamic graphs	1st-order proximity	${∥(A_{t}-{\hat{A}}_{t})⊙B∥}_{F}^{2}+\lambda L_{2}$
Dyngraph2Vec-AERNN [201]	Dynamic graphs	Adjacency matrix	${∥(A_{t}-{\hat{A}}_{t})⊙B∥}_{F}^{2}$
Topo-LSTM [49]	Directed graphs	Diffusion structure	$-\sum_{v_{i}\in V}\sum_{t=1}^{T}\log p\left(v_{i,t}|G_{t}\right)+\alpha L_{2}$
SHNE [48]	Heterogeneous graphs	Random walk Meta-path	$\sum_{\langle v_{i},v_{j},v_{k}\rangle }\log\sigma \left(Z_{j}\cdot Z_{i}\right)+\log\sigma \left(-Z_{k}\cdot Z_{i}\right)$
[17]	Directed graphs	Transition matrix	$-\sum_{v_{i}\in V}y_{i}^{⊺}\log\left({\hat{y}}_{i}\right)$
GraphRNA [93]	Attributed graph	Random walk	$-\sum_{v_{i}\in V}y_{i}^{⊺}\log\left({\hat{y}}_{i}\right)$
[205]	Labeled graphs	Random walks, shortest paths, and breadth-first search.	$\sum_{v_{i}\in V}{∥Z_{i}-{\hat{Z}}_{i}∥}_{2}^{2}$ $-\sum_{v_{i}\in V}y_{i}^{⊺}\log\left({\hat{y}}_{i}\right)$
[206]	Dynamic graphs	Graph reconstruction	$\sum_{t=1}^{T}\sum_{<v_{i},v_{j}>\in E}^{}A_{i,j}^{t}\log\left({\hat{A}}_{i,j}^{t}\right)$
Camel [207]	Heterogeneous graphs	Link prediction	$\sum_{\begin{array}{c} (v_{i},v_{j})\in E\\ (v_{i},v_{k})\notin E \end{array}}\left[{∥Z_{i}-Z_{j}∥}_{2}^{2}-{∥Z_{i}-Z_{k}∥}_{2}^{2}\right]$ $+\alpha_{1}\sum_{\left(v_{1},v_{k}\notin P\right)}\log\sigma (-Z_{i}^{T}Z_{j})+\alpha_{2}L_{2}$
TaPEm [208]	Heterogeneous graphs	Link prediction	$-\sum_{v_{i}\in V}\left(y_{i}^{⊺}\log\left({\hat{y}}_{i}\right)+\left(1-y_{i}\right)log\left(1-{\hat{y}}_{i}\right)\right)$
[209]	Heterogeneous graphs	Link prediction	$-\sum_{v_{i}\in V}\left(y_{i}^{⊺}\log\left({\hat{y}}_{i}\right)+\left(1-y_{i}\right)log\left(1-{\hat{y}}_{i}\right)\right)$

**Table 10:** A summary of spectral CGNN models.

Model	Graph Type	Tasks	Loss Function
[56]	Static graphs	Node classification	$-\sum_{v_{i}\in V}y_{i}^{⊺}\log\left({\hat{y}}_{i}\right)$
[96]	Static graphs	Node classification	$-\sum_{v_{i}\in V}y_{i}^{⊺}\log\left({\hat{y}}_{i}\right)+L_{2}$
[210]	Static graphs	Multi-task prediction Node classification	${\left[\frac{1}{\left|V\right|}\sum_{v_{i}\in V}{\left({\hat{y}}_{i}-y_{i}^{}\right)}^{2}\right]}^{\frac{1}{2}}$
[211]	Static graphs	Label classification	$-\frac{1}{\left|V\right|}\sum_{v_{i}\in V}y_{i}^{⊺}\log\left({\hat{y}}_{i}\right)+L_{2}$
GCN [18]	Knowledge graphs	Node classification	$-\sum_{v_{i}\in V}y_{i}^{⊺}\log\left({\hat{y}}_{i}\right)$
EGCN [55]	Static graphs	Multi-task classification, Link prediction	${\left[\frac{1}{\left|V\right|}\sum_{v_{i}\in V}{\left({\hat{y}}_{i}-y_{i}^{}\right)}^{2}\right]}^{\frac{1}{2}}$
LNPP [212]	Static graphs	Graph Reconstruction	${∥A-\hat{A}∥}_{F}^{2}$
[213]	Static graphs	Node classification	$\sum_{v_{i}\in V}{∥y_{i}-{\hat{y}}_{i}∥}_{D}^{2})$
[214]	Static graphs	Node classification	$-\sum_{v_{i}\in V}y_{i}^{⊺}\log\left({\hat{y}}_{i}\right)$
[215]	Heterogeneous graphs	Node classification	$\sum_{(v_{i},v_{j})\in E}\log\sigma \left(Z_{i}^{⊺}Z_{j}\right)-\sum_{(v_{i},v_{k})\notin E}\log\sigma \left(-Z_{i}^{⊺}Z_{j}\right)$

**Table 11:** A summary of spatial CGNN models for static and homogeneous graphs. *m* is the total weight of the degrees of the Graph, $V_{t}$ is the number of clusters in the graph. $P_{n}\left(v\right)$ is a negative sampling distribution, $A^{\left(k\right)}$ is the transition matrix at time *k*, and *B* is the batch of nodes used to calculate the gradient estimation.

Model	Graph Type	Tasks	Loss Function
HCNP [217]	Static graphs	Node classification	$-\sum_{v_{i}\in V}y_{i}^{⊺}\log\left({\hat{y}}_{i}\right)$
CDMG [218]	Static graphs	Community detection	$-trace\left(H^{⊺}A^{\left(k\right)}H\right)$
[219]	Static graphs	Passenger Prediction	${\left[\frac{1}{\left|V\right|}\sum_{v_{i}\in V}{\left({\hat{y}}_{i}-y_{i}^{}\right)}^{2}\right]}^{\frac{1}{2}}$
ST-GDN [220]	Static graphs	Link prediction	$\sum_{v_{i}\in V}{∥y_{i}-{\hat{y}}_{i}∥}_{2}^{2}$
[221]	Static graphs	Node classification	$-\sum_{v_{i}\in V}y_{i}^{⊺}\log\left({\hat{y}}_{i}\right)$
MPNNs [222]	Static graphs	Node prediction	$\sum_{v_{i}\in V}{∥y_{i}-{\hat{y}}_{i}∥}_{2}^{2}$
GraphSAGE [22]	Static graphs	Node classification	$\sum_{v_{i}\in V}-\log\left(\sigma (y_{i}^{⊺}y_{j})\right)-\left|N_{neg}\right|E_{v_{k}∼P_{n}\left(v\right)}\log\left(\sigma (-y_{i}^{⊺}y_{k})\right)$
FastGCN [52]	Static graphs	Node classification, link prediction	$\sum_{v_{i}\in V}{∥y_{i}-{\hat{y}}_{i}∥}_{2}^{2}$
SACNNs [223]	Static graphs	Node classification Regression tasks	$-\sum_{v_{i}\in V}y_{i}^{⊺}\log\left({\hat{y}}_{i}\right)$ $\frac{1}{\left|V\right|}\sum_{v_{i}\in V}{∥y_{i}-{\hat{y}}_{i}∥}_{2}^{2}$
Cluster-GCN [95]	Static graphs	Node classification	$-\frac{1}{\left|B\right|}\sum_{v_{i}\in B}y_{i}^{⊺}\log\left({\hat{y}}_{i}\right)$
[18]	Static graphs	Node classification	$\sum_{v_{i}\in V}{∥y_{i}-{\hat{y}}_{i}∥}_{2}^{2}$
[224]	Static graphs	Node classification	$-\frac{1}{\left|B\right|}\sum_{v_{i}\in B}y_{i}^{⊺}\log\left({\hat{y}}_{i}\right)$
GraphSAINT [53]	Static graphs	Node classification Community prediction	$\sum_{v_{i}\in V}{∥y_{i}-{\hat{y}}_{i}∥}_{2}^{2}$
VGAE [72]	Static graphs	Link prediction	$E_{q(Z|X,A)}\left[\log p(A|Z)\right]-KL\left[q(Z|X,A)||p(Z)\right]$
PinSAGE [225]	Static graphs	Link prediction	$E_{n_{k}∼P_{n}\left(i\right)}\max\left(0,Z_{i}\cdot Z_{n_{k}}-Z_{i}\cdot Z_{j}+m\right)$
Hi-GCN [54]	Static graphs	Classification tasks	$-\sum_{v_{i}\in V}y_{i}^{⊺}\log\left({\hat{y}}_{i}\right)$
[226]	Static graphs	Link prediction	$\sum_{\langle v_{i},v_{j},v_{k}\rangle }\log\sigma \left(Z_{i}Z_{j}-Z_{i}Z_{k}\right)+\alpha L_{2}$
[28]	Static graph	Node classification	$\frac{1}{2}\sum_{v_{i}\in V}{∥y_{i}-{\hat{y}}_{i}∥}_{2}^{2}+\alpha L_{1}$
[26]	Static graph	Node classification	$-\frac{1}{\left|V\right|}\sum_{v_{i}\in V}\left(y_{i}^{⊺}\log\left({\hat{y}}_{i}\right)+\left(1-y_{i}\right)log\left(1-{\hat{y}}_{i}\right)\right)$
[227]	Static graphs	Classification tasks	$-\frac{1}{\left|V\right|}\sum_{v_{i}\in V}\left(y_{i}^{⊺}\log\left({\hat{y}}_{i}\right)+\left(1-y_{i}\right)log\left(1-{\hat{y}}_{i}\right)\right)$
[228]	Static graph	Node Classification Link prediction	$\sum_{v_{i}\in V}{∥y_{i}-{\hat{y}}_{i}∥}_{2}^{2}$
[229]	Static graphs	Node classification Link prediction	$-\sum_{v_{i}\in V}\left(y_{i}^{⊺}\log\left({\hat{y}}_{i}\right)+\left(1-y_{i}\right)log\left(1-{\hat{y}}_{i}\right)\right)$
DCRNN [230]	Static graphs	Node classification	$\frac{1}{\left|V\right|}\sum_{v_{i}\in V}\left|y_{i}-{\hat{y}}_{i}\right|$
PinSAGE [225]	Static graphs	Link prediction	$E_{n_{k}∼P_{n}\left(i\right)}\max\left(0,Z_{i}\cdot Z_{n_{k}}-Z_{i}\cdot Z_{j}+m\right)$
E-GraphSAGE [231]	Static graph	Edge classification	$-\sum_{v_{i}\in V}y_{i}^{⊺}\log\left({\hat{y}}_{i}\right)$
GraphNorm [232]	Static graphs	Graph classification	$\sum_{v_{i}\in V}\frac{1}{2}{∥y_{i}-{\hat{y}}_{i}∥}_{2}^{2}$
GIN [24]	Heterogeneous graphs	Node classification, Graph classification	$\sum_{v_{i}\in V}\frac{1}{2}{∥y_{i}-{\hat{y}}_{i}∥}_{2}^{2}$
DeeperGCN [98]	Static graphs	Node property prediction, Graph property prediction	$\sum_{v_{i}\in V}\frac{1}{2}{∥y_{i}-{\hat{y}}_{i}∥}_{2}^{2}$
PHC-GNNs [233]	Static graphs	Graph classification	$-\sum_{v_{i}\in V}\left(y_{i}^{⊺}\log\left({\hat{y}}_{i}\right)+\left(1-y_{i}\right)log\left(1-{\hat{y}}_{i}\right)\right)$
HGNN [27]	Hypergraphs	Node classification, Recognition tasks.	$-\sum_{v_{i}\in V}y_{i}^{⊺}\log\left({\hat{y}}_{i}\right)$
HyperGCN [234]	Hypergraphs	Node classification	$-\sum_{v_{i}\in V}y_{i}^{⊺}\log\left({\hat{y}}_{i}\right)$

**Table 12:** A summary of spatial CGNN models for dynamic and heterogeneous graphs, *m* is the margin.

Model	Graph Type	Tasks	Loss Function
SHARE [235]	Dynamic graphs	Availability prediction	$\frac{1}{N}\left(\sum_{v_{i}\in V}{∥y_{i}-{\hat{y}}_{i}∥}_{2}^{2}+y_{i}^{⊺}\log\left({\hat{y}}_{i}\right)\right)$
Dyn-GRCNN [236]	Dynamic graphs	Traffic flow forecasting	$\sum_{v_{i}\in V}{∥y_{i}-{\hat{y}}_{i}∥}_{2}^{2}$ ${\left[\frac{1}{\left|V\right|}\sum_{v_{i}\in V}{\left({\hat{y}}_{i}-y_{i}^{}\right)}^{2}\right]}^{\frac{1}{2}}$
STAN [237]	Dynamic graphs	Fraud detection	$\sum_{v_{i}\in V}\left[y_{i}^{⊺}log\left({\hat{y}}_{i}\right)+\alpha \left(1-y_{i}^{⊺}\right)log\left(1-{\hat{y}}_{i}\right)\right]$
SeqGNN [238]	Dynamic graphs	Traffic speed prediction	$\sum_{v_{i}\in V}{∥y_{i}-{\hat{y}}_{i}∥}_{2}^{2}$
DMVST-Net [239]	Dynamic graphs	Taxi demand prediction	$\sum_{v_{i}\in V}\left({∥y_{i}-{\hat{y}}_{i}∥}_{2}^{2}+{∥\frac{y_{i}-{\hat{y}}_{i}}{y_{i}}∥}_{2}^{2}\right)$
ST-ResNet [240]	Dynamic graphs	Flow prediction	$\sum_{v_{i}\in V}{∥y_{i}-{\hat{y}}_{i}∥}_{2}^{2}$
R-GCNs [241]	Knowledge graphs	Entity classification	$-\sum_{v_{i}\in V}y_{i}^{⊺}\log\left({\hat{y}}_{i}\right)$
HDMI [242]	Multiplex graphs	Node clustering, Node classification	$-\frac{1}{\left|V\right|}\sum_{v_{i}\in V}\left(y_{i}^{⊺}\log\left({\hat{y}}_{i}\right)+\left(1-y_{i}\right)log\left(1-{\hat{y}}_{i}\right)\right)$
DMGI [243]	Multiplex graphs	Link Prediction, Clustering, Node classification	$-\frac{1}{\left|V\right|}\sum_{v_{i}\in V}\left(y_{i}^{⊺}\log\left({\hat{y}}_{i}\right)+\left(1-y_{i}\right)log\left(1-{\hat{y}}_{i}\right)\right)$
LDANE [244]	Dynamic graphs	Graph reconstruction, Link prediction, Node classification	$\sum_{v_{i}\in V}{∥{\hat{A}}_{i}-A_{i}∥}_{2}^{2}+\alpha \sum_{\langle v_{i},v_{j}\rangle \in E}{∥Z_{i}-Z_{j}∥}_{2}^{2}+L_{1}+L_{2}$
EvolveGCN [245]	Dynamic graphs	Link prediction, Node, edge classification	$-\sum_{v_{i}\in V}y_{i}^{⊺}\log\left({\hat{y}}_{i}\right)$

**Table 13:** A summary of attentive convolutional GNN models. $p_{ij}^{l}$ denotes the probability of an edge between two node $v_{i}$ and $v_{j}$ at layer *l*, $p_{ij}^{}=\sigma \left(W(h_{i}||h_{j})\right)$.

Model	Graph Type	Tasks	Loss Function
GAT [19]	Static graphs	Node classification	$-\sum_{v_{i}\in V}y_{i}^{⊺}\log\left({\hat{y}}_{i}\right)$
GATv2 [58]	Static graphs	Link prediction, Graph prediction, Node classification	$-\sum_{v_{i}\in V}y_{i}^{⊺}\log\left({\hat{y}}_{i}\right)$
Gaan [255]	Static graphs	Node classification	$-\sum_{v_{i}\in V}y_{i}^{⊺}\log\left({\hat{y}}_{i}\right)$
GraphStar [256]	Static graphs	Node classification, Graph classification, Link prediction	$-\sum_{v_{i}\in V}y_{i}^{⊺}\log\left({\hat{y}}_{i}\right)$
HAN [25]	Heterogeneous graphs	Node classification, Node clustering	$-\sum_{v_{i}\in V}y_{i}^{⊺}\log\left({\hat{y}}_{i}\right)$
[257]	Static graphs	Label-agreement prediction, Link prediction	$-\sum_{v_{i}\in V}\left(y_{i}^{⊺}\log\left({\hat{y}}_{i}\right)+\left(1-y_{i}\right)log\left(1-{\hat{y}}_{i}\right)\right)$
SuperGAT [258]	Static graphs	Label-agreement Link prediction	$-\frac{1}{\left|V\right|}\sum_{v_{i}\in V}\left(y_{i}^{⊺}\log\left({\hat{y}}_{i}\right)+\left(1-y_{i}\right)log\left(1-{\hat{y}}_{i}\right)\right)+\alpha \sum_{l=1}^{L}L_{E}^{l}+\beta L_{2}$ where $L_{E}^{l}=\sum_{\left(v_{i},v_{j}\right)}1_{\left(\left(v_{i},v_{j}\right)\in E\right)}\log p_{ij}^{l}+1_{\left(\left(v_{i},v_{j}\right)\notin E\right)}\log\left(1-p_{ij}^{l}\right)$
CGAT [259]	Static graphs	Node classification	$\sum_{v_{i}\in V}\sum_{\begin{array}{c} v_{j}\in N\left(v_{i}\right),\\ v_{k}\notin N\left(v_{i}\right) \end{array}}\max\left(0,\phi_{ik}^{}+m-\phi_{ij}^{}\right)-\sum_{v_{i}\in V}y_{i}^{⊺}\log\left({\hat{y}}_{i}\right)$
[260]	Static graphs	Node classification	$-\sum_{v_{i}\in V}y_{i}^{⊺}\log\left({\hat{y}}_{i}\right)$
[261]	Static graphs	Node classification, Object recognition	$-\sum_{v_{i}\in V}y_{i}^{⊺}\log\left({\hat{y}}_{i}\right)+\alpha \sum_{(v_{i},v_{j})\in E}{∥Z_{i}-Z_{j}∥}_{2}^{2}$
[25]	Heterogeneous graphs	Node classification, Node clustering	$-\sum_{v_{i}\in V}y_{i}^{⊺}\log\left({\hat{y}}_{i}\right)$
[262]	Knowledge graphs	Relation prediction	$\sum_{\langle h,t,r\rangle }\sum_{\langle h^{\prime },t^{\prime },r\rangle }\max\left(0,{∥h+r-t∥}_{1}-{∥h^{\prime }+r-t^{\prime }∥}_{1}+m\right)$
[224]	Static graphs	Node classification	$-\frac{1}{\left|V\right|}\sum_{v_{i}\in V}y_{i}^{⊺}\log\left({\hat{y}}_{i}\right)$
R-GCN [241]	Knowledge graphs	Entity classification, Link prediction	$-\frac{1}{\left|V\right|}\sum_{v_{i}\in V}y_{i}^{⊺}\log\left({\hat{y}}_{i}\right)$
DMGI [243]	Attributed multiplex graphs	Node clustering, Node classification	$-\frac{1}{\left|V\right|}\sum_{v_{i}\in V}\left(y_{i}^{⊺}\log\left({\hat{y}}_{i}\right)+\left(1-y_{i}\right)log\left(1-{\hat{y}}_{i}\right)\right)+\alpha L_{E}+\beta L_{2}$ where $L_{E}={\left[Z-\sigma \left(H^{\left(r\right)}|r\in R\right)\right]}^{2}-{\left[Z-\sigma \left({\tilde{H}}^{\left(r\right)}|r\in R\right)\right]}^{2}$
SHetGCN [263]	Heterogeneous graphs	Node classification	$-\frac{1}{\left|V\right|}\sum_{v_{i}\in V}y_{i}^{⊺}\log\left({\hat{y}}_{i}\right)$
DualHGCN [264]	Multiplex bipartite graphs	Node classification Link prediction	$\sum_{(v_{i},v_{j})\in E}\left[\alpha \log\sigma \left(Z_{i}^{⊺}Z_{j}\right)+\left(1-\alpha \right)\sum_{k=1}^{n}\left(E_{v_{k}∼P\left(v_{i}\right)}\log\sigma \left(Z_{i}^{⊺}Z_{k}\right)\right)\right]$
HANE [265]	Heterogeneous graphs	Node classification	$-\frac{1}{\left|V\right|}\sum_{v_{i}\in V}y_{i}^{⊺}\log\left({\hat{y}}_{i}\right)$
MHGCN [266]	Multiplex heterogeneous Graph	Link prediction Node classification	$-\frac{1}{\left|V\right|}\sum_{v_{i}\in V}y_{i}^{⊺}\log\left({\hat{y}}_{i}\right)$ $\sum_{(v_{i},v_{j})\in E}\log\sigma \left(Z_{i}^{⊺}Z_{j}\right)-\sum_{(v_{i},v_{k})\notin E}\log\sigma \left(-Z_{i}^{⊺}Z_{j}\right)$

**Table 14:** A summary of graph convolutional autoencoder models. *E* is the edge attribute tensor, *X* is a node attribute matrix.

Algorithms	Graph Types	Tasks	Loss Function
GAE [72]	Static graphs	Link prediction	$E_{q(Z|X,A)}\left[\log p(A|Z)\right]-KL\left[q(Z|X,A)||p(Z)\right]$
VGAE [72]	Static graphs	Link prediction	$E_{q(Z|X,A)}\left[\log p(A|Z)\right]-KL\left[q(Z|X,A)||p(Z)\right]$
[271]	Static graphs	Graph generation	$-\alpha_{1}\log p\left(A|Z\right)-\alpha_{2}\log p\left(X|Z\right)-\alpha_{3}\log p\left(E|Z\right)$
[272]	Static graphs	Graph generation	$-\sum_{v_{i}\in V}\left(y_{i}^{⊺}\log\left({\hat{y}}_{i}\right)+\left(1-y_{i}\right)log\left(1-{\hat{y}}_{i}\right)\right)$
MGAE [270]	Static graphs	Graph clustering	${∥A-\hat{A}∥}^{2}$
[273]	Static graphs	Graph reconstruction	$\frac{1}{\left|V\right|}\sum_{v_{i}\in V}{∥y_{i}-{\hat{y}}_{i}∥}_{2}^{2}$
LDANE [244]	Dynamic graphs	Graph reconstruction, Link prediction, Node classification	$\sum_{v_{i}\in V}{∥{\hat{A}}_{i}-A_{i}∥}_{2}^{2}+\alpha \sum_{\langle i,j\rangle \in E}{∥Z_{i}-Z_{j}∥}_{2}^{2}+L_{1}+L_{2}$

**Table 15:** A summary of graph transformer models. MP is the message-passing, SPD is the shortest-path distance.

Model	Graph Type	Transformer Type	Sampling Strategy	Self-Supervised Learning
[64]	Tree-like graphs	Structural encoding	Dependency path BFS, DFS	Structure reconstruction
[65]	Tree-like graphs	Structural encoding	Dependency path	Structure reconstruction
[282]	Tree-like graphs	Structural encoding	SPD	Structure reconstruction
Graph-Bert [63]	Static graphs	Structural encoding Attention + GNN	WL and *K*-hop	Attribute reconstruction Structure reconstruction
[283]	Static graphs	Structural encoding	WL, *K*-hop	Structure reconstruction
[29]	Heterogeneous graphs	Structural encoding Edge channels	Laplacian matrix	Structure reconstruction
SAN [284]	Heterogeneous graphs	Structural encoding Edge channels	Laplacian matrix	Structure reconstruction
Grover [100]	Heterogeneous graphs	MP + Attention	*k*-hop	Feature prediction Motif prediction
Mesh Graphormer [99]	Static graphs	Attention + CGNNs	*k*-order proximity	Graph reconstruction
HGT [285]	Heterogeneous graphs	Attention+ MP	Meta-paths	Graph reconstruction
UGformer [61]	Heterogeneous graphs	Attention +GNN	1st-order proximity	Graph reconstruction
StA-PLAN [286]	Heterogeneous graphs	Attention matrix	1st-order proximity	Structure reconstruction
NI-CTR [287]	Heterogeneous graphs	Attention matrix	Subgraph sampling	Structure reconstruction
[101]	Heterogeneous graphs	MP + Attention	1-hop neighbors	Structure reconstruction
[66]	Heterogeneous graphs	Attention + MP	Subgraph	Masked label prediction
Gophormer [46]	Heterogeneous graphs	Attention matrix	Ego-graph *k*-order proximity	Node classification
Graformer [47]	Knowledge graphs	Edge channels	SPD	Structure reconstruction
Graphormer [67]	Homogeneous graphs	Edge channels	SPD	Structure reconstruction
EGT [30]	Homogeneous graphs	Edge channels	SPD	Structure reconstruction

**Table 16:** A summary of hyperbolic models.

Models	Graph Types	Hyperbolic Models	Model Types
[70]	Homogeneous graphs	Poincaré disk	Shallow models
[102]	Homogeneous graphs	Lorentz model	Shallow models
[71]	Heterogeneous graphs	Poincaré disk	Shallow models
[293]	Homogeneous graphs	Poincaré disk	Convolutional GNNs
[294]	Homogeneous graphs	Poincaré disk, Lorentz model	GNNs
LGCN [69]	Homogeneous graphs	Lorentzian model	GNNs
[68]	Homogeneous graphs	Gyrovector model	GAT

**Table 17:** A summary of Gaussian embeddings models.

Model	Graph Type	Model	Structure Preservation
VGAE [72]	Homogeneous graphs	Autoencoder-based GCNs	Random-walk sampling
DVNE [298]	Homogeneous graphs	Autoencoder	1-order, 2-order proximity
[104]	Heterogeneous graphs	MLP	Meta-path
[23]	Homogeneous graphs	Autoencoder	*k*-order proximity
KG2E [299]	Knowledge graphs	Triplet score (h,t,r)	1-order proximity

**Table 18:** A summary of benchmark datasets for graph embedding models. # Nodes and # Edges indicate the number of nodes and edges in graphs, respectively.

Dataset	Graph Type	Category	# Nodes	# Edges
Cora [406]	Homogeneous graph	Citation network	2808	5429
Citeseer [407]	Homogeneous graph	Citation network	3312	4732
Reddit [22]	Homogeneous graph	Social network	232,965	114,615,892
PubMed [406]	Homogeneous graph	Citation network	19,717	44,338
Wikipedia [406]	Homogeneous graph	Webpage	2405	17,981
DBLP [408]	Homogeneous graph	Citation network	781,109	4,191,677
BlogCatalog [408]	Homogeneous graph	Social network	10,312	333,983
Flickr [1]	Homogeneous graph	Social network	80,513	5,899,882
Facebook [409]	Homogeneous graph	Social network	4039	88,234
PPI [22]	Homogeneous graph	Biochemical network	56,944	818,716
MUTAG [410]	Homogeneous graph	Biochemical network	27,163	148,100
PROTEIN [411]	Homogeneous graph	Biochemical network	43,500	162,100
Wiki	Homogeneous graph	Webpage	4,780	184,81 K
YouTube	Homogeneous graph	Video streaming	1,130,000	2,99 M
DBLP [412]	Heterogeneous graph	Citation network	Author (A): 4057 Paper (P): 14,328 Term (T): 7723 Venue (V): 20	A-P: 19,645 P-T: 85,810 P-V: 14,328
ACM [412]	Heterogeneous graph	Citation network	Paper (P): 4019 Author (A): 7167 Subject (S): 60	P-P: 9615 P-A: 13,407 P-S: 4019
IMDB [412]	Heterogeneous graph	Movie reviews	Movie (M): 4278 Director (D): 2081 Actor (A): 5257	M-D: 4278 M-A: 12,828
DBIS [413]	Heterogeneous graph	Citation network	Venues (V): 464 Authors (A): 5000 Publication (P): 72,902	-
BlogCatalog3 [414]	Heterogeneous graph	Social network	User: 10,312 Group: 39	348,459
Yelp [415]	Heterogeneous graph	Social media	User: 630,639 Business: 86,810 City: 10 Category: 807	-
U.S. Patents [180]	Heterogeneous graph	Patent, Trademark Office	Patent: 295,145 Inventor: 293,848 Assignee: 31,805 Class: 14	-
UCI [416]	Dynamic graph	Social network	1899	59,835
DNC [416]	Dynamic graph	Social network	2029	39,264
Epinions [417]	Dynamic graph	Social media	6224	19496
Hep-th [418]	Dynamic graph	Citation network	34,000	421,000
Auto Systems [419]	Dynamic graph	BGP logs	6000	13,000
Enron	Dynamic graph	Email network	87,000	1,100,000
StackOverflow [420]	Dynamic graph	Question&Answer	14,000	195,000
dblp [408]	Dynamic graph	Citation network	90,000	749,000
Darpa [421]	Dynamic graph	Computer network	12,000	22,000

**Table 19:** A summary of libraries of graph embeddings models. The accessibility of URLs for open-source repositories of the libraries have been checked on 16 April 2023.

Library	URL	Platform	Model
PyTorch Geometric (PyG) [426]	https://github.com/pyg-team/pytorch_geometric	PyTorch	Various GNN models and basic graph deep-learning operations
Deep Graph Library (DGL) [427]	https://github.com/dmlc/dgl	TensorFlow, PyTorch	Various GNN models and basic graph deep-learning operations
OpenNE	https://github.com/thunlp/OpenNE/tree/pytorch	TensorFlow, PyTorch	Shallow models: DeepWalk, Node2Vec, GAE, VGAE, LINE, TADW, SDNE, HOPE, GraRep, GCN
CogDL [428]	https://github.com/THUDM/cogdl	TensorFlow, PyTorch	Various GNN models
Dive into Graphs (DIG) [429]	https://github.com/divelab/DIG	PyTorch	Various GNN models and research-oriented studies (Graph generation, Self-supervised learning (SSL), explainability, 3D graphs, and graph out-of-distribution).
Graphvite [430]	https://github.com/deepgraphlearning/graphvite	Python	DeepWalk, LINE, Node2Vec, TransE, RotatE, and LargeVis.
GraphLearn [431]	https://github.com/alibaba/graph-learn	Python	Various GNN models, the framework can support the sampling batch graphs or offline training process.
Connector	https://github.com/NSLab-CUK/connector	Pytorch	Various shallow models and GNN models.

## Data Availability

Data sharing not applicable.

## Appendix A. Open-Source Implementations

We deliver a summary of open-source implementations of graph embedding models described in Section 3. Table A1 provides open-source implementations of graph kernels (Section 3.1), matrix factorization-based (Section 3.2), and shallow models (Section 3.3). Table A2 provides open-source implementations of deep-learning-empowered models (Section 3.4). Table A3 presents open-source implementations of non-Euclidean models (Section 3.5).

**Table A1:** A summary of open-source implementations of graph kernels, matrix factorization-based, and shallow models, which are introduced in Section 3.1, Section 3.2, and Section 3.3, respectively. The accessibility of URLs for the open-source implementations have been checked on 16 April 2023.

Model	Category	URL
[84]	Graph kernels	https://github.com/BorgwardtLab/WWL
[111]	Graph kernels	https://github.com/hasanmdal/GraTFEL-Source
[113]	Graph kernels	https://github.com/ferencberes/online-node2vec
[36]	Graph kernels	https://github.com/yeweiysh/MSPG
[35]	Graph kernels	https://github.com/haidnguyen0909/weightedWWL
[34]	Graph kernels	https://github.com/chrsmrrs/glocalwl
[7]	Matrix factorization-based models	https://github.com/andompesta/ComE.git
GLEE [122]	Matrix factorization-based models	https://github.com/DefuLian/lightne
GraRep [15]	Matrix factorization-based models	https://github.com/ShelsonCao/GraRep
HOPE [5]	Matrix factorization-based models	https://github.com/ZW-ZHANG/HOPE
ProNE [43]	Matrix factorization-based models	https://github.com/THUDM/ProNE
TADW [131]	matrix factorization-based models	https://github.com/thunlp/tadw
PME [138]	matrix factorization-based models	https://github.com/TimDettmers/ConvE
DeepWalk [14]	Shallow models	https://github.com/phanein/deepwalk
Node2vec [4]	Shallow models	https://github.com/aditya-grover/node2vec
Node2Vec+ [148]	Shallow models	https://github.com/krishnanlab/node2vecplus_benchmarks
Struct2Vec [21]	Shallow models	https://github.com/leoribeiro/struc2vec
Gat2Vec [154]	Shallow models	https://github.com/snash4/GAT2VEC
NME [160]	Shallow models	https://github.com/HKUST-KnowComp/MNE
[162]	Shallow models	http://www3.ntu.edu.sg/home/aspatra/research/Yongjin_BI2010.zip
[166]	Shallow models	https://github.com/higd963/Multi-resolution-Network-Embedding
EvoNRL [170]	Shallow models	https://github.com/farzana0/EvoNRL
STWalk [172]	Shallow models	https://github.com/supriya-pandhre/STWalk
[173]	Shallow models	https://github.com/urielsinger/tNodeEmbed
LINE [16]	Shallow models	https://github.com/tangjianpku/LINE
DNGR [196]	Shallow models	https://github.com/ShelsonCao/DNGR
TriDNR [441]	Shallow models	https://github.com/shiruipan/TriDNR
[188]	Shallow models	https://github.com/fuguoji/event2vec

**Table A2:** A summary of open-source implementations of deep-learning-empowered graph embedding models discussed in Section 3.4. The accessibility of URLs for the open-source implementations have been checked on 16 April 2023.

Model	Category	URL
SDNE [50]	Graph autoencoder	http://nrl.thumedia.org/structural-deep-network-embedding
[57]	Graph autoencoder	https://github.com/minkky/Graph-Embedding
Topo-LSTM [49]	Graph autoencoder	https://github.com/vwz/topolstm
GCN [18]	Spectral GNNs	https://github.com/tkipf/gcn
[211]	Spectral GNNs	https://github.com/ZhuangCY/Coding-NN
[214]	Spectral GNNs	https://github.com/Tiiiger/SGC
FastGCN [52]	Spatial GNNs	https://github.com/matenure/FastGCN
GraphSAINT [53]	Spatial GNNs	https://github.com/GraphSAINT/GraphSAINT
Hi-GCN [54]	Spatial GNNs	https://github.com/haojiang1/hi-GCN
GIN [24]	Spatial GNNs	https://github.com/weihua916/powerful-gnns
ST-GDN [220]	Spatial GNNs	https://github.com/jill001/ST-GDN
SACNNs [223]	Spatial GNNs	https://github.com/vector-1127/SACNNs
[227]	Spatial GNNs	https://github.com/rdevon/DIM
[229]	Spatial GNNs	https://github.com/dinhinfotech/PGC-DGCNN
PHC-GNNs [233]	Spatial GNNs	https://github.com/bayer-science-for-a-better-life/phc-gnn
Dyn-GRCNN [236]	Spatial GNNs	https://github.com/RingBDStack/GCNN-In-Traffic
DMGI [243]	Spatial GNNs	https://github.com/pcy1302/DMGI
EvolveGCN [245]	Spatial GNNs	https://github.com/IBM/EvolveGCN
GAT [19]	Attentive GNNs	https://github.com/PetarV-/GAT
GATv2 [58]	Attentive GNNs	https://github.com/tech-srl/how_attentive_are_gats
SuperGAT [258]	Attentive GNNs	https://github.com/dongkwan-kim/SuperGAT
GraphStar [256]	Attentive GNNs	https://github.com/graph-star-team/graph_star
HAN [25]	Attentive GNNs	https://github.com/Jhy1993/HAN
[260]	Attentive GNNs	https://github.com/AvigdorZ/ADaptive-Structural-Fingerprint
DualHGCN [264]	Attentive GNNs	https://github.com/xuehansheng/DualHGCN
MHGCN [266]	Attentive GNNs	https://github.com/NSSSJSS/MHGCN
[334]	Attentive GNNs	https://github.com/Blair1213/DDKG
Graformer [47]	Graph transformer	https://github.com/mnschmit/graformer
Graph-Bert [63]	Graph transformer	https://github.com/jwzhanggy/Graph-Bert
EGT [30]	Graph transformer	https://github.com/shamim-hussain/egt
UGformer [61]	Graph transformer	https://github.com/daiquocnguyen/Graph-Transformer
Graphormer [67]	Graph transformer	https://github.com/microsoft/MeshGraphormer
Yao et al. [101]	Graph transformer	https://github.com/QAQ-v/HetGT
[282]	Graph transformer	https://github.com/jcyk/gtos
[29]	Graph transformer	https://github.com/graphdeeplearning/graphtransformer
SAN [284]	Graph transformer	https://github.com/DevinKreuzer/SAN
HGT [285]	Graph transformer	https://github.com/UCLA-DM/pyHGT
NI-CTR [287]	Graph transformer	https://github.com/qwerfdsaplking/F2R-HMT

**Table A3:** A summary of open-source implementations of non-Euclidean graph embedding models, which are described in Section 3.5. The accessibility of URLs for the open-source implementations have been checked on 16 April 2023.

Model	Category	URL
[70]	Hyperbolic space	https://github.com/facebookresearch/poincare-embeddings
[293]	Hyperbolic space	http://snap.stanford.edu/hgcn/
[294]	Hyperbolic space	https://github.com/facebookresearch/hgnn
Graph2Gauss [23]	Gaussian embedding	https://github.com/abojchevski/graph2gauss
